# Supplementary material for: Exploring emotional well-being, spiritual, religious and personal beliefs and telomere length in chronic pain patients—A pilot study with cross-sectional design
Source: PLoS One. 2024 Sep 4;19(9):e0308924. doi: 10.1371/journal.pone.0308924 (PMC11373805; doi:10.1371/journal.pone.0308924)
Supplement: Attachment — Submitted filename: PONE-D-23-08421_R1_reviewer_RH.pdf [file pone.0308924.s002.pdf]

PLOS ONE

Exploring emotional well-being, spiritual, religious and personal beliefs and telomere length in chronic pain patients - A pilot study with cross-sectional design

--Manuscript Draft--

**Manuscript Number:**	PONE-D-23-08421R1
**Article Type:**	Research Article
**Full Title:**	Exploring emotional well-being, spiritual, religious and personal beliefs and telomere length in chronic pain patients - A pilot study with cross-sectional design
**Short Title:**	Pain, Emotional Well Being and Telomeres
**Corresponding Author:**	Torkel Falkenberg<br>Karolinska Institutet<br>Stockholm, SWEDEN
**Keywords:**	Chronic pain, Emotional health, Spirituality, Telomere length, Telomerase activity, Integrative Health Care
**Abstract:**	Introduction : Living with chronic pain is associated with substantial suffering and high societal costs. Patient reported outcomes (PROM's) and effects on cellular ageing should be considered in pain management. The aim of this study was to explore correlations of PROM's and cellular ageing (telomere length [TL] and telomerase activity [TA] measures) amongst patients with chronic non-malignant pain. Methods: This was an explorative pilot study with cross-sectional design and recruitment was done at two pain rehabilitation facilities in Sweden, with inpatient setting/integrative care and outpatient setting/multimodal care, respectively. Eighty-four patients were enrolled by referral to pain rehabilitation in Sweden. Main outcome measures collected after admission in addition to TL and TA were the following PROMs: Numerical Rating Scale, Chronic Pain Acceptance Questionnaire, Hospital Anxiety and Depression scale, Five Facets Mindfulness Questionnaire, WHO Quality of Life - Spiritual, Religious and Personal Beliefs and EuroQol 5 Dimensions. Results: All the PROM's showed evidence of poor health status. TL correlated negatively with Hospital Anxiety and Depression (r = -.219, p = .047) and positively with WHO Quality of Life -Spiritual, Religious and Personal Beliefs (r = .224, p = .052). TL did not correlate with any of the pain measures. TA correlated positively with pain spread (r = .222, p = .049). A mediation of the direct effect of spiritual well-being on TL by anxiety and depression could be shown (b = 0.008; p = .045). Conclusion: The correlations between TL and spiritual well-being and anxiety and depression suggest some importance of emotional and spiritual dimensions in pain management, with implications for cellular aging, which needs further confirmation.
**Order of Authors:**	Linn Rönne Petersen
	Maria Niemi
	Harald Walach
	catharina lavebratt
	Liu L Yang
	Björn Gerdle
	Bijar Ghafouri
	Torkel Falkenberg
**Response to Reviewers:**	Response to Reviewers<br><br>Torkel Falkenberg<br>Karolinska Institutet<br>Dept. of Neurobiology, Care Sciences & Society<br>Alfred Nobels allé 23, 141 52 Huddinge<br>Sweden

Powered by Editorial Manager® and ProduXion Manager® from Aries Systems Corporation

PONE-D-23-08421
Exploring emotional well-being, personal, spiritual and religious beliefs and telomere length in chronic pain patients - A pilot study with cross-sectional design

May 2023,
Dear Editor,

Thank you for considering our manuscript for publication in PLOS ONE and please find our revisions and comments that addresses the points raised during the review process as described below.

Reviewer #1:
*The study explores a fascinating hypothesis, around the relationship between several psychological states and telomere length. I should note beforehand that my expertise lies in spirituality and psychology, not in biology, so there is a lot about this study that I cannot evaluate. This may also have led to some misunderstandings on my part, for which I hope the authors will forgive me.
-Thank you so much for appreciating our work.

*My main question around this study has to do with its rationale. A minor point on this is on page 4, when the authors state that 'sometimes TA is a signal for repair activity, or an indicator of a lack of such activity'. This immediately raised the question for me what then the relevance and validity of this measure is. Why is it included in this study? When is or isn't it an indicator for (non-)activity? Perhaps the authors can elaborate on this a little.

-Thank you for pointing this out. We have tried to clarify this and changed the text to: "Changes in TA enzyme levels have implications for telomere length and protection of the chromosomes [18, 19], making it suitable for examining more immediate effects in time of for example certain lifestyle factors or health-promoting behaviors [20].
*Also, from the introduction it doesn't become clear what the rationale behind the study is: Why do the authors expect TL and TA to relate to the mentioned PROs?

- We have made the rationale clearer in the introduction. In addition, we point to research that has linked TL to chronic pain and cellular ageing, whereas reports on pain and TA are lacking and hence the inclusion in this study in order to learn more about the possible role of TA levels and pain.

*What's more, from the methods section I understand that this study is part of a larger clinical trial on the effects of anthroposophical integrative care (AIC) or standard care (SC) on TA and TL, with the PROs being secondary outcome variables in this trial. What then, is the reason behind this current study?

-Thank you for addressing this. Indeed, this is the baseline reporting (before care programs are provided). In the rationale we describe the need for research aiming to investigate correlations between emotional well-being, pain experiences and cellular aging in the group of chronic pain patients. Provided such correlations exists, this could underpin "new ways of developing and evaluating chronic pain interventions" for this group of patients.

*The research question of this manuscript is also not mentioned in the trial registration. Might it be that the authors were examining bivariate correlations to check their data, stumbled upon the associations reported here and decided to publish about it? If so, the authors should at least make this clear.

-The reviewer is correct. At the time of the trial registration, we did not foresee baseline reporting as a self-standing paper. However, when confronted with the massive amount of data in this project, we realized the need for detailing the pain patients reported PROM's in relation to TA and TL before they were subjected to care programs, since such information may help the development and evaluation of current pain management.

*Assuming for now that the authors have a clear rationale for examining the research

question in this manuscript, I have some further questions for clarification (in order of appearance, not in order of importance):
* What were the arguments for the inclusion and exclusion criteria? Specifically, the county councils and the comorbities (which are also reported much more specifically in the trial registration).

-The idea was to be as pragmatic as possible and authentic to the irl clinical health care reality both patients and clinicians experience on a daily basis. Hence, we were aiming for external validity, rather than internal validity, and inclusion criterions were deliberately broad. The ambition was to describe this cohort of patients as correctly as possible, and hence their comorbidities were of interest, alongside with many other reported outcomes. The effect on all outcomes of the different care programs will be reported separately.
* Why were the different parameters in the blood samples examined? What role do these play in the current study?
-These are important to monitor since routine blood sample parameters beyond normal reference intervals could hypothetically affect our outcomes. However, at group level, this was not the case since all routine blood test results were within normal reference intervals according to Swedish standards at Karolinska University Laboratory. We do elaborate a little on this: “Interestingly, the blood samples did not deviate from normal reference intervals indicating that patients with severe and long-lasting pain paradoxically appear “perfectly healthy”. However, more complex proteomics or pro-inflammatory markers have been shown to deviate in chronic pain patients and correlate with other health aspects such as HAD scores [54]”. The latter markers were not examined in this study.

* Why were both partial correlations and linear regression analysis examined? Did the first influence which variables were included in the latter? Or was there some other reason?

Thank you for noting this – indeed, the linear regression analyses are no longer reported in the paper due to the redundancy you describe, and we have thus removed that sentence form the statistical analysis description. Instead, we have added a description of the ANCOVA analysis result which is reported: “In addition, we used an analytic approach similar to Hassett et al [5], where the groups were divided into “high pain/low pain” and “high HAD score/low HAD score” (the group mean score was used as cut-off), and ANCOVA analyses adjusted for age were conducted with TL and TA as dependent variables.”

* Were no control variables included in the linear regression analysis, particularly gender and level of education? If so, why not? [I do want to complement the authors for controlling for multiple testing]
We do not report linear regression analyses in this paper and have removed the description form the statistical analysis section (see above).

How were differences in age, pain duration and pain spread between the samples from the study sites handled?

*As we do not compare study sites in this study, we have not taken into account the differences in baseline variables in the study site samples in the analysis.

* What does it mean for the study that neither TL nor TA were correlated to any of the pain measures?

-We do elaborate on this: “Another finding is the absence of correlation between TL and the pain measures selected in this study. This is surprising since others have found statistically significant correlations between TL and pain measures [5, 21-24]. In contrast, TA had a positive relationship with pain spread, i.e. high TA was associated with higher pain spread. But again, this correlation is not large enough to reach a convincing significance level with the sample size we had available. This is ambiguous since TL is negatively associated with pain spread to a notable degree”.

The fact that TL was not linked to pain measures but rather to existential measures is something we address in the summary: “In summary, what ultimately correlates with

the genomic integrity within this sample of chronic pain patients is neither quantitative pain measures or scales, nor index numbers generated for health economy calculation purposes, but instead anxiety, depression and personal, spiritual and religious beliefs".

Our findings hence challenge the idea that the big focus of health care on the pain itself might not be what really is important for the well being of the patients, but rather their existential situation and experiences which seem also to be linked to vital parameters such as the longevity of their DNA.

* Why was an ANCOVA performed for the HADS and not the WHOQOL?

We agree, that it makes sense to run the same analysis and report results for WHOQOL-SRPB as well – we have now added this.

* What theoretical considerations underlie the test for the mediation effect?

Thank you for addressing this. As we state in the article, our mediation model is intuitive, but far from definitive and that one can argue that mediation is only demonstrated according to the original Baron and Kenny approach, but not according to more recent discussions, which would require an independent interaction term to be significant. However, we nevertheless think that this potential mediation might be interesting for other researchers to scrutinize in more detail. Given the explorative design of our study we hope the reviewer agree with this.

* In the discussion section the authors discuss many findings that do not pertain to the research question. What is the purpose of this discussion?

Thank you for pointing this out. We agree that some elaborations may seem a little out of scope. However, we take the opportunity in the discussion to address gender, pain management recommendations and the global opioid crisis since they are all dimensions which the typical chronic pain patient experience with implications for their individual care programs where emotional and spiritual dimensions are lacking.

* What is the connection of the study with opioid use (p.22-23)? This was not included in the study was it?

- We do report drug use in the result section and state in the discussion that: "despite the relation to global morbidity, mortality and doubtful long-term effectiveness, 24 % of the participants in our sample of chronic pain patients had opioids prescribed for their pain". We think that this merits discussion of opioid use as well as the global crisis around pain management.

* Overall, I think the authors present an interesting finding, based on acceptable methodology, that certainly warrants further study. However, the background of the study in insufficiently clear and needs a more sound rationale.

- Thank you and we hope the changes and clarifications we have made have improved the manuscript to the reviewer's satisfaction.

Reviewer #2: Dear authors, thank you for your efforts. Your study covers a relevant topic and an important attempt to promote more holistic non-pharmacological treatments in chronic pain patients. Your methodology is sound (scales used, handling of biological materials) but the sample size is very small and relevant confounders have not been considered (tobacco and alcohol as stated by yourself, other lifestyle factors as exercise and nutrition, or patients medication specially anti-inflammatory drugs). This makes the results, specially the associations with TL/TA rather speculative and hypothetical.

Thank you for appreciating our efforts in this challenging study and we agree that research like this is needed to find new ways to improve existential care of patients in need, such as chronic pain patients given their large suffering.

We agree that the sample size is rather small, however given the complexity of this

	project with measures from cell to existential health it was not possible to enroll a larger patient basis. In the article we state that: "Because of its exploratory nature, no sample size was calculated but a minimum of 70 enrolled patients was strived for, which allows to document an effect size of r = .3 with 72% power and reaches a power of 80% with r = .32. These are the ranges of correlations that were deemed likely and clinically important".<br><br>We do address this as a limitation in the discussion and also state that: "The size of the correlations we found were rather small, between r = .2 and .3. Because of the small sample size and the comparatively large number of correlations, inherent in an exploratory project such as this one, we would not want to draw any definitive conclusions".<br><br>Hence, we agree that the findings of this exploratory project, notably the weak correlation to TL, need replication in larger clinical trials. In such future trials, the limitation of potential confounders apart from BMI, age or sex such as the one mentioned by the reviewer including tobacco or alcohol use should be considered.<br><br>We have added the limitations suggested by the reviewer to the limitation discussion: "Indeed, levels of exercise and nutrition and patients medication should also be considered".<br><br>*Associations between spiritual und religious beliefs and mental health (anxiety and depression) are well documented and have been found in several studies and different contexts (pain, cardiac rehabilitation, coping with disease).<br><br>-Thank you for addressing this, and we have extended the discussion to include an extension on this: "Studies have repeatedly established positive links between religion, spirituality and health where for example religious involvement is correlated with better mental health in the areas of depression, substance abuse, and suicide (reference 1). It has also been shown that cardiovascular and cortisol stress responses of a sample of university students can be predicted by their secularity, religiosity, spirituality, and existential search (reference 2)".<br><br>1.Bonelli, R.M., Koenig, H.G. Mental Disorders, Religion and Spirituality 1990 to 2010: A Systematic Evidence-Based Review. J Relig Health 52, 657–673 (2013). https://doi.org/10.1007/s10943-013-9691-4<br>2.Schnell, T., Fuchs, D. & Hefti, R. Worldview Under Stress: Preliminary Findings on Cardiovascular and Cortisol Stress Responses Predicted by Secularity, Religiosity, Spirituality, and Existential Search. J Relig Health 59, 2969–2989 (2020). https://doi.org/10.1007/s10943-020-01008-5<br><br><br>6. PLOS authors have the option to publish the peer review history of their article (what does this mean?). If published, this will include your full peer review and any attached files.<br><br>If you choose "no", your identity will remain anonymous but your review may still be made public.<br><br>Do you want your identity to be public for this peer review? For information about this choice, including consent withdrawal, please see our Privacy Policy.<br>Reviewer #1: Yes: Anja Visser<br>Reviewer #2: Yes: René Hefti M.D.<br><br>Thank you for your fine work in reviewing this manuscript and for your contributions to this research field.<br><br>Sincerely yours,<br>Torkel Falkenberg, on behalf of all the co-authors
**Additional Information:**	
**Question**	**Response**

Powered by Editorial Manager® and ProduXion Manager® from Aries Systems Corporation

**Financial Disclosure**<br><br>Enter a financial disclosure statement that describes the sources of funding for the work included in this submission. Review the submission guidelines for detailed requirements. View published research articles from *PLOS ONE* for specific examples.<br><br>This statement is required for submission and **will appear in the published article** if the submission is accepted. Please make sure it is accurate.<br><br>**Unfunded studies**<br>Enter: *The author(s) received no specific funding for this work.*<br><br>**Funded studies**<br>Enter a statement with the following details:<br>• Initials of the authors who received each award<br>• Grant numbers awarded to each author<br>• The full name of each funder<br>• URL of each funder website<br>• Did the sponsors or funders play any role in the study design, data collection and analysis, decision to publish, or preparation of the manuscript?<br>• **NO** - Include this sentence at the end of your statement: *The funders had no role in study design, data collection and analysis, decision to publish, or preparation of the manuscript.*<br>• **YES** - Specify the role(s) played.<br><br>* typeset	This work was supported by Ekhagastiftelsen (TF), KI Research School in Health Care Sciences (LRP), Gyllenbergsstiftelsen (TF;LRP), Integrative Care Science Center (TF;LRP;MN), Vidarstiftelsen (TF) and Mahle stiftung (TF). The funders had no role in study design, data collection and analysis, decision to publish, or preparation of the manuscript.
**Competing Interests**<br><br>Use the instructions below to enter a competing interest statement for this submission. On behalf of all authors, disclose any competing interests that could be perceived to bias this work—acknowledging all financial support and any other relevant financial or non-financial competing interests.<br><br>This statement is required for submission	The authors have declared that no competing interests exist.

and **will appear in the published article** if the submission is accepted. Please make sure it is accurate and that any funding sources listed in your Funding Information later in the submission form are also declared in your Financial Disclosure statement.<br><br>View published research articles from *PLOS ONE* for specific examples.<br><br>**NO authors have competing interests**<br><br>Enter: *The authors have declared that no competing interests exist.*<br><br>**Authors with competing interests**<br><br>Enter competing interest details beginning with this statement:<br><br>*I have read the journal's policy and the authors of this manuscript have the following competing interests: [insert competing interests here]*<br><br>* typeset	
**Ethics Statement**<br><br>Enter an ethics statement for this submission. This statement is required if the study involved:<br><br>• Human participants<br>• Human specimens or tissue<br>• Vertebrate animals or cephalopods<br>• Vertebrate embryos or tissues<br>• Field research<br><br>Write "N/A" if the submission does not require an ethics statement.<br><br>General guidance is provided below. Consult the submission guidelines for detailed instructions. **Make sure that all information entered here is included in the Methods section of the manuscript.**	The study was ethically approved by the Regional Ethics Committee in Stockholm (EPN 2014/953-31/1). The study was preregistered (ClinicalTrials.gov Identifier NCT02459639). After oral and written information, participants could make an informed written decision whether to participate or not with no implications for their care.

**Format for specific study types**<br><br>**Human Subject Research (involving human participants and/or tissue)**<br>• Give the name of the institutional review board or ethics committee that approved the study<br>• Include the approval number and/or a statement indicating approval of this research<br>• Indicate the form of consent obtained (written/oral) or the reason that consent was not obtained (e.g. the data were analyzed anonymously)<br><br>**Animal Research (involving vertebrate animals, embryos or tissues)**<br>• Provide the name of the Institutional Animal Care and Use Committee (IACUC) or other relevant ethics board that reviewed the study protocol, and indicate whether they approved this research or granted a formal waiver of ethical approval<br>• Include an approval number if one was obtained<br>• If the study involved *non-human primates*, add *additional details* about animal welfare and steps taken to ameliorate suffering<br>• If anesthesia, euthanasia, or any kind of animal sacrifice is part of the study, include briefly which substances and/or methods were applied<br><br>**Field Research**<br><br>Include the following details if this study involves the collection of plant, animal, or other materials from a natural setting:<br>• Field permit number<br>• Name of the institution or relevant body that granted permission	
**Data Availability**<br><br>Authors are required to make all data underlying the findings described fully available, without restriction, and from the time of publication. PLOS allows rare exceptions to address legal and ethical concerns. See the PLOS Data Policy and FAQ for detailed information.	No - some restrictions will apply

A Data Availability Statement describing where the data can be found is required at submission. Your answers to this question constitute the Data Availability Statement and **will be published in the article**, if accepted.<br><br>**Important:** Stating 'data available on request from the author' is not sufficient. If your data are only available upon request, select 'No' for the first question and explain your exceptional situation in the text box.<br><br>Do the authors confirm that all data underlying the findings described in their manuscript are fully available without restriction?	
**Describe where the data may be found in full sentences. If you are copying our sample text, replace any instances of XXX with the appropriate details.**<br><br>• If the data are **held or will be held in a public repository**, include URLs, accession numbers or DOIs. If this information will only be available after acceptance, indicate this by ticking the box below. For example: *All XXX files are available from the XXX database (accession number(s) XXX, XXX.)*.<br>• If the data are all contained **within the manuscript and/or Supporting Information files**, enter the following: *All relevant data are within the manuscript and its Supporting Information files.*<br>• If neither of these applies but you are able to provide **details of access elsewhere**, with or without limitations, please do so. For example:<br><br>*Data cannot be shared publicly because of [XXX]. Data are available from the XXX Institutional Data Access / Ethics Committee (contact via XXX) for researchers who meet the criteria for access to confidential data.*<br><br>*The data underlying the results presented in the study are available from (include the name of the third party*	There are ethical restrictions on sharing our de-identified data set publicly since data contain potentially identifying sensitive patient information. The reasons are both the rules on confidentiality, primarily to protect the research subjects, and the GDPR regulations in Sweden. These restrictions are imposed by the institutional legal body thru lawyer Sofia Hallquist Björkegren; sofia.hallquist@ki.se to which data requests may be sent for researchers who meet the criteria for access to confidential data.

Powered by Editorial Manager® and ProduXion Manager® from Aries Systems Corporation

and contact information or URL).<br>• This text is appropriate if the data are owned by a third party and authors do not have permission to share the data.<br><br>* typeset	
Additional data availability information:	Tick here if your circumstances are not covered by the questions above and you need the journal’s help to make your data available.

Powered by Editorial Manager® and ProduXion Manager® from Aries Systems Corporation

Manuscript
Click here to access/download;Manuscript;Revised Telomeres, Pain and Well being PLOS ONE.docx

Title Page

Exploring emotional well-being, personal, spiritual and religious beliefs and telomere length in chronic pain patients - A pilot study with cross-sectional design

Linn Rönne-Petersén, Msc[1] linn.ronne-petersen@ki.se
Maria Niemi, PhD[2] maria.niemi@ki.se
Harald Walach, Professor[3] hw@chs-institute.org
Catharina Lavebratt, Assoc Prof[4] catharina.lavebratt@ki.se
Liu L. Yang, M.D, PhD[4] liu.yang@ki.se
Björn Gerdle, Professor[5] bjorn.gerdle@liu.se
Bijar Ghafouri, Assoc Prof[5] bijar.ghafouri@liu.se
Torkel Falkenberg, Assoc Prof[1]* torkel.falkenberg@ki.se +46706183868

*Corresponding author

AFFILIATIONS:
[1]*Deparment of Neurobiology, Care sciences and Society, Division of Nursing 2, Karolinska Institutet, 171 77, Stockholm, Sweden.*
[2]*Department of Global Public Health, K9 FHE Dalman, Karolinska Institutet, 171 77 Stockholm, Sweden*
[3]*Change Health Science Institute, Berlin, Germany*
[4]*Department of Molecular Medicine and Surgery, Karolinska Institutet and Center for Molecular Medicine, 171 76 Stockholm, Sweden*
[5]*Pain and Rehabilitation Centre, and Department of Health, Medicine and Caring Sciences, Linköping University, 581 83 Linköping, Sweden*

ABSTRACT

Introduction: Living with chronic pain is associated with substantial suffering and high societal costs. Patient reported outcomes (PROM's) and effects on cellular ageing should be considered in pain management. The aim of this study was to explore correlations of PROM's and cellular ageing (telomere length [TL] and telomerase activity [TA] measures) amongst patients with chronic non-malignant pain. *Methods*: This was an explorative pilot study with cross-sectional design and recruitment was done at two pain rehabilitation facilities in Sweden, with inpatient setting/integrative care and outpatient setting/multimodal care, respectively. Eighty-four patients were enrolled by referral to pain rehabilitation in Sweden. Main outcome measures collected after admission in addition to TL and TA were the following PROMs: Numerical Rating Scale, Chronic Pain Acceptance Questionnaire, Hospital Anxiety and Depression scale, Five Facets Mindfulness Questionnaire, WHO Quality of Life - Spiritual, Religious and Personal Beliefs and EuroQol 5 Dimensions. *Results*: All the PROM's showed evidence of poor health status. TL correlated negatively with Hospital Anxiety and Depression ($r = -.219$, $p = .047$) and positively with WHO Quality of Life -Spiritual, Religious and Personal Beliefs ($r = .224$, $p = .052$). TL did not correlate with any of the pain measures. TA correlated positively with pain spread ($r = .222$, $p = .049$). A mediation of the direct effect of spiritual well-being on TL by anxiety and depression could be shown ($b = 0.008$; $p = .045$). *Conclusion*: The correlations between TL and spiritual well-being and anxiety and depression suggest some importance of emotional and spiritual dimensions in pain management, with implications for cellular aging, which needs further confirmation.

ClinicalTrials.gov Identifier: NCT02459639

KEYWORDS

Chronic pain, Emotional health, Spirituality, Telomere length, Telomerase activity, Integrative Health Care

1. INTRODUCTION

1.1. Chronic pain as a personal and societal condition

Chronic pain constitutes considerable personal suffering for patients living with it, and poses a great challenge for the healthcare system with high economic impact on society [1, 2]. Chronic pain has been described as an enigmatic phenomenon associated with substantial negative psychosocial factors and physical ill-health [3-5]. With 19-50 % of the European population affected, chronic pain is common, and in the female population the prevalence is at least 10 % higher [2, 6, 7]. Patients' reality of complex health issues is often misunderstood and misinterpreted by the healthcare system, leading to feelings of stigmatization and forcing the patients to seek healthcare repeatedly [8, 9]. Leadely et al [1] conclude that chronic pain is neither sufficiently prioritized in policies nor in budgets. Why such a widespread and incapacitating condition isn't more prioritized seems at large unclear.

1.2. Measuring pain

Despite the complex nature of pain and the subsequent need for a wider approach to study it, most pain research has mainly focused on pain intensity and duration [10]. In contrast, Wideman et al [10] have proposed a comprehensive model for how to integrate the nature of subjectivity and objectivity in pain, while at the same time arguing that we can neither measure the largest part of pain, namely the *pain experience (inner experience)*, since it is non-observable and deeply personal. However, what research and clinicians can quantify are *pain measures* (subjective self-reported or objective non-self-reported), preferably mindful of *what* is being measured, i.e. not pain itself, but a limited part of the pain phenomena.

1.3. Telomeres, cellular ageing and pain

Telomeres are specific, protective structures at the ends of our chromosomes that are crucial for preventing genetic information from being lost during cell division [11]. Shortened telomere length (TL) is considered a predictor for a number of age-related diseases and mortality [12-14], although it is also affected by factors *independent* of chronological age [14-16]. In addition, TL shortening tends to be more the result of cumulative life stress and age-related diseases [17]. The enzyme telomer*ase* functions as a buffer against the cellular aging in the nucleus, steadily re-building the telomeres by adding on base pairs [11, 16]. Changes in TL can take months or years to be detectable, while telomerase activity (TA) is more dynamic and linked to short-term changes. Sometimes TA is a signal for repair activity, or an indicator of a lack of such activity [18, 19], making it suitable for examining more direct effects of for example lifestyle on the cellular ageing process [20].

A handful articles address the association between chronic pain and cellular ageing, whereas reports on pain and measures of TA are lacking. The majority of studies find significant correlations between chronic pain or degenerative tissue (associated with pain) and decreased TL [5, 21-24]. There are also studies not showing significant correlations [25, 26]. Notably, one study observed changes in TL when looking at combinations of pain and high and low stress, arguing that a combined effect of the two may accelerate cellular ageing [26]. It has been estimated that chronic pain shortens life by 4-5 years (as measured in loss of base pairs) and combined with depression it adds up to 6 years [5].

Research aiming to investigate correlations between *pain experiences* and cellular aging could contribute to cater for new ways of developing and evaluating different health care interventions. Multimodal interventions including emotional well-being and spiritual health are

important elements of nursing care and are emerging as potential health services components [27] with hypothetical implications for stress, mental health and pain management [27, 28]

The aim of this study was to explore correlations of Patient Reported Outcome Measurements [PROM's] and markers of cellular ageing amongst patients with chronic non-malignant pain. We used PROM's of clinical pain, physical and emotional functioning, personal, spiritual and religious beliefs, mindfulness and quality of life. We used TL and TA as measures of cellular ageing and potential mediators between PROMS and cellular ageing were explored.

2. METHODS

2.1. Participant recruitment

The participants in this study were individuals suffering from chronic pain conditions referred to tertiary specialist centers and enrolled at either The Vidar Clinic, Järna *or* Pain and Rehabilitation Center, University Hospital, Linköping between June 2015 and December 2017. The study was ethically approved by the Regional Ethics Committee in Stockholm (EPN 2014/953-31/1). The study was preregistered (ClinicalTrials.gov Identifier NCT02459639). After oral and written information, participants could make an informed written decision whether to participate or not with no implications for their care. Inclusion criteria were ICD-10 M79, *OR* chronic pain (>3 months) in neck/shoulders and/or lower back or generalized pain (e.g. fibromyalgia), ≥18 years of age, fluent in Swedish, from Stockholm, Sörmland or Östergötland county councils. Comorbidities were allowed except for psychotic illness, addiction, and cancer.

In all, 57 patients at The Vidar Clinic and 138 patients at Linköping were invited to the study, of which 51 respectively 35 patients showed interest to participate. Of these, 51 respectively

33 patients met the inclusion criteria and agreed to participate. The final analysed sample consisted of 84 participating patients. The size of the Vidar Clinic was smaller compared to the Rehabilitation Center, University hospital, Linköping and hence explains the differences in recruitment proportions.

2.2. Data collection

This was an explorative pilot study with cross-sectional design, looking into clinical characteristics and correlations in a sample of persons with chronic non-malignant pain. Measures were collected after admission. Because of its exploratory nature, no sample size was calculated but a minimum of 70 enrolled patients was strived for, which allows to document an effect size of r = .3 with 72% power and reaches a power of 80% with r = .32. These are the ranges of correlations that were deemed likely and clinically important. Patients were recruited from two geographic locations – one with an out-patient setting (Linköping) and one with an in-patient setting (The Vidar Clinic). The same standard protocols for blood sampling were used at both sites, with the patients in a fasting state and where samples were collected after admission. Questionnaires were distributed at the time of the blood sampling. Double data entry was practiced for quality reasons and the completed database was then validated by cross-checking 12% of all data, whereby 0.7% were found to be incorrect due to small systematic errors. Those errors were controlled and adjusted for in the whole dataset before analysing.

2.3. Measurements

The measurements of this study comprised of TA and TL and PROM’s of clinical pain, physical and emotional functioning, personal, spiritual and religious beliefs, mindfulness and quality of life.

In addition, demographics, anthropometrics, blood pressure and blood samples were collected. A BMI between 18.5-24.9 kg/m^2 would be considered normal, as was a blood pressure <140/90 mmHg. The participants' blood samples were taken and sent for standard clinical analysis at Clinical Chemistry Laboratory at Linköping University Hospital and at Karolinska University Laboratory at Södertälje Hospital. The blood count was measured as well as the properties of the erythrocytes, leucocytes, thrombocytes, standard lipid status (triglycerides and cholesterol with quotas) and inflammatory profile (sedimentation rate, C-reactive protein) (data not shown). Also, levels of haemoglobin, glucose, sodium, potassium and creatinine were measured (data not shown). The IMMPACT recommendations for core outcome measurements in chronic pain clinical trials [29] were considered and applied heading-wise, but not strictly followed in regard to the scales used. Some questionnaries were replaced due to being validated in Sweden and others were added due to relevance for this study.

Clinical pain was assessed by means of intensity using the Numerical Rating Scale [NRS] (0 = no pain, 10 = worst possible pain) which is a commonly used instrument, included in the Swedish National Quality Registry for Pain Rehabilitation [SQRP] and recommended by IMMPACT [29], A score <4 translates to mild pain, 4-6 moderate and >7 severe pain. Pain duration (years, months and days) and pain spread was also assessed by means of well established self-report questionaire from SQRP. In addition, the Chronic Pain Acceptance Questionnaire [CPAQ] ($\alpha \geq .80$), with the subscales of *pain willingness* and *activity engagement* was used because of its emphasis on aspects of coping, which is a focus area within pain research [30, 31]. In accordance with recommendations from Dworkin et al [29], patient usage of rescue analgesics (fast-acting medications) were registered. The *physical functioning* was evaluated with the Swedish National Board of Health and Welfare's indicator questions on physical activity [FYS], recently validated

[32], as well as EUROQOL 5 Dimensions-3L [EQ5D](α > .85)[33]. The latter is an established measure of quality of life, including aspects of physical functioning. The EQ5D index value is a single number calculated from the different health states obtained within the questionnaire. This number is commonly ranging from 0 to 1, although negative numbers are possible, and it reflects how good or bad the health state is in comparison with the preferences of a general population in a specific country. The related EQ5D VAS is a single question with the score range 0-100, going from worst possible to best possible health [33]. *Emotional functioning* was measured with the Hospital Anxiety and Depression Scale [HAD Scale] (α > .89), a reliable and valid instrument with subscales measuring *depression* and *anxiety* [34], validated in Sweden [35] and where high values indicate psychological distress [36]. *Participant ratings of global improvement and satisfaction with treatment*, as well as *Symptoms and adverse events* were assessed qualitatively, and will be reported separately.

Personal, spiritual and religious beliefs, are increasingly recognized as a distinctive, important, and cross-cultural concept in quality of life assessments [37]. Hence, in addition to the IMMPACT recommendations we assessed personal, spiritual and religious beliefs using the WHO Quality of Life, Spiritual, Religious and Personal Beliefs (WHOQOL-SRPB) (α = .85) questionnaire in its appropriate Swedish adaptation [38]. The questionnaire has the eight following subscales: *spiritual connection, meaning and purpose in life, experiences of awe and wonder, wholeness and integration, spiritual strength, inner peace, hope and optimism, and faith*. Mindfulness using Five Facets Mindfulness Questionnaire [FFMQ]) (α = .81) was included to complement the WHOQOL-SRPB to assess if improvements in health could be mediated by increased mindfulness. It has five subscales: *non-reactivity, observe, acting with awareness, describe* and *non-judging*. FFMQ is the most frequently used questionnaire for mindfulness [39]

and has been validated in Sweden [40]. The information of number of items and min-max score for each questionnaire is found in Table 3.

2.4. Telomerase measurement

Blood samples used for this study were collected between 2015 and 2017 at the two sites. TA was detected by real-time telomeric repeat amplification protocol (RT-TRAP) [41], with some modifications. In brief, blood samples were drawn in line with Swedish nursing standards, with the participants in a fasting and rested state (minimum 20 minutes). Whole blood was obtained in 8 ml BD Vacutainer® CPT™ Mononuclear Cell Preparation Tube - Sodium Citrate (Becton Dickinson). Thereafter, lymphocytes and monocytes were separated within two hours of sampling, according to manufacturer's protocol. Approximately half of the pelleted cells were lysed by incubation with 120 µl CHAPS (Merck Millipore, including 0.15 units/µl RiboLock [LifeTechnologies, Thermo Fisher Scientific]) on wet ice for 30 min and three short vortexes. Following this initial preparation done locally at laboratories on respective site, the samples were labelled and frozen at -80°C, after which they were transported to Center for Molecular Medicine (CMM) at Karolinska Institutet for storage and further analysis.

Thawed lysate was centrifuged at 4°C at 12000xg for 20 min and the supernatant was transferred to an empty tube. The total protein concentration was measured in the supernatant by the DC Protein Assay (Bio-Rad) and all patients' cell lysates were diluted to equal concentrations in CHAPS (0.95 µg/µl), aliquoted and refrozen at -80°C. An aliquot per sample was thawed and equal amounts of total protein (3.8 µg) from each sample was added to a reaction mix with a total volume of 50 µL containing 1 mM of each dNTP, 20 mM Tris-HCl (pH 8.3), 2.5 mM MgCl2, 63 mM KCl, 0.05% Tween 20, 1 mM EGTA, 8 µmol of each of the primers TS (5'-

AATCCGTCGAGCAGAGTT-3') and ACX (5'- GCGCGG(CTTACC)3CTAACC-3'). HepG2 (hepatic cancer) and ARO (thyroid cancer) cell lines were used as telomerase-positive controls, whereas CHAPS buffer and heat-inactivated samples were used as negative controls. TSR8 is an oligonucleotide with a sequence identical to the TS primer extended with 8 telomeric repeats being $AG(GGTTAG)_7$. Serial dilutions of TSR8 control template were used to generate a standard curve to calculate TA. The serial dilutions were 0.2 amoles/µL, 0.04 amoles/µL, 0.008 amoles/µL, 0.0016 amoles/µL, 0.00032 amoles/µL, and 0.000064 amoles/µL corresponding to 200, 40, 8, 1.6, 0.32 and 0.064 TPG units/µL; TPG is the total product generated, corresponding to the number of TS primers (1 unit equals 10^{-3} amoles or 600 molecules) that are extended with at least 3 TTAGGG repeats by telomerase in the extract in a 30 min incubation at 30°C. The reaction mix was incubated at 30°C for 30 min followed by termination at 95°C for 5 min. Then 8 µL of the telomeric repeat products (i.e. corresponding to 8/50*3.8=0.608 ug total protein) were used for the RT-TRAP assay amplified by 8 µL Power SYBR Green in 384-well plates. The reaction was performed on QuantStudio 7 Flex (Applied Biosystems; Life Technologies; Thermo Fisher Scientific Inc.) with the following conditions: 95°C for 10 min, followed by 36 repeats of 95°C for 20 s, 52°C for 30 s and 72°C for 60 s. Samples, controls and standard curve dilutions were run in triplicate, standard curve and controls on all plates. All samples from each patient were run on the same randomized plate. Efficiency was 99-105%. The mean of the correlation coefficients of the standard curves was > 0.993. The mean coefficients of variation (CV) of intra-plate Ct values for the standard dilutions of the five plates was CV = 0.56%, and the mean inter-plate CV of three inter-plate controls was CV = 1.6%. The detection success rate was 100%. For each supernatant, aliquots are still there with original protein concentration.

2.5. Telomere length measurement

The other half of the pelleted cells, collected and prepared at both sites, were also stored at -80°C until DNA extraction. Genomic DNA was extracted based on the kit [42], but the speed of centrifuge was changed to 6000g and avoid vortex by inverse, and incubate at 37°C for 3 h. DNeasy® Blood & Tissue Kit (Qiagene). DNA concentration was quantified with NanoDrop ND-1000 Spectrophotometer (Nano-Drop Technologies Inc., Wilmington, DE, USA). Relative TL was determined using real-time quantitative polymerase chain reaction (qPCR) according to Cawthon et al's protocol [43] where the relative telomere to single copy gene (T/S) ratios was determined using a standard curve. In brief, each DNA sample (10 ng) was assessed for the telomere (*Tel*) and the single-copy gene (hemoglobin-b, *HGB*) in triplicate within the same 384-well plate, amplified by using Power SYBR Green in 10 µl total reaction volume. The reaction was performed on QuantStudio 7 Flex (Applied Biosystems; Life Technologies, Carlsbad, CA, USA) with the following conditions: 50°C for 2 min, then 95°C for 10 min, followed by 40 repeats of 95°C for 15 s and 60°C for 1 min, followed by a dissociation stage to monitor amplification specificity. The same standard curve of pooled DNA from these patient samples ranging from 80 ng to 0.128 ng, was run on each plate for both genes and was used to determine the quantity of each gene for each sample. This allowed controlling for differences in the efficiencies between that of *Tel* and *HGB.* The gene quantities were then used to determine the T/S ratio for each sample. DNA samples with a Ct standard deviation of ≥ 0.35 between triplicates or a Ct value outside the standard curve were omitted from the analyses. The correlation coefficients of the standard curves were above 0.99 for each primer set and 384-plate. The inter-plate coefficient of variation (CV) of T/S ratio was 9.1% calculated from a patient sample run in seven 384-well plates. The TL analysis detection success rate was 100%. The primer sequences were (written 5'à3'): Tel1:

CGGTTTGTTTGGGTTTGGGTTTGGGTTTGGGTTTGGGTT; Tel2: GGCTTGCCTTACCCTTACCCTTACCCTTACCCTTACCCT; *HGB* Fw: GCTTCTGACACAACTGTGTTCACTAGC; *HGB* Rv: CACCAACTTCATCCACGTTCACC. Samples from all three timepoints per person were assayed in the same 384-well plate.

2.6. Statistical analysis

The software used for analysis was IBM SPSS version 26. Background data are described as means and standard deviations, or frequencies and percentages, and Chi-Square or Fishers exact tests were calculated for descriptive background measures. The TA variable was ln-transformed due to significant skewness. Partial correlations were used to assess how the TL (adjusted for age) and TA correlated with pain intensity, spread and psychological outcomes at baseline. A linear regression analysis was conducted, with age, anxiety and depression and pain intensity as independent variables, to study their impact on the telomere and telomerase variables, which were thus dependent variables in the analyses. A potential mediation model was conducted in an exploratory fashion following the standard mediation model of Baron and Kenny [44]. Direct effects were modeled in univariate regression models. Beta-weights, i.e. partial correlations coefficients were given for the different paths. This model was an ad-hoc decision after we saw the data pattern, because we wanted to explore the data further. It has to be viewed as a preliminary additional analysis to clarify relationships. We mark significance levels according to a p-level of .005 corrected for multiple testing, which is in accordance with a recommended level for new discoveries [45]. But due to the exploratory nature of the study, we do not emphasize significances, but rather effect size, i.e. the extent of the correlation [46].

3. RESULTS

3.1. Sample characteristics

The socio-demographic background variables and pain variables of participants are presented in Table 1. The majority of the participants were female, from Sweden and currently working. Most had upper secondary school or university education. Regarding pain, 82% reported it as constant and high, with an average pain intensity of 7.1 (out of 10) and a mean duration of nearly 12 years. With this amount of pain 60% were taking prescription drugs for pain and various other types of medications. Almost one third of the participants were using herbals and other supplements. In order to assess representativeness of our sample it was compared to the target population and hence Table 2 presents essential characteristics of the sample and target population, with numbers from a report by SQRP [47]. Table 3 presents the differences in participant demographic and pain variables from the different sites, where differences in distributions and means are also presented.

Table 1. *Socio-demographic background and pain variables of participants*

Variable	n	Score	SD
Gender	84		
**Female**	84	85.7%	
**Male**	84	14.3%	
Age	84	47.6	10.3
Country of birth	84		
**Sweden**	84	77.4%	
**Other Nordic countries**	84	3.6%	
**Europe**	84	9.5%	
**Outside Europe**	84	9.5%	
Education	84		
**Primary schooling**	84	7.1%	
**Upper secondary school**	84	64.3%	
**University**	84	22.6%	
**Other**	84	6.0%	
Working	82	87.8%	
Blood pressure	77		
**Systolic**	77	120.4 mmHg	17.3
**Diastolic**	77	76.9 mmHg	10.8
BMI	77	27.6	10.3

ICD-10 diagnosis	84		
**M-diagnoses**	84	66.7%	
**R-diagnoses**	84	19.0%	
**Q-diagnoses**	84	8.0%	
**F-diagnoses**	84	7.1%	
**Other diagnoses**	84	2.4%	
Medication	84		
**Prescription drugs for pain**	84	61%	
**Prescription drugs for other**	84	45.2%	
**Non-prescription drugs for pain**	75	28%	
**Non-prescription drugs for other**	75	10.7%	
**Herbals**	77	26%	
Type of pain	83		
**Constant**	83	81.9%	
**Periodic**	83	18.1%	
Pain duration	81	11.8	10.5
Pain intensity NRS	83	7.1	1.5
Pain spread	83	19.1	9.0

BMI: Body Mass Index, ICD-10: International Classification of Diseases, M-diagnoses: diseases of the musculoskeletal system and connective tissue, R-diagnoses: symptoms, signs and abnormal clinical and laboratory findings not elsewhere classified, Q-diagnoses: congenital malformations, deformations and chromosomal abnormalities, F-diagnoses: Mental, Behavioral and Neurodevelopmental disorders, NRS: numerical rating scale.

Table 2. *Essential sample characteristics in comparison with the target population*

Variable	Sample score	Target population score*
**Age**	47	44
**Gender F/M**	86/14%	79/21%
**Working now**	88%	67%
**Nationality Sweden/other**	81/19%	82/18%
**Senior high school**	64%	41%
**University**	23%	42%
**BMI**	28	27
**Pain duration**	12 years	9 years
**Pain intensity**	7.1	4.1
**Pain spread**	19	15

*Based on Swedish National Quality Registry for Pain Rehabilitation 2020

Table 3: *Differences in demographic and pain variables in the two patient cohorts (Vidar Clinic and Linköping University Hospital)*

	Clinic	N	Mean	p-value (t-test)
**Sex**	Vidar	51	96 (% female)	0.001 (Fisher's exact)
	Linköping	33	70 (% female)	
**age**	Vidar	51	50.50	0.001
	Linköping	33	43.17	
**Pain duration (years)**	Vidar	49	15.39	<0.001
	Linköping	32	6.38	
**Pain intensity**	Vidar	51	6.95	0.43
	Linköping	32	7.22	
**Pain spread**	Vidar	51	22.53	<0.001
	Linköping	32	13.69	

3.2. Patient reported outcome measures

All PROM's were normally distributed. The PROM's for *clinical pain* including pain intensity, pain duration, pain spread, and medication are presented in table 1. All other PROM's, including subscales are presented in Table 4. To better understand the interrelatedness of different PROM's, correlations were calculated amongst all the questionnaires and pain measures and are presented in Table 5.

Table 4. *Patient Reported Outcome Measures including subscales and scoring.*

Questionnaire	n	Items, n	Scoring	Min-max	Mean	Std. Dev
**HAD scale score**	84	14	0-3	0-42	19.17	8.49
HAD scale anxiety	84	7	0-3	0-21	10.13	5.10
HAD scale depression	84	7	0-3	0-21	9.04	4.33
**EQ5D VAS**	82	1	0-100	0-100	40.77	17.37
**EQ5D Index value**	84	5	1-3	5-15	0.26	0.32
**FFMQ score**	80	29	1-5	29-145	91.95	15.12
FFMQ non-reactivity	80	6	1-5	6-30	17.80	3.94
FFMQ observe	80	7	1-5	7-35	24.40	5.02
FFMQ acting with awareness	80	5	1-5	5-25	14.55	3.87

FFMQ describe	80	6	1-5	6-30	19.26	4.80
FFMQ non-judging	80	5	1-5	5-25	16.03	4.22
**CPAQ score**	79	8	0-6	0-48	22.14	7.18
CPAQ pain willingness	79	4	0-6	0-24	11.72	5.34
CPAQ activity engagement	79	4	0-6	0-24	10.42	4.63
**WHOQOL-SRPB score**	75	32	1-5	32-160	92.00	26.44
WHOQOL-SRPB spiritual connection	75	4	1-5	4-20	10.27	4.98
WHOQOL-SRPB meaning and purpose in life	75	4	1-5	4-20	12.85	3.49
WHOQOL-SRPB experiences of awe and wonder	75	4	1-5	4-20	10.52	4.62
WHOQOL-SRPB wholeness and integration	75	4	1-5	4-20	12.84	3.83
WHOQOL-SRPB spiritual strength	75	4	1-5	4-20	12.92	3.23
WHOQOL-SRPB inner peace	75	4	1-5	4-20	10.93	4.04
WHOQOL-SRPB hope and optimism	75	4	1-5	4-20	10.51	3.38
WHOQOL-SRPB faith	75	4	1-5	4-20	11.16	3.37

HAD: Hospital anxiety depression scale, EQ5D (VAS): Euro Quality of Life 5 Dimensions (visual analogue scale), FFMQ: Five Facets Mindfulness Questionnaire, CPAQ: Chronic Pain Acceptance Questionnaire, WHOQOL-SRPB: WHO Quality of Life –Spiritual, Religious and Personal Beliefs.

Table 5. *Correlations amongst questionnaires and pain measures*

	**HAD score**	**EQ5D index**	**EQ5D VAS**	**FFMQ score**	**CPAQ score**	**WHO score**	**Pain intensity**	**Pain duration**	**Pain spread**
**HAD score**									
**EQ5D index**	-.300								
**EQ5D VAS**	-.347*	.378*							
**FFMQ score**	-.470*	.019	.019						
**CPAQ score**	-.292	.212	.366*	-.005					
**WHO score**	-.347*	.189	.236	.485*	.064				
**Pain intensity**	.292	-.333*	-.270	-.230	-.160	-.235			
**Pain duration**	.011	.051	.004	.070	-.101	.166	-.108		
**Pain spread**	.298	-.104	-.158	.008	-.273	.090	.095	.330*	

Abbreviations: Hospital Anxiety and Depression scale (HAD), Euroqol 5 Dimensions (EQ5D), Visual Analogue Scale (VAS), Five Facets Mindfulness Questionnaire (FFMQ), Chronic Pain Acceptance Questionnaire (CPAQ), WHO Quality of Life Spiritual, Religious and Personal Beliefs (WHO). *Correlation is significant at the 0.005 level (2-tailed)

3.3. Cellular measurements

The mean TL of the group was 1.163 (n=84, SD=0.412) and the group mean TA (ln transformed) was 2.064 TPG units/µL (n=80, SD=1.774). At group level, all routine blood test results were

within normal reference intervals according to Swedish standards at Karolinska University Laboratory (data not shown).

3.4. Telomere measurement correlations and associations with PROMs

TL was negatively associated with age (r=-.210, p=.055) and in an age-adjusted correlation, only P-cholesterol (r=.225, p=.041) and LDL-cholesterol (r=.218, p=.050) correlated with TL. No correlations were found between measures in blood samples and the TA (data not shown). With self-report measures, a negative correlation was found between the TL and HAD score (r = -.219, p = .047). Also, a positive correlation was seen between the TL and WHOQOL-SRPB (r = .224, p = .052). Interestingly the TL was not correlated to any of the pain measures. The TA did not correlate with any of the self-reported questionnaires or pain measures. When using an analytic approach similar to Hassett et al [5], where the groups were divided into "high pain/low pain" and "high HAD score/low HAD score" (the group mean score was used as cut-off), and ANCOVA analyses adjusted for age were conducted with TL and TA as dependent variables, no associations were detected (for TA and HAD: $Eta^2 = 0.00$; p = 0.901, for TL and HAD: $Eta^2 = 0.012$; p = 0.32; for TA and pain: $Eta^2 = 0.011$, p = 0.357; for TL and pain: $Eta^2 = 0.001$; p = 0.78).

Table 6. *Partial correlations with p-values, between TA/TL and self-report measures*

	HAD	EQ5D index	EQ5D VAS	FFMQ	CPAQ	WHO	PI	PD	PS
TL	-.219 (.047)	-.003 (.977)	.141 (.211)	.131 (.250)	.189 (.089)	.224 (.052)	-.154 (.166)	.083 (.462)	-.188 (.090)
TA	-.042 (.709)	-.080 (.480)	-.100 (.383)	.039 (.738)	-.031 (.787)	.145 (.225)	-.177 (.119)	.181 (.113)	.222 (.049)

Abbreviations: Hospital Anxiety and Depression scale (HAD), Euroqol 5 Dimensions (EQ5D), Visual Analogue Scale (VAS), Five Facets Mindfulness Questionnaire (FFMQ), Chronic Pain Acceptance Questionnaire (CPAQ), WHO Quality of Life Spiritual, Religious and Personal Beliefs (WHO), Pain Intensity (PI), Pain Duration (PD), Pain Spread (PS), Telomere Length (TL), Telomerase Activity (TA).

3.5. Mediation effects

As the first order correlations and theoretical consideration suggested that the relationship between spiritual well-being (WHOQOL-SRPB) and TL might be mediated by the effect of the former on depression and anxiety (HAD scale) we inspected a potential mediation model in an exploratory fashion. First, the direct effects a, b and c were modeled in univariate regression models. Beta-weights, i.e. partial correlations coefficients are given for the different paths in Figure 1. Modelling the total effect using the mediation path, the interaction a*b (Total Effect = c + a*b), following the standard mediation model of Baron and Kenny [44] yields a total effect (beta = 0.008; p = .045), i.e. supports the idea of a mediation of the direct effect of spiritual well-being on TL by anxiety and depression (HAD scale). However, the indirect effect, the interaction a*b (ab = 0.002; beta = -.23; p = .15) is not significant.

Figure 1. Mediation model

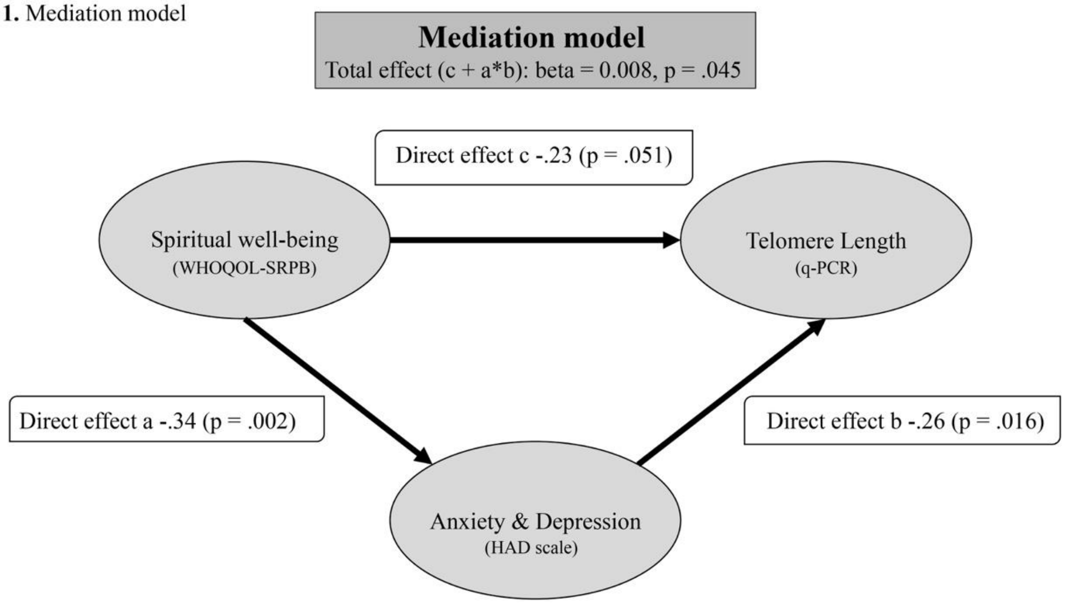

WHOQOL-SRPB: WHO Quality of Life –Spiritual, Religious and Personal Beliefs, HAD scale: Hospital Anxiety and Depression Scale; q-PCR: quantitative Polymerase Chain Reaction.

4. DISCUSSION

To the best of our knowledge this is the first scientific study that explores the body-mind-spirit perspective of pain in an integrated and comprehensive manner, by investigating both telomeres, blood tests and questionnaires covering pain, coping, depression, mindfulness and spiritual well-being, including personal and religious beliefs.

Our typical study participant was a woman that rated her overall health poorly, with EQ5D VAS mean being 41 out of a maximum of 100. In comparison, the Swedish population norm (not standardized) is 83 out of 100 [48]. The EQ5D index value is 0.85 for the Swedish general population, whereas our participant mean score was 0.26 (SD 0.32). Our average participant had a clinically probable risk for both anxiety and depression with a moderate pain coping capacity - more for pain willingness than for activity engagement. When aspects of mindfulness were

reported, our average participant was struggling with acting with awareness or staying focused on the task at hand without becoming distracted. Mindfulness meditation could be beneficial as an intervention since it can improve pain, depression symptoms and quality of life with no risk of addiction [49]. On average, participants scored moderately on all facets of the WHOQOL-SRPB. Their sense of meaning and purpose in life together with a feeling of wholeness and integration was slightly more prominent while there was a lack of hope and optimism as well as few experiences of awe and wonder. Despite the obvious challenges to realize a universal instrument for spirituality that allow equitable comparison among people with different worldviews [50] the SRPB dimensions used in our study can be seen as related to spiritual quality of life in one way or another. Since spirituality seems to enhance quality of life and affective well-being in chronic pain patients, partly mediated through coping [51-53], health care interventions including spiritual health and respecting religious and personal meaning dimensions might be beneficial.

Interestingly, the blood samples did not deviate from normal reference intervals (data not shown) indicating that patients with severe and long-lasting pain paradoxically appear "perfectly healthy". However, more complex proteomics or pro-inflammatory markers have been shown to deviate in chronic pain patients and correlate with other health aspects such as HAD scores [54].

Important to mention is the unbalanced gender ratio (86-14) in this sample, characteristic of the pain patient population nationally as well as globally, illuminating a gender issue. Women are noticeably overrepresented amongst patients living with chronic pain [55-57] and it has been highlighted as a gendered condition possibly subjected to medical gender bias [56]. Further adding to this, a report from 2018 requested by the Swedish Government concluded that there were close to zero (1 of 185) reviews with focus on women in this female dominated condition [57].

Regarding correlations, the participants' self-reported anxiety and depression had stronger relationships with measures of mindfulness and spiritual well-being than with pain measures: Indeed, our findings indicate that stronger personal, spiritual, or religious beliefs may be protective of depression and anxiety, and that this may perhaps be mediated by increased mindfulness - presence in everyday life. However, our study design does not allow us to assess the direction of association, whereby it may also be so that decreased depression and anxiety instead enables increased mindfulness and personal, spiritual and religious beliefs.

One interesting finding in this study is that the WHOQOL-SRPB had a positive correlation to TL, meaning that a greater sense of spiritual well-being, meaning, purpose and wholeness might be linked with longer telomeres. Furthermore, our exploratory model supports the idea of a mediation of the direct effect of spiritual well-being on TL by anxiety and depression as measured by the HAD scale. The relationship between spiritual well-being and TL could indicate a weak relationship between PROM's and aspects of telomeres. As we had only a small sample, the correlations, though interesting from a conceptual point of view, did not reach conventional significance thresholds. This likely indicates a power problem which underlines the need for replication with a larger data set. The HAD scale was negatively correlated with the TL, meaning that higher depression/anxiety scores, could be linked to shorter telomeres. Similar findings have been described by Hassett et al [5] and Sibille et al [26].

Another finding is the absence of correlation between TL and the pain measures selected in this study. This is surprising since others have found statistically significant correlations between TL and pain measures [5, 21-24]. In contrast, TA had a positive relationship with pain spread, i.e. high TA was associated with higher pain spread. But again, this correlation is not large enough to

reach a convincing significance level with the sample size we had available. This is ambiguous since TL is negatively associated with pain spread to a notable degree. Surprisingly, when repeating the cross table statistical analysis of Hassett et al [5] by grouping high pain-high depression in contrast to low pain-low depression we were still unable to reproduce the results with correlations between TL and high depression - high pain (data not shown). For all lab tests, pain measures and other self-report measures it is the HAD scale and the WHOQOL-SRPB that emerge in correlation with TL.

In summary, what ultimately correlates with the genomic integrity within this sample of chronic pain patients is neither quantitative pain measures or scales, nor index numbers generated for health economy calculation purposes, but instead anxiety, depression and personal, spiritual and religious beliefs. And those, in turn, have a significant relation to both EQ5D VAS and some of the self-report pain measures. While how patients feel is often dismissed in health care [8, 9], it seems to be mirrored by the state of genomic integrity as well as in self-reports in our study. This is in line with the model proposed by Wideman et al [10], with patients as experts on their own condition.

Although the recommendations by large organizations like IMMPACT is to use NRS as the core measurement for clinical pain [29], it doesn't relate to the genomic integrity of the participants in this study. And while measures of emotional functioning are included in the recommendations for core outcome measures, the spiritual dimension of pain is absent from the international recommendations [29].

The global opioid crisis is a painful reality that is of foremost relevance to pain management. Opioids are commonly prescribed to chronic pain patients in spite of doubtful effectiveness as long-term treatment. Between 1999-2014 approximately 165 000 persons died of

overdose related to opioid medications - only in the United States [58]. Moreover, during 2015 opioids were responsible for a third of the almost half a million global drug deaths [59]. And despite the relation to global morbidity, mortality and doubtful long-term effectiveness, 24 % of the participants in our sample of chronic pain patients had opioids prescribed for their pain. Our study gives reasons to suggest that it might be beneficial for health care to be guided by interventions also including the emotional and spiritual dimensions of pain, as well as personal and religious meaning.

Increasingly, more patients are offered rehabilitation in so-called multimodal programs with psychological and physical activities and this biopsychosocial approach and model in pain management is currently recommended in Sweden [57, 60] , though without reference to spiritual aspects of health and pain. Given that TL is a good indicator for longevity and robustness of health [12] and that spiritual well-being is somewhat associated with TL this indicates a novel arena with potential in pain rehabilitation. Our findings are preliminary and warrant further scrutiny.

4.1. Methodological considerations and limitations

A limitation with this study is its cross-sectional design with the innate inability to draw causal conclusions, and its comparatively small sample size. The latter was due to organisational restrictions that could not be remedied. However, the goal was rather to generate some preliminary insights, questions and hypotheses for future studies. Another apparent limitation is the lack of a control group. This is part of the nature of this study as patients cannot be randomised to become chronic pain patients. Only naturalistic cohorts of healthy, age matched controls would be possible, and we were unable to recruit such controls for this study for organisational reasons.

There are several biases in this project. Firstly, the two sites had different acceptance rates which can generate bias. Together, 45% of all eligible persons accepted participation which increases the risk of bias and raises the concern of the sample not being representative of the target population. Yet, by comparing our data to national health registry data [47] we can see that our sample was in fact representative of the target population, as well as assessing on what accounts they were not.

The double data entry and cross-checking and careful validation of the database before analysis improved methodological strength in this study. Also, the inclusion of two geographically separate locations as study sites with the aim of improving generalizability can be seen as a strength. In addition, since Complementary and Alternative Medicine (CAM) modalities are widely used by pain patients [61], the inclusion of The Vidar Clinic as a formal health care provider including certain CAM therapies can also been seen as a strength contributing to generalizability.

Another limitation is that other potential confounders apart from BMI, age or sex may have accounted for some of the results. We did not investigate the effect of tobacco or alcohol use, which are known to confound telomere measurements. Also, the initial preparation of the blood samples used for TL and TA measurement were done by different staff at the two sites. However, the standard protocol was followed and when conducting analyses of the mononuclear cells in laboratory environment, the quality of blood sampling and initial preparation could be validated. Additionally, the randomisation of the telomere samples as well as blinding of laboratory personnel performing the tests added to methodological strengths and reduced bias.

The size of the correlations we found were rather small, between r = .2 and .3. Because of the small sample size and the comparatively large number of correlations, inherent in an exploratory project such as this one, we would not want to draw any definitive conclusions. But it

seems to be an interesting hypothesis to follow up in a subsequent study whether the positive association between TL and spiritual well being can be substantiated, as well as the negative association between depression and anxiety and TL. Also the positive correlation between TA and pain spread warrants further scrutiny. The former would be indicative of a protective effect of spirituality and meaning-making against physical disintegration. The latter would point to an association between repair processes and the spreading of pain. With such focussed hypotheses further studies could produce more confirmatory evidence than our data can provide.

Also, it should be noted that our mediation model is intuitive, but far from definitive. That spiritual well being together with an interaction between SRPB and HAD should have a positive, albeit small, influence on telomere length certainly warrants further scrutiny. As our data were cross-sectional, it would be mandatory to have longitudinal data which would lend itself to a clear causal analysis, which our analysis cannot provide. Furthermore, one can argue that mediation is only demonstrated according to the original Baron and Kenny approach, but not according to more recent discussions, which would require an independent interaction term to be significant. However, we nevertheless think that this potential mediation might be interesting for other researchers to scrutinize in more detail.

5. CONCLUSION

In summary, chronic pain patients participating in this study confirm the view of a complex and holistic web of health dimensions that are affected by and associated with their suffering and a heavy burden of symptoms. Our findings show that telomeres tend to be shorter in more anxious and depressed pain patients, but not in patients with more self-reported pain. Intriguingly, spiritual well-being as measured with WHOQOL-SRPB, was in this sample of patients correlated with TL.

TA was more pronounced in patients reporting higher pain spread. These findings are preliminary and need confirmation. This study confirms the notion that numerical estimations of pain may be too crude to capture the complexity of chronic pain, and the widespread use of such measures in health care and research can possibly be explained by an historical tendency to assess the effectiveness of pharmacological pain management. Considering the international opioid crisis, non-pharmacological and non-opioid treatments emerge as a way forward. We have indicated some reason to believe that the emotional and spiritual dimensions of pain and health is a neglected but still highly important aspect for the well-being and longevity of chronic pain patients and hence should also be a focus in pain management and most importantly for further research.

Authors contributions

Linn Rönne-Petersén: Investigation, Validation, Visualization, Data Curation, Writing – original draft, Project administration. **Maria Niemi**: Methodology, Data Curation, Formal analysis, Writing – Review and Editing. **Harald Walach**: Formal analysis, Validation, Writing – Review and Editing. **Catharina Lavebratt**: Investigation, Resources, Methodology, Writing – Review and Editing. **Liu L.,Yang**: Investigation, Writing – Review and Editing. **Björn Gerdle**: Conceptualization, Methodology, Resources, Writing – Review and Editing, Funding acquisition **Bijar Ghafouri**: Investigation, Writing – Review and Editing. **Torkel Falkenberg**: Conceptualization, Supervision, Methodology, Resources, Writing – Review and Editing, Visualization, Project administration, Data Curation, Funding acquisition.

Financial support

This work was supported by Ekhagastiftelsen, KI Research School in Health Care Sciences, Gyllenbergsstiftelsen, Integrative Care Science Center, Vidarstiftelsen and Mahle stiftung; making the research possible without any demands of retribution.

Declaration of Competing Interest

Declarations of interest: none

Acknowledgements

The many anonymous patients are deeply appreciated for generously giving of body, mind and spirit, making this project possible. Also, the following persons are gratefully acknowledged: Annele Claeson for her general support and efforts during data entry, Eva-Britt Lind and Ulrika Wentzel Olausson for essential contribution in recruitment, data collection, and data entry. Vincent Millisher for substantial contribution in analysing telomere data, as well as editing and reviewing. Tobias Sundberg for contribution to database development and initial crude analysis. Sebastian Sauer for help with evaluating the mediation model. Maria Arman for insights and expertise on nursing and Johanna Hök Nordberg for integrated feedback on manuscript and both for thoughtful supervision. Linköping Smärtcentrum and The Vidar Clinic are acknowledged for smooth collaboration. We are also grateful for initial advice and guidance on genomic aspects of the project by Elizabeth Blackburn. Lastly a thank you to the unnamed reviewers who helped elevate this work.

Data availability statement

Data can be obtained from the authors on request

REFERENCES

[1] R.M. Leadley, N. Armstrong, Y.C. Lee, A. Allen, J. Kleijnen, Chronic diseases in the European Union: the prevalence and health cost implications of chronic pain. J Pain Palliat Care Pharmacother. 26(4) (2012) 310-25. doi: 10.3109/15360288.2012.736933.

[2] S.P Bergman, P. Herrstrom, K. Hogstrom, I.F Peterson, B. Svensson, L.T. Jacobsson, Chronic musculoskeletal pain, prevalence rates, and sociodemographic associations in a Swedish population study, J. Rheumatol, 28(6) (2001) 1369-77.

[3] J. McBeth, D.P. Symmons, A.J. Silman, T. Allison, R. Webb, T. Brammah, G.J. Macfarlane, Musculoskeletal pain is associated with a long-term increased risk of cancer and cardiovascular-related mortality. Rheumatology (Oxford), 48(1) (2009) 74-7. doi: 10.1093/rheumatology/ken424.

[4] F. Wolfe, A.L. Hassett, B. Walitt, K. Michaud, Mortality in fibromyalgia: a study of 8,186 patients over thirty-five years. Arthritis Care Res (Hoboken). 63(1) (2011) 94-101. doi: 10.1002/acr.20301

[5] A.L. Hassett, , E. Epel, D.J. Clauw, R.E. Haris, S.E.Harte, A. Kairys, S. Buyske, D.A. Williams. Pain is associated with short leukocyte telomere length in women with fibromyalgia. J Pain. **13**(10) (2012) 959-69. doi: 10.1016/j.jpain.2012.07.003.

[6] B. Gerdle, J. Bjork, C. Henriksson, A. Bengtsson, Prevalence of current and chronic pain and their influences upon work and healthcare-seeking: a population study. J Rheumatol. 31(7) (2004) 1399-406.

[7] K.J. Reid, J. Harker, M.M. Bala, C. Truyers, E. Kellen, G.E. Bekkering, J. Kleijnen, Epidemiology of chronic non-cancer pain in Europe: narrative review of prevalence, pain treatments and pain impact. Current Medical Research and Opinion. **27**(2) (2011) 449-462. doi: 10.1185/03007995.2010.545813. Epub 2011 Jan 3.

[8] L. De Ruddere, and K.D. Craig, Understanding stigma and chronic pain: a-state-of-the-art review. Pain. **157**(8) (2016) 1607-10. doi: 10.1097/j.pain.0000000000000512.

[9] F. Toye, K. Seers, N. Allcock, M. Briggs, E. CArr, J. Andrews, K. Barker, Patients' experiences of chronic non-malignant musculoskeletal pain: a qualitative systematic review. Br J Gen Pract. **63**(617) (2013) 829-41.

[10] T.H. Wideman, R.R. Edwards, D.M. Walton, M.O. Martel, A. Hudon, D.A. Seminowicz The Multimodal Assessment Model of Pain: A Novel Framework for Further Integrating the Subjective Pain Experience Within Research and Practice. Clin J Pain. 35(3) (2019) 212-221. doi: 10.1097/AJP.0000000000000670.

[11] E. Blackburn, E. Epel, and J. Lin, Human telomere biology: A contributory and interactive factor in aging, disease risks, and protection. Science. 350(6265) (2015) 1193-8. doi: 10.1126/science.aab3389.

[12] E.S. Epel, E.H. Blackburn, J. Lin, F.S. Dhabhar, N.E. Adler, J.D. Morrow, R.M. Cawthon, Accelerated telomere shortening in response to life stress. Proc Natl Acad Sci U S A. 101(49) (2004) 17312-5. doi: 10.1073/pnas.0407162101. Epub 2004 Dec 1.

[13] E.S. Epel, J. Lin, F.H. Wilhelm, O.M. Wolkowitz, R. Cawthon, N.E. Adler, C. Dolbier, W.B. Mendes, E.H. Blackburn. Cell aging in relation to stress arousal and cardiovascular

disease risk factors. Psychoneuroendocrinology. 31(3) (2006) 277-87. doi: 10.1016/j.psyneuen.2005.08.011.
[14] P.C. Haycock, E.E. Heydon, S. Kaptoge, A.S. Butterworth, A. Thompson, P. Willeit, Leucocyte telomere length and risk of cardiovascular disease: systematic review and meta-analysis. Bmj. 349 (2014) 4227. doi: https://doi.org/10.1136/bmj.g4227
[15] K.T. Sibille, L. Witek-Janusek, H.L. Mathews, R.B. Fillingim, Telomeres and epigenetics: potential relevance to chronic pain. Pain. 153(9) (2012) 1789-93. doi: 10.1016/j.pain.2012.06.003.
[16] E. Blackburn, and E. Epel, The telomere effect : a revolutionary approach to living younger, healthier, longer. Orion spring, London, 2017.
[17] A.Vaiserman, and D. Krasnienkov, Telomere Length as a Marker of Biological Age: State-of-the-Art, Open Issues, and Future Perspectives. Front. Genet. 11:630186 (2021) doi: 10.3389/fgene.2020.630186.
[18] E.S. Epel, J. Lin, F.S. Dhabhar, O.M. Wolkowitz, E. Puterman, L. Karan, E.H. Blackburn, Dynamics of telomerase activity in response to acute psychological stress. Brain Behav Immun, 24(4) 2010 531-9. doi: 10.1016/j.bbi.2009.11.018.
[19] D.A. Skvortsov, M.E. Zvereva, O.V. Shpanchenko, O.A. Dontsova, Assays for detection of telomerase activity. Acta Naturae. 3(1) (2011) 48-68.
[20] J. Daubenmier, J. Lin, E. Blackburn, F.M. Hecht, J. Kristeller, N. Maninger, M. Kuwata, P. Bacchetti, P.J. Havel, E. Epel, Changes in stress, eating, and metabolic factors are related to changes in telomerase activity in a randomized mindfulness intervention pilot study. Psychoneuroendocrinology. 37(7) (2012) 917-28.
[21] C.L. Le Maitre, A.J. Freemont, and J.A. Hoyland, Accelerated cellular senescence in degenerate intervertebral discs: a possible role in the pathogenesis of intervertebral disc degeneration. Arthritis Res Ther. 9(3) (2007) R45. doi: 10.1186/ar2198.
[22] S. Dechsupa, W. Yingsakmongkol, W. Limthongkul, W. Singhatanadgige, S. Honsawek, Relative telomere length and oxidative DNA damage in hypertrophic ligamentum flavum of lumbar spinal stenosis. PeerJ. 6: (2018) e5381. doi: 10.7717/peerj.5381
[23] T. Maeda, Y. Kudo, T. Horiuchi, N. Makino, Clinical and anti-aging effect of mud-bathing therapy for patients with fibromyalgia. Mol Cell Biochem. 444(1-2) (2018) 87-92. doi: 10.1007/s11010-017-3233-4.
[24] K.T. Sibille, H. Chen, E.J. Bartley, J. Riley, T.L. Glover, C.D. King, H. Zhang, Y. Cruz-Almeida, B.R. Goodin, A. Sotolongo, M.E. Petrov, M. Herbert, H.W. Bulls, J.C. Edberg, R. Staud, D. Redden, L.A Bradley, R.B. Fillingim, Accelerated aging in adults with knee osteoarthritis pain: consideration for frequency, intensity, time, and total pain sites. Pain Rep. 2(3) (2017) e591. doi: 10.1097/PR9.0000000000000591.
[25] A.M. Steward, J.D. Morgan, J.P. Espinosa, D.C. Turk, K.V. Patel, Chronic Pain and Telomere Length in Community-Dwelling Adults: Findings From the 1999 to 2002 National Health and Nutrition Examination Survey. J Pain. 18(12) (2017) 1517-1525. doi: 10.1016/j.jpain.2017.08.006.
[26] K.T. Sibille, T. Langaee, B. Burkley, Y. Gong, T.L. Glover, C. King, J.L. Riley, C. Leeuwenburgh, R. Staud, L.A. Bradley, R.B. Fillingim, Chronic pain, perceived stress, and cellular aging: an exploratory study. Mol Pain. 8 (2012) 12. doi: 10.1186/1744-8069-8-12.
[27] G, Lucchetti, *Spirituality, Religiousness and Health [Elektronisk resurs]*. 2019: Springer International Publishing.

[28] G. Lucchetti, H.G. Koenig, A.L.G. Lucchetti, Spirituality, religiousness, and mental health: A review of the current scientific evidence. World J Clin Cases. 16;9(26) (2021) 7620-7631. doi: 10.12998/wjcc.v9.i26.7620.

[29] R.H. Dworkin, D.C. Turk, J.T. Farrar, J.A. Haythornthwaite, M.P. Jensen, N.P. Katz, R.D. Kerns, G.Stucki, R.R. Allen, N. Bellamy, D.B. Carr, J. Chandler, P. Cowan, R. Dionne, B.S. Galer, S. Hertz, A.R. Jadad, L.D. Kramer, D.C. Manning, S. Martin, C.G. McCormick, M.P McDermot, P. McGrath, S. Quessy, B.A. Rappaport, W. Robbins, J.P. Robinson, M. Rothman, M.A. Royal, L. Simon, J.W. Stauffer, W. Stein, J. Tollett, J. Wernicke, J. Witter, Core outcome measures for chronic pain clinical trials: IMMPACT recommendations. Pain. 113(1-2) (2005) 9-19. doi: 10.1016/j.pain.2004.09.012.

[30] L.M. McCracken, K.E. Vowles, and C. Eccleston, Acceptance of chronic pain: component analysis and a revised assessment method. Pain. 107(1-2) (2004)159-66. doi: 10.1016/j.pain.2003.10.012.

[31] G.S. Rovner, K. Arestedt, B. Gerdle, B. Börsbo, L.M. McCracken, Psychometric properties of the 8-item Chronic Pain Acceptance Questionnaire *(CPAQ-8) in a Swedish chronic pain cohort.* J Rehabil Med. 46(1) (2014) 73-80. doi: 10.2340/16501977-1227.

[32] S.J. Olsson, O. Ekblom, E. Andersson, M. Börjesson, L.V. Kallings, Categorical answer modes provide superior validity to open answers when asking for level of physical activity: A cross-sectional study. Scand J Public Health. 44(1) (2016) 70-6. doi: 10.1177/1403494815602830.

[33] EuroQol-Group, EuroQol--a new facility for the measurement of health-related quality of life. Health Policy. 16(3) (1990) 199-208. doi: 10.1016/0168-8510(90)90421-9.

[34] I. Bjelland, A.A. Dahl, T.T. Haug, D. Neckelman, The validity of the Hospital Anxiety and Depression Scale. An updated literature review. J Psychosom Res. 52(2) (2002) 69-77. doi: 10.1016/s0022-3999(01)00296-3.

[35] J. Lisspers, A. Nygren, and E. Söderman, Hospital Anxiety and Depression Scale (HAD): some psychometric data for a Swedish sample. 96(4) (1997) 281-286. doi: 10.1111/j.1600-0447.1997.tb10164.x.

[36] R. LoMartire, B.O. Äng, B. Gerdle, L. Vixner, Psychometric properties of Short Form-36 Health Survey, EuroQol 5-dimensions, and Hospital Anxiety and Depression Scale in patients with chronic pain. Pain. 161(1) (2020) 83-95. doi: 10.1097/j.pain.0000000000001700.

[37] S.M. Skevington, K.S. Gunson, and K.A. O'Connell, Introducing the WHOQOL-SRPB BREF: developing a short-form instrument for assessing spiritual, religious and personal beliefs within quality of life. Qual Life Res. 22(5) (2013) 1073-83. doi: 10.1007/s11136-012-0237-0.

[38] C. Melder, Vilsenhetens epidemiologi -en religionspsykologisk studie i existentiell folkhälsa [The epidemiology of lost meaning − A study in psychology of religion and existential public health in a Swedish context], in Acta Universitatis Upsaliensis, Psychologia et Sociologia Religionum 25th [Doctoral dissertation] 2011, Uppsala University Uppsala, Sweden. p. 305.

[39] R.A. Baer, G.T. Smith, J. Hopkins, J. Krietemeyer, L. Toney, Using self-report assessment methods to explore facets of mindfulness. Assessment. 13(1) (2006) 27-45. doi: 10.1177/1073191105283504.

[40] J.L. Lilja, A. Frodi-Lundgren, J.J. Hanse, T. Josefsson, L.G. Lundh, C. Sköld, E. Hansen, A.G. Broberg, Five Facets Mindfulness Questionnaire--reliability and factor structure: a Swedish version. Cogn Behav Ther. 40(4) (2011) 291-303.

[41] M. Hou, D. Xu, M. Bjorkholm, A. Gruber, Real-time quantitative telomeric repeat amplification protocol assay for the detection of telomerase activity. Clin Chem. 47(3) (2001) 519-24.

[42] N.J. O'Callaghan, and M. Fenech, A quantitative PCR method for measuring absolute telomere length. Biol Proced Online. 13 (2011) 3. doi: 10.1186/1480-9222-13-3.

[43] R.M. Cawthon, Telomere measurement by quantitative PCR. Nucleic Acids Res. **30**(10) (2002) e47. doi: 10.1093/nar/30.10.e47.

[44] R.M. Baron, and D.A. Kenny, The moderator-mediator variable distinction in social psychological research: conceptual, strategic, and statistical considerations. J Pers Soc Psychol. **51**(6) (1986)1173-82. doi: 10.1037//0022-3514.51.6.1173.

[45] D.J. Benjamin, J.O. Berger, M. Johannesson, B.A. Nosek, E.J. Wagenmakers, R. Berk, K.A. Bollen, B. Brembs, L. Brown, C. Camerer, D. Cesarini, C.D. Chambers, M. Clyde, T.D. Cook, P. De Boeck, Z. Dienes, A. Dreber, K. Easwaran, C. Efferson, E. Fehr, F. Fidler, A.P. Field, M. Forster, E.I. George, R. Gonzalez, S. Goodman, E. Green, D.P. Green, A.G. Greenwald, J.D. Hadfield, L.V. Hedges, L. Held, T. Hua Ho, H. Hoijtink, D.J. Hruschka, K. Imai, G. Imbens, J.P.A. Ioannidis, M. Jeon, J.H. Jones, M. Kirchler, D. Laibson, J. List, R. Little, A. Lupia, E. Machery, S.E. Maxwell, M. McCarthy, D.A. Moore, S.L. Morgan, M. Munafó, S. Nakagawa, B. Nyhan, T.H. Parker, L. Pericchi, M. Perugini, J. Rouder, J. Rousseau, V. Savalei, F.D. Schönbrodt, T. Sellke, B. Sinclair, D. Tingley, T. Van Zandt, S. Vazire, D. J. Watts, C. Winship, R.L. Wolpert, Y. Xie, C. Young, J. Zinman, V.E. Johnson. Redefine statistical significance. Nat Hum Behav. 2(1) (2018) 6-10. doi: 10.1038/s41562-017-0189-z.

[46] G. Gigerenzer. Mindless statistics. Journal of Socio-Economics, 33 (2004) 587-606. https://doi.org/10.1016/j.socec.2004.09.033

[47] SQRP, *Bilaga rapport 2020:1 Specialistvård: patienter som avslutats under 2019*. 2020.

[48] A. Szende, Self-Reported Population Health: An International Perspective based on EQ-5D [Elektronisk resurs]. 2014.

[49] L. Hilton, S. Hempel, B.A. Ewing, E. Apaydin, L. Xenakis, S. Newberry, B. Colaiaco, A. Rualez Maher, R.M. Shanman, M.E. Sorbero, M.A. Maglione, Mindfulness Meditation for Chronic Pain: Systematic Review and Meta-analysis. Ann Behav Med. **51**(2) (2017)199-213. doi: 10.1007/s12160-016-9844-2.

[50] J.H. Hammer, N.G. Wade, and R.T. Cragun, Valid assessment of spiritual quality of life with the WHOQOL-SRPB BREF across religious, spiritual, and secular persons: A psychometric study. Psychology of Religion and Spirituality, 12(4), 440–450. https://doi.org/10.1037/rel000026650.

[51] E. Biccheri, N. Roussiau, C. Mambet-Doue, Fibromyalgia, Spirituality, Coping and Quality of Life. J Relig Health 55 (4) (2016), 1189-97. doi: 10.1007/s10943-016-0216-9.

[52] O. Dedeli. and G. Kaptan, Spirituality and Religion in Pain and Pain Management. Health Psychol Res. **1**(3) (2013) e29. doi: 10.4081/hpr.2013.e29

[53] A. Moreira-Almeida. and H.G. Koenig, Religiousness and spirituality in fibromyalgia and chronic pain patients. Curr Pain Headache Rep. **12**(5) (2008) 327-32. doi: 10.1007/s11916-008-0055-9.

[54] B. Gerdle, E. Backryd, T. Falkenberg, E. Lundström, B. Ghafouri, Changes in inflammatory plasma proteins from patients with chronic pain associated with treatment in an interdisciplinary multimodal rehabilitation program - an explorative multivariate pilot study. Scand J Pain. **20**(1) (2019) 125-138. doi: 10.1515/sjpain-2019-0088.
[55] C. Ahlgren, A. Fjellman-Wiklund, K. Hamberg, E.E. Johansson, B.M. Stålnacke, The meanings given to gender in studies on multimodal rehabilitation for patients with chronic musculoskeletal pain – a literature review. Disability and Rehabilitation. **38**(23) (2016) 2255-2270. doi: 10.3109/09638288.2015.1127435.
[56] G. Smirthwaite, Tengelin, E., Borrman, T. (O)jämställdhet i hälsa och vård. (rev uppl) Stockholm: Sveriges kommuner och regioner; 2014.
[57] Statens beredning för medicinsk och social utvärdering. Behandling av långvariga smärttillstånd med fokus på kvinnor. En kartläggning av systematiska översikter. Vol. SBU-rapport nr 301. 2019: Stockholm: Statens beredning för medicinsk utvärdering (SBU), 2019.
[58] Dowell, D., T.M. Haegerich, and R. Chou, *CDC Guideline for Prescribing Opioids for Chronic Pain—United States, 2016.* JAMA, 2016. **315**(15): p. 1624-1645.
[59] UN, *World drug report 2017*. 2017, Vienna: United Nations, Office on Drugs and Crime.
[60] Statens beredning för medicinsk och social utvärdering, *Rehabilitering vid långvarig smärta: en systematisk litteraturöversikt.* Stockholm: Statens beredning för medicinsk och social utvärdering (SBU), 2010.
[61] Rosenberg, E.I., I. Genao, I. Chen, et al., *Complementary and alternative medicine use by primary care patients with chronic pain.* Pain Med, 2008. **9**(8): p. 1065-72.

[62] K. Preacher, & A. Hayes. SPSS and SAS procedures for estimating indirect effects in simple mediation models. Behavior Research Methods, Instruments, & Computers in Human Behavior. 36, (2004) 717-731.

[63] A.F. Hayes, Beyond Baron and Kenny: Statistical mediation analysis in the new millennium. *Communication Monographs. 76*(4) (2009) 408–420. https://doi.org/10.1080/03637750903310360

Manuscript
Click here to access/download;Manuscript;Finalised revised manuscript.docx

Title Page

Exploring emotional well-being, spiritual, religious and personal beliefs and telomere length in chronic pain patients - A pilot study with cross-sectional design

Linn Rönne-Petersén[1], Maria Niemi[2], Harald Walach[3], Catharina Lavebratt[4], Liu L. Yang[4], Björn Gerdle[5], Bijar Ghafouri[5] , Torkel Falkenberg[1]*

[1]Deparment of Neurobiology, Care sciences and Society, Division of Nursing 2, Karolinska Institutet, Stockholm, Sweden.

[2]Department of Global Public Health, K9 FHE Dalman, Karolinska Institutet, Stockholm, Sweden

[3]Change Health Science Institute, Berlin, Germany

[4]Department of Molecular Medicine and Surgery, Karolinska Institutet and Center for Molecular Medicine, Stockholm, Sweden

[5]Pain and Rehabilitation Centre, and Department of Health, Medicine and Caring Sciences, Linköping University, Linköping, Sweden

*Corresponding author
E-mail: torkel.falkenberg@ki.se

Abstract

Living with chronic pain is associated with substantial suffering and high societal costs. Patient reported outcomes (PROM's) and effects on cellular ageing should be considered in pain management. The aim of this study was to explore correlations of PROM's and cellular ageing (telomere length [TL] and telomerase activity [TA] measures) amongst patients with chronic non-malignant pain. This was an explorative pilot study with cross-sectional design and recruitment was done at two pain rehabilitation facilities in Sweden, with inpatient setting/integrative care and outpatient setting/multimodal care, respectively. Eighty-four patients were enrolled by referral to pain rehabilitation in Sweden. Main outcome measures collected after admission in addition to TL and TA were the following PROMs: Numerical Rating Scale, Chronic Pain Acceptance Questionnaire, Hospital Anxiety and Depression scale, Five Facets Mindfulness Questionnaire, WHO Quality of Life - Spiritual, Religious and Personal Beliefs and EuroQol 5 Dimensions. All the PROM's showed evidence of poor health status. TL correlated negatively with Hospital Anxiety and Depression ($r = -.219$, $p = .047$) and positively with WHO Quality of Life -Spiritual, Religious and Personal Beliefs ($r = .224$, $p = .052$). TL did not correlate with any of the pain measures. TA correlated positively with pain spread ($r = .222$, $p = .049$). A mediation of the direct effect of spiritual well-being on TL by anxiety and depression could be shown ($b = 0.008$; $p = .045$). The correlations between TL and spiritual well-being and anxiety and depression suggest some importance of emotional and spiritual dimensions in pain management, with implications for cellular aging, which needs further confirmation.

ClinicalTrials.gov Identifier: NCT02459639

KEYWORDS

Chronic pain, Emotional health, Spirituality, Telomere length, Telomerase activity, Integrative Health Care

Introduction

Chronic pain as a personal and societal condition

Chronic pain constitutes considerable personal suffering for patients living with it, and poses a great challenge for the healthcare system with high economic impact on society [1, 2]. Chronic pain has been described as an enigmatic phenomenon associated with substantial negative psychosocial factors and physical ill-health [3-5]. With 19-50 % of the European population affected, chronic pain is common, and in the female population the prevalence is at least 10 % higher [2, 6, 7]. Patients' reality of complex health issues is often misunderstood and misinterpreted by the healthcare system, leading to feelings of stigmatization and forcing the patients to seek healthcare repeatedly [8, 9]. Leadely et al [1] conclude that chronic pain is neither sufficiently prioritized in policies nor in budgets. Why such a widespread and incapacitating condition isn't more prioritized seems at large unclear.

Measuring pain

Despite the complex nature of pain and the subsequent need for a wider approach to study it, most pain research has mainly focused on pain intensity and duration [10]. In contrast, Wideman et al [10] have proposed a comprehensive model for how to integrate the nature of subjectivity and objectivity in pain, while at the same time arguing that we can neither measure the largest part of pain, namely the *pain experience (inner experience),* since it is non-observable and deeply personal. However, what research and clinicians can quantify are *pain measures* (subjective self-reported or objective non-self-reported), preferably mindful of *what* is being measured, i.e. not pain itself, but a limited part of the pain phenomena.

Telomeres, cellular ageing and pain

Telomeres are specific, protective structures at the ends of our chromosomes that are crucial for preventing genetic information from being lost during cell division [11]. Shortened telomere length (TL) is considered a predictor for a number of age-related diseases and mortality [12-14], although it is also affected by factors *independent* of chronological age [14-16]. In addition, TL shortening tends to be more the result of cumulative life stress and age-related diseases [17]. The enzyme telomer*ase* functions as a buffer against the cellular aging in the nucleus, steadily re-building the telomeres by adding on base pairs [11, 16]. Changes in TL can take months or years to be detectable, while telomerase activity (TA) is more dynamic and linked to short-term changes. Changes in TA enzyme levels have implications for telomere length and protection of the chromosomes [18, 19], making it suitable for examining more immediate effects in time of for example certain lifestyle factors or health-promoting behaviors [20].

A handful articles address the association between chronic pain and cellular ageing, whereas reports on pain and measures of TA are lacking. The majority of studies find significant correlations between chronic pain or degenerative tissue (associated with pain) and decreased TL [5, 21-24]. There are also studies not showing significant correlations [25, 26]. Notably, one study observed changes in TL when looking at combinations of pain and high and low stress, arguing that a combined effect of the two may accelerate cellular ageing [26]. It has been estimated that chronic pain shortens life by 4-5 years (as measured in loss of base pairs) and combined with depression it adds up to 6 years [5]. Hence, research aiming to investigate correlations between emotional well-being, pain experiences and cellular aging are urgently needed and could contribute to new ways of developing and evaluating chronic pain interventions. Health care interventions which include elements of emotional well-being and spiritual health are important in nursing care

and are emerging as potential health services components [27] with hypothetical implications for stress, mental health and pain management [27, 28] and even cellular aging.

Given these challenges and opportunities, the aim of this study was to explore correlations of Patient Reported Outcome Measurements [PROM's] and markers of cellular ageing amongst patients with chronic non-malignant pain. We used PROM's of clinical pain, physical and emotional functioning, spiritual, religious and personal beliefs, mindfulness and quality of life. We used TL and TA as measures of cellular ageing and potential mediators between PROMS and cellular ageing were explored.

Methods

Participant recruitment

The participants in this study were individuals suffering from chronic pain conditions referred to tertiary specialist centers and enrolled at either The Vidar Clinic, Järna *or* Pain and Rehabilitation Center, University Hospital, Linköping between June 2015 and December 2017. The study was preregistered (ClinicalTrials.gov Identifier NCT02459639), and ethically approved by the Regional Ethics Committee in Stockholm (EPN 2014/953-31/1). After oral and written information, participants made an informed decision whether to participate or not with no implications for their care. Inclusion criteria were ICD-10 M79, *OR* chronic pain (>3 months) in neck/shoulders and/or lower back or generalized pain (e.g. fibromyalgia), ≥18 years of age, fluent in Swedish, from Stockholm, Sörmland or Östergötland county councils. Comorbidities were allowed except for psychotic illness, addiction, and cancer.

In all, 57 patients at The Vidar Clinic and 138 patients at Linköping were invited to the study, of which 51 respectively 35 patients showed interest to participate. Of these, 51 respectively 33 patients met the inclusion criteria and agreed to participate. The final analysed sample consisted of 84 participating patients. The size of the Vidar Clinic was smaller compared to the Rehabilitation Center, University hospital, Linköping and hence explains the differences in recruitment proportions.

Data collection

This was an explorative pilot study with cross-sectional design, looking into clinical characteristics and correlations in a sample of persons with chronic non-malignant pain. Measures were collected after admission. Because of its exploratory nature, no sample size was calculated but a minimum of 70 enrolled patients was strived for, which allows to document an effect size of $r = .3$ with 72% power and reaches a power of 80% with $r = .32$. These are the ranges of correlations that were deemed likely and clinically important. Patients were recruited from two geographic locations – one with an out-patient setting (Linköping) and one with an in-patient setting (The Vidar Clinic). The same standard protocols for blood sampling were used at both sites, with the patients in a fasting state and where samples were collected after admission. Questionnaires were distributed at the time of the blood sampling. Double data entry was practiced for quality reasons and the completed database was then validated by cross-checking 12% of all data, whereby 0.7% were found to be incorrect due to small systematic errors. Those errors were controlled and adjusted for in the whole dataset before analysing.

Measurements

The measurements of this study comprised of TA and TL and PROM's of clinical pain, physical and emotional functioning, spiritual, religious and personal beliefs, mindfulness and quality of life. In addition, demographics, anthropometrics, blood pressure and blood samples were collected. A BMI between 18.5-24.9 kg/m^2 would be considered normal, as was a blood pressure <140/90 mmHg. The participants' blood samples were taken and sent for standard clinical analysis at Clinical Chemistry Laboratory at Linköping University Hospital and at Karolinska University Laboratory at Södertälje Hospital. The blood count was measured as well as the properties of the erythrocytes, leucocytes, thrombocytes, standard lipid status (triglycerides and cholesterol with quotas) and inflammatory profile (sedimentation rate, C-reactive protein). Also, levels of haemoglobin, glucose, sodium, potassium and creatinine were measured. The IMMPACT recommendations for core outcome measurements in chronic pain clinical trials [29] were considered and applied heading-wise, but not strictly followed in regard to the scales used. Some questionnaries were replaced due to being validated in Sweden and others were added due to relevance for this study.

Clinical pain was assessed by means of intensity using the Numerical Rating Scale [NRS] (0 = no pain, 10 = worst possible pain) which is a commonly used instrument, included in the Swedish National Quality Registry for Pain Rehabilitation [SQRP] and recommended by IMMPACT [29], A score <4 translates to mild pain, 4-6 moderate and >7 severe pain. Pain duration (years, months and days) and pain spread was also assessed by means of well established self-report questionaire from SQRP. In addition, the Chronic Pain Acceptance Questionnaire [CPAQ] ($\alpha \geq .80$), with the subscales of *pain willingness* and *activity engagement* was used because of its emphasis on aspects of coping, which is a focus area within pain research [30, 31]. In accordance

with recommendations from Dworkin et al [29], patient usage of rescue analgesics (fast-acting medications) were registered. The *physical functioning* was evaluated with the Swedish National Board of Health and Welfare's indicator questions on physical activity [FYS], recently validated [32], as well as EUROQOL 5 Dimensions-3L [EQ5D]($\alpha > .85$)[33]. The latter is an established measure of quality of life, including aspects of physical functioning. The EQ5D index value is a single number calculated from the different health states obtained within the questionnaire. This number is commonly ranging from 0 to 1, although negative numbers are possible, and it reflects how good or bad the health state is in comparison with the preferences of a general population in a specific country. The related EQ5D VAS is a single question with the score range 0-100, going from worst possible to best possible health [33]. *Emotional functioning* was measured with the Hospital Anxiety and Depression Scale [HAD Scale] ($\alpha > .89$), a reliable and valid instrument with subscales measuring *depression* and *anxiety* [34], validated in Sweden [35] and where high values indicate psychological distress [36]. *Participant ratings of global improvement and satisfaction with treatment*, as well as *Symptoms and adverse events* were assessed qualitatively, and will be reported separately.

Spiritual, religious and personal beliefs, are increasingly recognized as a distinctive, important, and cross-cultural concept in quality of life assessments [37]. Hence, in addition to the IMMPACT recommendations we assessed spiritual, religious and personal beliefs using the WHO Quality of Life, Spiritual, Religious and Personal Beliefs (WHOQOL-SRPB) ($\alpha = .85$) questionnaire in its appropriate Swedish adaptation [38]. The questionnaire has the eight following subscales: *spiritual connection, meaning and purpose in life, experiences of awe and wonder, wholeness and integration, spiritual strength, inner peace, hope and optimism, and faith*. Mindfulness using Five Facets Mindfulness Questionnaire [FFMQ]) ($\alpha = .81$) was included to

complement the WHOQOL-SRPB to assess if improvements in health could be mediated by increased mindfulness. It has five subscales: *non-reactivity, observe, acting with awareness, describe* and *non-judging*. FFMQ is the most frequently used questionnaire for mindfulness [39] and has been validated in Sweden [40]. The information of number of items and min-max score for each questionnaire is found in Table 3.

Telomerase measurement

Blood samples used for this study were collected between 2015 and 2017 at the two sites. TA was detected by real-time telomeric repeat amplification protocol (RT-TRAP) [41], with some modifications. In brief, blood samples were drawn in line with Swedish nursing standards, with the participants in a fasting and rested state (minimum 20 minutes). Whole blood was obtained in 8 ml BD Vacutainer® CPT™ Mononuclear Cell Preparation Tube - Sodium Citrate (Becton Dickinson). Thereafter, lymphocytes and monocytes were separated within two hours of sampling, according to manufacturer's protocol. Approximately half of the pelleted cells were lysed by incubation with 120 µl CHAPS (Merck Millipore, including 0.15 units/µl RiboLock [LifeTechnologies, Thermo Fisher Scientific]) on wet ice for 30 min and three short vortexes. Following this initial preparation done locally at laboratories on respective site, the samples were labelled and frozen at -80°C, after which they were transported to Center for Molecular Medicine (CMM) at Karolinska Institutet for storage and further analysis.

Thawed lysate was centrifuged at 4°C at 12000xg for 20 min and the supernatant was transferred to an empty tube. The total protein concentration was measured in the supernatant by the DC Protein Assay (Bio-Rad) and all patients' cell lysates were diluted to equal concentrations in CHAPS (0.95 µg/µl), aliquoted and refrozen at -80°C. An aliquot per sample was thawed and

equal amounts of total protein (3.8 µg) from each sample was added to a reaction mix with a total volume of 50 µL containing 1 mM of each dNTP, 20 mM Tris-HCl (pH 8.3), 2.5 mM MgCl2, 63 mM KCl, 0.05% Tween 20, 1 mM EGTA, 8 µmol of each of the primers TS (5'-AATCCGTCGAGCAGAGTT-3') and ACX (5'- GCGCGG(CTTACC)3CTAACC-3'). HepG2 (hepatic cancer) and ARO (thyroid cancer) cell lines were used as telomerase-positive controls, whereas CHAPS buffer and heat-inactivated samples were used as negative controls. TSR8 is an oligonucleotide with a sequence identical to the TS primer extended with 8 telomeric repeats being $AG(GGTTAG)_7$. Serial dilutions of TSR8 control template were used to generate a standard curve to calculate TA. The serial dilutions were 0.2 amoles/µL, 0.04 amoles/µL, 0.008 amoles/µL, 0.0016 amoles/µL, 0.00032 amoles/µL, and 0.000064 amoles/µL corresponding to 200, 40, 8, 1.6, 0.32 and 0.064 TPG units/µL; TPG is the total product generated, corresponding to the number of TS primers (1 unit equals 10^{-3} amoles or 600 molecules) that are extended with at least 3 TTAGGG repeats by telomerase in the extract in a 30 min incubation at 30°C. The reaction mix was incubated at 30°C for 30 min followed by termination at 95°C for 5 min. Then 8 µL of the telomeric repeat products (i.e. corresponding to 8/50*3.8=0.608 ug total protein) were used for the RT-TRAP assay amplified by 8 µL Power SYBR Green in 384-well plates. The reaction was performed on QuantStudio 7 Flex (Applied Biosystems; Life Technologies; Thermo Fisher Scientific Inc.) with the following conditions: 95°C for 10 min, followed by 36 repeats of 95°C for 20 s, 52°C for 30 s and 72°C for 60 s. Samples, controls and standard curve dilutions were run in triplicate, standard curve and controls on all plates. All samples from each patient were run on the same randomized plate. Efficiency was 99-105%. The mean of the correlation coefficients of the standard curves was > 0.993. The mean coefficients of variation (CV) of intra-plate Ct values for the standard dilutions of the five plates was CV = 0.56%, and the mean inter-plate CV of three inter-plate controls was

CV = 1.6%. The detection success rate was 100%. For each supernatant, aliquots are still there with original protein concentration.

Telomere length measurement

The other half of the pelleted cells, collected and prepared at both sites, were also stored at -80°C until DNA extraction. Genomic DNA was extracted based on the kit [42], but the speed of centrifuge was changed to 6000g and avoid vortex by inverse, and incubate at 37°C for 3 h. DNeasy® Blood & Tissue Kit (Qiagene). DNA concentration was quantified with NanoDrop ND-1000 Spectrophotometer (Nano-Drop Technologies Inc., Wilmington, DE, USA). Relative TL was determined using real-time quantitative polymerase chain reaction (qPCR) according to Cawthon et al's protocol [43] where the relative telomere to single copy gene (T/S) ratios was determined using a standard curve. In brief, each DNA sample (10 ng) was assessed for the telomere (*Tel*) and the single-copy gene (hemoglobin-b, *HGB*) in triplicate within the same 384-well plate, amplified by using Power SYBR Green in 10 µl total reaction volume. The reaction was performed on QuantStudio 7 Flex (Applied Biosystems; Life Technologies, Carlsbad, CA, USA) with the following conditions: 50°C for 2 min, then 95°C for 10 min, followed by 40 repeats of 95°C for 15 s and 60°C for 1 min, followed by a dissociation stage to monitor amplification specificity. The same standard curve of pooled DNA from these patient samples ranging from 80 ng to 0.128 ng, was run on each plate for both genes and was used to determine the quantity of each gene for each sample. This allowed controlling for differences in the efficiencies between that of *Tel* and *HGB*. The gene quantities were then used to determine the T/S ratio for each sample. DNA samples with a Ct standard deviation of ≥ 0.35 between triplicates or a Ct value outside the standard curve were omitted from the analyses. The correlation coefficients of the standard curves were above 0.99 for

each primer set and 384-plate. The inter-plate coefficient of variation (CV) of T/S ratio was 9.1% calculated from a patient sample run in seven 384-well plates. The TL analysis detection success rate was 100%. The primer sequences were (written 5'à3'): Tel1: CGGTTTGTTTGGGTTTGGGTTTGGGTTTGGGTTTGGGTT; Tel2: GGCTTGCCTTACCCTTACCCTTACCCTTACCCTTACCCT; *HGB* Fw: GCTTCTGACACAACTGTGTTCACTAGC; *HGB* Rv: CACCAACTTCATCCACGTTCACC. Samples from all three timepoints per person were assayed in the same 384-well plate.

Statistical analysis

The software used for analysis was IBM SPSS version 26. Background data are described as means and standard deviations, or frequencies and percentages, and Chi-Square or Fishers exact tests were calculated for descriptive background measures. The TA variable was ln-transformed due to significant skewness. Partial correlations were used to assess how the TL (adjusted for age) and TA correlated with pain intensity, spread and psychological outcomes at baseline. In addition, we used an analytic approach similar to Hassett et al [5], where the groups were divided into "high pain/low pain"; "high HAD score/low HAD score"; and "high WHOQOL-SRPB score/low WHOQOL-SRPB score" (the group mean score was used as cut-off), and ANCOVA analyses adjusted for age were conducted with TL and TA as dependent variables. A potential mediation model was conducted in an exploratory fashion following the standard mediation model of Baron and Kenny [44]. Direct effects were modeled in univariate regression models. Beta-weights, i.e. partial correlations coefficients were given for the different paths. This model was an ad-hoc decision after we saw the data pattern, because we wanted to explore the data further. It has to be viewed as a preliminary additional analysis to clarify relationships. We mark significance levels

according to a p-level of .005 corrected for multiple testing, which is in accordance with a recommended level for new discoveries [45]. But due to the exploratory nature of the study, we do not emphasize significances, but rather effect size, i.e. the extent of the correlation [46].

Results

Sample characteristics

The socio-demographic background variables and pain variables of participants are presented in Table 1. The majority of the participants were female, from Sweden and currently working. Most had upper secondary school or university education. Regarding pain, 82% reported it as constant and high, with an average pain intensity of 7.1 (out of 10) and a mean duration of nearly 12 years. With this amount of pain 60% were taking prescription drugs for pain and various other types of medications. Almost one third of the participants were using herbals and other supplements. In order to assess representativeness of our sample it was compared to the target population and hence Table 2 presents essential characteristics of the sample and target population, with numbers from a report by SQRP [47]. Table 3 presents the differences in participant demographic and pain variables from the different sites, where differences in distributions and means are also presented.

Table 1. Socio-demographic background and pain variables of participants

Variable	n	Score	SD
Gender	84		
**Female**	84	85.7%	
**Male**	84	14.3%	
Age	84	47.6	10.3
Country of birth	84		
**Sweden**	84	77.4%	
**Other Nordic countries**	84	3.6%	
**Europe**	84	9.5%	
**Outside Europe**	84	9.5%	
Education	84		
**Primary schooling**	84	7.1%	
**Upper secondary school**	84	64.3%	
**University**	84	22.6%	
**Other**	84	6.0%	
Working	82	87.8%	
Blood pressure	77		
**Systolic**	77	120.4 mmHg	17.3
**Diastolic**	77	76.9 mmHg	10.8
BMI	77	27.6	10.3
ICD-10 diagnosis	84		
**M-diagnoses**	84	66.7%	
**R-diagnoses**	84	19.0%	
**Q-diagnoses**	84	8.0%	
**F-diagnoses**	84	7.1%	
**Other diagnoses**	84	2.4%	
Medication	84		
**Prescription drugs for pain**	84	61%	
**Prescription drugs for other**	84	45.2%	
**Non-prescription drugs for pain**	75	28%	
**Non-prescription drugs for other**	75	10.7%	
**Herbals**	77	26%	
Type of pain	83		
**Constant**	83	81.9%	
**Periodic**	83	18.1%	
Pain duration	81	11.8	10.5
Pain intensity NRS	83	7.1	1.5
Pain spread	83	19.1	9.0

BMI: Body Mass Index, ICD-10: International Classification of Diseases, M-diagnoses: diseases of the musculoskeletal system and connective tissue, R-diagnoses: symptoms, signs and abnormal clinical and laboratory findings not elsewhere classified, Q-diagnoses: congenital malformations, deformations and chromosomal abnormalities, F-diagnoses: Mental, Behavioral and Neurodevelopmental disorders, NRS: numerical rating scale.

Table 2. Essential sample characteristics in comparison with the target population

Variable	Sample score	Target population score*
**Age**	47	44
**Gender F/M**	86/14%	79/21%
**Working now**	88%	67%
**Nationality Sweden/other**	81/19%	82/18%
**Senior high school**	64%	41%
**University**	23%	42%
**BMI**	28	27
**Pain duration**	12 years	9 years
**Pain intensity**	7.1	4.1
**Pain spread**	19	15

*Based on Swedish National Quality Registry for Pain Rehabilitation 2020

Table 3: Differences in demographic and pain variables in the two patient cohorts (Vidar Clinic and Linköping University Hospital)

	Clinic	N	Mean	p-value (t-test)
**Sex**	Vidar	51	96 (% female)	0.001 (Fisher's exact)
	Linköping	33	70 (% female)	
**Age**	Vidar	51	50.50	0.001
	Linköping	33	43.17	
**Pain duration (years)**	Vidar	49	15.39	<0.001
	Linköping	32	6.38	
**Pain intensity**	Vidar	51	6.95	0.43
	Linköping	32	7.22	
**Pain spread**	Vidar	51	22.53	<0.001
	Linköping	32	13.69	

Patient reported outcome measures

All PROM's were normally distributed. The PROM's for *clinical pain* including pain intensity, pain duration, pain spread, and medication are presented in table 1. All other PROM's, including

subscales are presented in Table 4. To better understand the interrelatedness of different PROM's, correlations were calculated amongst all the questionnaires and pain measures and are presented in Table 5.

Table 4. Patient Reported Outcome Measures including subscales and scoring

Questionnaire	n	Items, n	Scoring	Min-max	Mean	Std. Dev
**HAD scale score**	84	14	0-3	0-42	19.17	8.49
HAD scale anxiety	84	7	0-3	0-21	10.13	5.10
HAD scale depression	84	7	0-3	0-21	9.04	4.33
**EQ5D VAS**	82	1	0-100	0-100	40.77	17.37
**EQ5D Index value**	84	5	1-3	5-15	0.26	0.32
**FFMQ score**	80	29	1-5	29-145	91.95	15.12
FFMQ non-reactivity	80	6	1-5	6-30	17.80	3.94
FFMQ observe	80	7	1-5	7-35	24.40	5.02
FFMQ acting with awareness	80	5	1-5	5-25	14.55	3.87
FFMQ describe	80	6	1-5	6-30	19.26	4.80
FFMQ non-judging	80	5	1-5	5-25	16.03	4.22
**CPAQ score**	79	8	0-6	0-48	22.14	7.18
CPAQ pain willingness	79	4	0-6	0-24	11.72	5.34
CPAQ activity engagement	79	4	0-6	0-24	10.42	4.63
**WHOQOL-SRPB score**	75	32	1-5	32-160	92.00	26.44
WHOQOL-SRPB spiritual connection	75	4	1-5	4-20	10.27	4.98
WHOQOL-SRPB meaning and purpose in life	75	4	1-5	4-20	12.85	3.49
WHOQOL-SRPB experiences of awe and wonder	75	4	1-5	4-20	10.52	4.62
WHOQOL-SRPB wholeness and integration	75	4	1-5	4-20	12.84	3.83
WHOQOL-SRPB spiritual strength	75	4	1-5	4-20	12.92	3.23
WHOQOL-SRPB inner peace	75	4	1-5	4-20	10.93	4.04
WHOQOL-SRPB hope and optimism	75	4	1-5	4-20	10.51	3.38
WHOQOL-SRPB faith	75	4	1-5	4-20	11.16	3.37

HAD: Hospital anxiety depression scale, EQ5D (VAS): Euro Quality of Life 5 Dimensions (visual analogue scale), FFMQ: Five Facets Mindfulness Questionnaire, CPAQ: Chronic Pain Acceptance Questionnaire, WHOQOL-SRPB: WHO Quality of Life –Spiritual, Religious and Personal Beliefs.

Table 5. Correlations amongst questionnaires and pain measures

	**HAD score**	**EQ5D index**	**EQ5D VAS**	**FFMQ score**	**CPAQ score**	**WHO score**	**Pain intensity**	**Pain duration**	**Pain spread**
**HAD score**									
**EQ5D index**	-.300								
**EQ5D VAS**	-.347*	.378*							
**FFMQ score**	-.470*	.019	.019						
**CPAQ score**	-.292	.212	.366*	-.005					
**WHO score**	-.347*	.189	.236	.485*	.064				
**Pain intensity**	.292	-.333*	-.270	-.230	-.160	-.235			
**Pain duration**	.011	.051	.004	.070	-.101	.166	-.108		
**Pain spread**	.298	-.104	-.158	.008	-.273	.090	.095	.330*	

Abbreviations: Hospital Anxiety and Depression scale (HAD), Euroqol 5 Dimensions (EQ5D), Visual Analogue Scale (VAS), Five Facets Mindfulness Questionnaire (FFMQ), Chronic Pain Acceptance Questionnaire (CPAQ), WHO Quality of Life Spiritual, Religious and Personal Beliefs (WHO). *Correlation is significant at the 0.005 level (2-tailed)

Cellular measurements

The mean TL of the group was 1.163 (n=84, SD=0.412) and the group mean TA (ln transformed) was 2.064 TPG units/µL (n=80, SD=1.774). At group level, all routine blood test results were within normal reference intervals according to Swedish standards at Karolinska University Laboratory.

Telomere measurement correlations and associations with PROMs

TL was negatively associated with age (r=-.210, p=.055) and in an age-adjusted correlation, only P-cholesterol (r=.225, p=.041) and LDL-cholesterol (r=.218, p=.050) correlated with TL. No correlations were found between measures in blood samples and the TA. With self-report measures, a negative correlation was found between the TL and HAD score (r = -.219, p = .047) (Table 6). Also, a positive correlation was seen between the TL and WHOQOL-SRPB (r = .224, p = .052) (Table 6). Interestingly the TL was not correlated to any of the pain measures. The TA did not correlate with any of the self-reported questionnaires or pain measures. When the groups were divided into "high pain/low pain" and "high HAD score/low HAD score" (the group mean score was used as cut-off), and ANCOVA analyses adjusted for age were conducted with TL and TA as dependent variables, no associations were detected (for TA and HAD: Eta^2 = 0.00; p = 0.901; for TL and HAD: Eta^2 = 0.012; p = 0.32; for TA and pain: Eta^2 = 0.011, p = 0.357; for TL and pain: Eta^2 = 0.001; p = 0.78; for TA and WHOQOL-SRPB: Eta^2 = 0,008; p = 0,460; for TL and WHOQOL-SRPB: Eta^2 = 0,022; p = 0,225).

Table 6. Partial correlations with p-values, between TA/TL and self-report measures

	HAD	EQ5D index	EQ5D VAS	FFMQ	CPAQ	WHO	PI	PD	PS
TL	-.219 (.047)	-.003 (.977)	.141 (.211)	.131 (.250)	.189 (.089)	.224 (.052)	-.154 (.166)	.083 (.462)	-.188 (.090)
TA	-.042 (.709)	-.080 (.480)	-.100 (.383)	.039 (.738)	-.031 (.787)	.145 (.225)	-.177 (.119)	.181 (.113)	.222 (.049)

Abbreviations: Hospital Anxiety and Depression scale (HAD), Euroqol 5 Dimensions (EQ5D), Visual Analogue Scale (VAS), Five Facets Mindfulness Questionnaire (FFMQ), Chronic Pain Acceptance Questionnaire (CPAQ), WHO Quality of Life Spiritual, Religious and Personal Beliefs (WHO), Pain Intensity (PI), Pain Duration (PD), Pain Spread (PS), Telomere Length (TL), Telomerase Activity (TA).

Mediation effects

As the first order correlations and theoretical consideration suggested that the relationship between spiritual well-being (WHOQOL-SRPB) and TL might be mediated by the effect of the former on depression and anxiety (HAD scale) we inspected a potential mediation model in an exploratory fashion. First, the direct effects a, b and c were modeled in univariate regression models. Beta-weights, i.e. partial correlations coefficients are given for the different paths in Figure 1. Modelling the total effect using the mediation path, the interaction a*b (Total Effect = c + a*b), following the standard mediation model of Baron and Kenny [44] yields a total effect (beta = 0.008; p = .045), i.e. supports the idea of a mediation of the direct effect of spiritual well-being on TL by anxiety and depression (HAD scale). However, the indirect effect, the interaction a*b (ab = 0.002; beta = -.23; p = .15) is not significant.

Insert Figure 1 here

Discussion

To the best of our knowledge this is the first scientific study that explores the body-mind-spirit perspective of pain in an integrated and comprehensive manner, by investigating both telomeres, blood tests and questionnaires covering pain, coping, depression, mindfulness and spiritual well-being, including personal and religious beliefs.

Our typical study participant was a woman that rated her overall health poorly, with EQ5D VAS mean being 41 out of a maximum of 100. In comparison, the Swedish population norm (not standardized) is 83 out of 100 [48]. The EQ5D index value is 0.85 for the Swedish general population, whereas our participant mean score was 0.26 (SD 0.32). Our average participant had a

clinically probable risk for both anxiety and depression with a moderate pain coping capacity - more for pain willingness than for activity engagement. When aspects of mindfulness were reported, our average participant was struggling with acting with awareness or staying focused on the task at hand without becoming distracted. Mindfulness meditation could be beneficial as an intervention since it can improve pain, depression symptoms and quality of life with no risk of addiction [49]. On average, participants scored moderately on all facets of the WHOQOL-SRPB. Their sense of meaning and purpose in life together with a feeling of wholeness and integration was slightly more prominent while there was a lack of hope and optimism as well as few experiences of awe and wonder. Despite the obvious challenges to realize a universal instrument for spirituality that allow equitable comparison among people with different worldviews [50] the SRPB dimensions used in our study can be seen as related to spiritual quality of life in one way or another. Studies have repeatedly established positive links between spirituality, religion and health where for example religious involvement is correlated with better mental health in the areas of depression, substance abuse, and suicide [51]. It has also been shown that cardiovascular and cortisol stress responses of a sample of university students can be predicted by their secularity, religiosity, spirituality, and existential search [52]. Since spirituality seem to enhance quality of life and affective well-being in chronic pain patients, partly mediated through coping [53-55], health care interventions including spiritual, religious and personal meaning dimensions might be beneficial.

Interestingly, the blood samples did not deviate from normal reference intervals indicating that patients with severe and long-lasting pain paradoxically appear “perfectly healthy”. However, more complex proteomics or pro-inflammatory markers have been shown to deviate in chronic pain patients and correlate with other health aspects such as HAD scores [56].

Important to mention is the unbalanced gender ratio (86-14) in this sample, characteristic of the pain patient population nationally as well as globally, illuminating a gender issue. Women are noticeably overrepresented amongst patients living with chronic pain [57-59] and it has been highlighted as a gendered condition possibly subjected to medical gender bias [58]. Further adding to this, a report from 2018 requested by the Swedish Government concluded that there were close to zero (1 of 185) reviews with focus on women in this female dominated condition [59].

Regarding correlations, the participants' self-reported anxiety and depression had stronger relationships with measures of mindfulness and spiritual well-being than with pain measures. Indeed, our findings indicate that stronger spiritual, religious or personal beliefs may be protective of depression and anxiety, and that this may perhaps be mediated by increased mindfulness - presence in everyday life. However, our study design does not allow us to assess the direction of association, whereby it may also be so that decreased depression and anxiety instead enables increased mindfulness and spiritual, religious and personal beliefs.

One interesting finding in this study is that the WHOQOL-SRPB had a positive correlation to TL, meaning that a greater sense of spiritual well-being, meaning, purpose and wholeness might be linked with longer telomeres. Furthermore, our exploratory model supports the idea of a mediation of the direct effect of spiritual well-being on TL by anxiety and depression as measured by the HAD scale. The relationship between spiritual well-being and TL could indicate a weak relationship between PROM's and aspects of telomeres. As we had only a small sample, the correlations, though interesting from a conceptual point of view, did not reach conventional significance thresholds. This likely indicates a power problem which underlines the need for replication with a larger data set. The HAD scale was negatively correlated with the TL, meaning

that higher depression/anxiety scores, could be linked to shorter telomeres. Similar findings have been described by Hassett et al [5] and Sibille et al [26].

Another finding is the absence of correlation between TL and the pain measures selected in this study. This is surprising since others have found statistically significant correlations between TL and pain measures [5, 21-24]. In contrast, TA had a positive relationship with pain spread, i.e. high TA was associated with higher pain spread. But again, this correlation is not large enough to reach a convincing significance level with the sample size we had available. This is ambiguous since TL is negatively associated with pain spread to a notable degree. Surprisingly, when repeating the cross table statistical analysis of Hassett et al [5] by grouping high pain-high depression in contrast to low pain-low depression we were still unable to reproduce the results with correlations between TL and high depression - high pain. For all lab tests, pain measures and other self-report measures it is the HAD scale and the WHOQOL-SRPB that emerge in correlation with TL.

In summary, what ultimately correlates with the genomic integrity within this sample of chronic pain patients is neither quantitative pain measures or scales, nor index numbers generated for health economy calculation purposes, but instead anxiety, depression and spiritual, religious and personal beliefs. And those, in turn, have a significant relation to both the single EQ5D VAS question and some of the self-report pain measures. While how patients feel is often dismissed in health care [8, 9], it seems to be mirrored by the state of genomic integrity as well as in self-reports in our study. This is in line with the model proposed by Wideman et al [10], with patients as experts on their own condition.

Although the recommendations by large organizations like IMMPACT is to use NRS as the core measurement for clinical pain [29], it doesn't relate to the genomic integrity of the participants

in this study. And while measures of emotional functioning are included in the recommendations for core outcome measures, the spiritual dimension of pain is absent from the international recommendations [29].

The global opioid crisis is a painful reality that is of foremost relevance to pain management. Opioids are commonly prescribed to chronic pain patients in spite of doubtful effectiveness as long-term treatment. Between 1999-2014 approximately 165 000 persons died of overdose related to opioid medications - only in the United States [60]. Moreover, during 2015 opioids were responsible for a third of the almost half a million global drug deaths [61]. And despite the relation to global morbidity, mortality and doubtful long-term effectiveness, 24 % of the participants in our sample of chronic pain patients had opioids prescribed for their pain. Our study gives reasons to suggest that it might be beneficial for health care to be guided by interventions also including the dimensions of emotional well-being and spiritual, religious and personal beliefs when it comes to pain management, i.e. a focus on promoting connection at the expense of addiction.

Increasingly, more patients are offered rehabilitation in so-called multimodal programs with psychological and physical activities and this biopsychosocial approach and model in pain management is currently recommended in Sweden [59, 62] , though without reference to spiritual aspects of health and pain. Given that TL is a good indicator for longevity and robustness of health [12] and that spiritual well-being is somewhat associated with TL this indicates a novel arena with potential in pain rehabilitation. Our findings are preliminary and warrant further scrutiny.

Limitations

A limitation with this study is its cross-sectional design with the innate inability to draw causal conclusions, and its comparatively small sample size. The latter was due to organisational restrictions that could not be remedied. However, the goal was rather to generate some preliminary insights, questions and hypotheses for future studies. Another apparent limitation is the lack of a control group. This is part of the nature of this study as patients cannot be randomised to become chronic pain patients. Only naturalistic cohorts of healthy, age matched controls would be possible, and we were unable to recruit such controls for this study for organisational reasons.

There are several biases in this project. Firstly, the two sites had different acceptance rates which can generate bias. Together, 45% of all eligible persons accepted participation which increases the risk of bias and raises the concern of the sample not being representative of the target population. Yet, by comparing our data to national health registry data [47] we can see that our sample was in fact representative of the target population, as well as assessing on what accounts they were not.

The double data entry and cross-checking and careful validation of the database before analysis improved methodological strength in this study. Also, the inclusion of two geographically separate locations as study sites with the aim of improving generalizability can be seen as a strength. In addition, since Complementary and Alternative Medicine (CAM) modalities are widely used by pain patients [63], the inclusion of The Vidar Clinic as a formal health care provider including certain CAM therapies can also been seen as a strength contributing to generalizability.

Another limitation is that other potential confounders apart from BMI, age or sex may have accounted for some of the results. We did not investigate the effect of tobacco or alcohol use, which are known to confound telomere measurements. Indeed, levels of exercise and nutrition and patients' medication should also be considered. Also, the initial preparation of the blood samples

used for TL and TA measurement were done by different staff at the two sites. However, the standard protocol was followed and when conducting analyses of the mononuclear cells in laboratory environment, the quality of blood sampling and initial preparation could be validated. Additionally, the randomisation of the telomere samples as well as blinding of laboratory personnel performing the tests added to methodological strengths and reduced bias.

The size of the correlations we found were rather small, between r = .2 and .3. Because of the small sample size and the comparatively large number of correlations, inherent in an exploratory project such as this one, we would not want to draw any definitive conclusions. But it seems to be an interesting hypothesis to follow up in a subsequent study whether the positive association between TL and spiritual well-being can be substantiated, as well as the negative association between depression and anxiety and TL. Also the positive correlation between TA and pain spread warrants further scrutiny. The former would be indicative of a protective effect of spirituality and meaning-making against physical disintegration. The latter would point to an association between repair processes and the spreading of pain. With such focussed hypotheses further studies could produce more confirmatory evidence than our data can provide.

Also, it should be noted that our mediation model is intuitive, but far from definitive. That spiritual well-being together with an interaction between SRPB and HAD should have a positive, albeit small, influence on telomere length certainly warrants further scrutiny. As our data were cross-sectional, it would be mandatory to have longitudinal data which would lend itself to a clear causal analysis, which our analysis cannot provide. Furthermore, one can argue that mediation is only demonstrated according to the original Baron and Kenny approach, but not according to more recent discussions [64, 65], which would require an independent interaction term to be significant.

However, we nevertheless think that this potential mediation might be interesting for other researchers to scrutinize in more detail.

Conclusion

In summary, chronic pain patients participating in this study confirm the view of a complex and holistic web of health dimensions that are affected by and associated with their suffering and a heavy burden of symptoms. Our findings show that telomeres tend to be shorter in more anxious and depressed pain patients, but not in patients with more self-reported pain. Intriguingly, spiritual well-being as measured with WHOQOL-SRPB, was in this sample of patients correlated with TL. TA was more pronounced in patients reporting higher pain spread. These findings are preliminary and need confirmation. This study confirms the notion that numerical estimations of pain may be too crude to capture the complexity of chronic pain, and the widespread use of such measures in health care and research can possibly be explained by an historical tendency to assess the effectiveness of pharmacological pain management. Considering the international opioid crisis, non-pharmacological and non-opioid treatments emerge as a way forward. We have indicated some reason to believe that the emotional and spiritual dimensions of pain and health is a neglected yet highly important aspect for the well-being and longevity of chronic pain patients and hence should be an additional focus in pain management and most importantly for further research.

Acknowledgements

The many anonymous patients are deeply appreciated for generously giving of body, mind and spirit, making this project possible. Also, the following persons are gratefully acknowledged: Annele Claeson for her general support and efforts during data entry, Eva-Britt Lind and Ulrika Wentzel Olausson for essential contribution in recruitment, data collection, and data entry. Vincent Millisher for substantial contribution in analysing telomere data, as well as editing and reviewing. Tobias Sundberg for contribution to database development and initial crude analysis. Sebastian Sauer for help with evaluating the mediation model. Maria Arman for insights and expertise on nursing and Johanna Hök Nordberg for integrated feedback on manuscript and both for thoughtful supervision. Linköping Smärtcentrum and The Vidar Clinic are acknowledged for smooth collaboration. We are also grateful for initial advice and guidance on genomic aspects of the project by Elizabeth Blackburn. Lastly a thank you to the unnamed reviewers who helped elevate this work.

Data availability statement

There are ethical restrictions on sharing our de-identified data set publicly since data contain potentially identifying sensitive patient information. The reasons are both the rules on confidentiality, primarily to protect the research subjects, and the GDPR regulations in Sweden.

References

[1] Leadley RM, Armstrong N, Lee YC, Allen A, Kleijnen J. Chronic diseases in the European Union: the prevalence and health cost implications of chronic pain. J Pain Palliat Care Pharmacother. 26(4) (2012) 310-25. doi: 10.3109/15360288.2012.736933.

[2] Bergman SP, Herrstrom P, Hogstrom K, Peterson IF, Svensson B, Jacobsson LT. Chronic musculoskeletal pain, prevalence rates, and sociodemographic associations in a Swedish population study, J. Rheumatol, 28(6) (2001) 1369-77.

[3] McBeth J, Symmons DP, Silman AJ, Allison T, Webb R, Brammah T, et al. Musculoskeletal pain is associated with a long-term increased risk of cancer and cardiovascular-related mortality. Rheumatology (Oxford), 48(1) (2009) 74-7. doi: 10.1093/rheumatology/ken424.

[4] Wolfe W, Hassett AL, Walitt B, Michaud K. Mortality in fibromyalgia: a study of 8,186 patients over thirty-five years. Arthritis Care Res (Hoboken). 63(1) (2011) 94-101. doi: 10.1002/acr.20301

[5] Hassett AL, Epel E, Clauw DJ, Haris RE, Harte SE, Kairys A, et al. Pain is associated with short leukocyte telomere length in women with fibromyalgia. J Pain. **13**(10) (2012) 959-69. doi: 10.1016/j.jpain.2012.07.003.

[6] Gerdle B, Bjork J, Henriksson C, Bengtsson A. Prevalence of current and chronic pain and their influences upon work and healthcare-seeking: a population study. J Rheumatol. 31(7) (2004) 1399-406.

[7] Reid KJ, Harker J, Bala MM, Truyers C, Kellen E, Bekkering GE, et al., J. Epidemiology of chronic non-cancer pain in Europe: narrative review of prevalence, pain treatments and pain impact. Current Medical Research and Opinion. **27**(2) (2011) 449-462. doi: 10.1185/03007995.2010.545813. Epub 2011 Jan 3.

[8] De Ruddere L, and Craig KD, Understanding stigma and chronic pain: a-state-of-the-art review. Pain. **157**(8) (2016) 1607-10. doi: 10.1097/j.pain.0000000000000512.

[9] Toye F, Seers K, Allcock N, Briggs M, Carr E, AndrewsJ, et al. Patients' experiences of chronic non-malignant musculoskeletal pain: a qualitative systematic review. Br J Gen Pract. **63**(617) (2013) 829-41.

[10] Wideman TH, Edwards RR, Walton DM, Martel MO, Hudon A, Seminowicz DA. The Multimodal Assessment Model of Pain: A Novel Framework for Further Integrating the Subjective Pain Experience Within Research and Practice. Clin J Pain. 35(3) (2019) 212-221. doi: 10.1097/AJP.0000000000000670.

[11] Blackburn E, Epel E, Lin J. Human telomere biology: A contributory and interactive factor in aging, disease risks, and protection. Science. 350(6265) (2015) 1193-8. doi: 10.1126/science.aab3389.

[12] Epel E, Blackburn E, Lin J, Dhabhar FS, Adler NE, Morrow JD, et al. Accelerated telomere shortening in response to life stress. Proc Natl Acad Sci U S A. 101(49) (2004) 17312-5. doi: 10.1073/pnas.0407162101. Epub 2004 Dec 1.

[13] Epel E, Lin J, Wilhelm FH, Wolkowitz OM, Cawthon R, Adler NE, et al. Cell aging in relation to stress arousal and cardiovascular disease risk factors. Psychoneuroendocrinology. 31(3) (2006) 277-87. doi: 10.1016/j.psyneuen.2005.08.011.

[14] Haycock PC, Heydon EE, Kaptoge S, Butterworth AS, Thompson A, Willeit P, Leucocyte telomere length and risk of cardiovascular disease: systematic review and meta-analysis. Bmj. 349 (2014) 4227. doi: https://doi.org/10.1136/bmj.g4227

[15] K.T. Sibille, Witek-Janusek L, Mathews HL, Fillingim RB. Telomeres and epigenetics: potential relevance to chronic pain. Pain. 153(9) (2012) 1789-93. doi: 10.1016/j.pain.2012.06.003.

[16] Blackburn E, Epel E. The telomere effect : a revolutionary approach to living younger, healthier, longer. Orion spring, London, 2017.

[17] Vaiserman A, Krasnienkov D. Telomere Length as a Marker of Biological Age: State-of-the-Art, Open Issues, and Future Perspectives. Front. Genet. 11:630186 (2021) doi: 10.3389/fgene.2020.630186.

[18] Epel E, Lin J, Dhabhar FS, Wolkowitz OM, Puterman E, Karan L, et al. Dynamics of telomerase activity in response to acute psychological stress. Brain Behav Immun, 24(4) 2010 531-9. doi: 10.1016/j.bbi.2009.11.018.

[19] Skvortsov DA, Zvereva ME, Shpanchenko OV, Dontsova OA. Assays for detection of telomerase activity. Acta Naturae. 3(1) (2011) 48-68.

[20] Daubenmier J, Lin J, Blackburn E, Hecht FM, Kristeller J, Maninger N, et al. Changes in stress, eating, and metabolic factors are related to changes in telomerase activity in a randomized mindfulness intervention pilot study. Psychoneuroendocrinology. 37(7) (2012) 917-28.

[21] Le Maitre CL, Freemont AJ, Hoyland JA. Accelerated cellular senescence in degenerate intervertebral discs: a possible role in the pathogenesis of intervertebral disc degeneration. Arthritis Res Ther. 9(3) (2007) R45. doi: 10.1186/ar2198.

[22] Dechsupa S, Yingsakmongkol W, Limthongkul W, Singhatanadgige W, Honsawek S. Relative telomere length and oxidative DNA damage in hypertrophic ligamentum flavum of lumbar spinal stenosis. PeerJ. 6: (2018) e5381. doi: 10.7717/peerj.5381

[23] Maeda T, Kudo Y, Horiuchi T, Makino N. Clinical and anti-aging effect of mud-bathing therapy for patients with fibromyalgia. Mol Cell Biochem. 444(1-2) (2018) 87-92. doi: 10.1007/s11010-017-3233-4.

[24] Sibille KT, Chen H, Bartley EJ, Riley J, Glover TL, King CD, et al. Accelerated aging in adults with knee osteoarthritis pain: consideration for frequency, intensity, time, and total pain sites. Pain Rep. 2(3) (2017) e591. doi: 10.1097/PR9.0000000000000591.

[25] Steward AM, Morgan JD, Espinosa JP, Turk DC, Patel KV. Chronic Pain and Telomere Length in Community-Dwelling Adults: Findings From the 1999 to 2002 National Health and Nutrition Examination Survey. J Pain. 18(12) (2017) 1517-1525. doi: 10.1016/j.jpain.2017.08.006.

[26] Sibille KT, Langaee T, Burkley B, Gong Y, Glover TL, King C, et al. Chronic pain, perceived stress, and cellular aging: an exploratory study. Mol Pain. 8 (2012) 12. doi: 10.1186/1744-8069-8-12.

[27] Lucchetti G. *Spirituality, Religiousness and Health [Elektronisk resurs]*. 2019: Springer International Publishing.

[28] Lucchetti G, Koenig HG, Lucchetti ALG. Spirituality, religiousness, and mental health: A review of the current scientific evidence. World J Clin Cases. 16;9(26) (2021) 7620-7631. doi: 10.12998/wjcc.v9.i26.7620.

[29] Dworkin RH, Turk DC, Farrar JT, Haythornthwaite JA, Jensen MP, Katz NP, et al. Core outcome measures for chronic pain clinical trials: IMMPACT recommendations. Pain. 113(1-2) (2005) 9-19. doi: 10.1016/j.pain.2004.09.012.

[30] McCracken LM, Vowles KE, and Eccleston C. Acceptance of chronic pain: component analysis and a revised assessment method. Pain. 107(1-2) (2004)159-66. doi: 10.1016/j.pain.2003.10.012.

[31] Rovner GS, Arestedt K, Gerdle B, Börsbo B, McCracken LM. Psychometric properties of the 8-item Chronic Pain Acceptance Questionnaire *(CPAQ-8) in a Swedish chronic pain cohort*. J Rehabil Med. 46(1) (2014) 73-80. doi: 10.2340/16501977-1227.

[32] Olsson SJ, Ekblom O, Andersson E, Börjesson M, Kallings LV. Categorical answer modes provide superior validity to open answers when asking for level of physical activity: A cross-sectional study. Scand J Public Health. 44(1) (2016) 70-6. doi: 10.1177/1403494815602830.

[33] EuroQol-Group, EuroQol--a new facility for the measurement of health-related quality of life. Health Policy. 16(3) (1990) 199-208. doi: 10.1016/0168-8510(90)90421-9.

[34] Bjelland I, Dahl AA, Haug TT, Neckelman D. The validity of the Hospital Anxiety and Depression Scale. An updated literature review. J Psychosom Res. 52(2) (2002) 69-77. doi: 10.1016/s0022-3999(01)00296-3.

[35] Lisspers J, Nygren A, and Söderman E. Hospital Anxiety and Depression Scale (HAD): some psychometric data for a Swedish sample. 96(4) (1997) 281-286. doi: 10.1111/j.1600-0447.1997.tb10164.x.

[36] LoMartire R, Äng BO, Gerdle B, Vixner L. Psychometric properties of Short Form-36 Health Survey, EuroQol 5-dimensions, and Hospital Anxiety and Depression Scale in patients with chronic pain. Pain. 161(1) (2020) 83-95. doi: 10.1097/j.pain.0000000000001700.

[37] Skevington SM, Gunson KS, O'Connell KA. Introducing the WHOQOL-SRPB BREF: developing a short-form instrument for assessing spiritual, religious and personal beliefs within quality of life. Qual Life Res. 22(5) (2013) 1073-83. doi: 10.1007/s11136-012-0237-0.

[38] Melder C. Vilsenhetens epidemiologi -en religionspsykologisk studie i existentiell folkhälsa [The epidemiology of lost meaning − A study in psychology of religion and existential public health in a Swedish context], in Acta Universitatis Upsaliensis, Psychologia et Sociologia Religionum 25th [Doctoral dissertation] 2011, Uppsala University Uppsala, Sweden. p. 305.

[39] Baer RA, Smith GT, Hopkins J, Krietemeyer J, Toney L. Using self-report assessment methods to explore facets of mindfulness. Assessment. 13(1) (2006) 27-45. doi: 10.1177/1073191105283504.

[40] Lilja JL, Frodi-Lundgren A, Hanse JJ, Josefsson T, Lundh LG, Sköld C, et al. Five Facets Mindfulness Questionnaire--reliability and factor structure: a Swedish version. Cogn Behav Ther. 40(4) (2011) 291-303.

[41] Hou M, Xu D, Bjorkholm M, Gruber A. Real-time quantitative telomeric repeat amplification protocol assay for the detection of telomerase activity. Clin Chem. 47(3) (2001) 519-24.

[42] O'Callaghan NJ, Fenech M. A quantitative PCR method for measuring absolute telomere length. Biol Proced Online. 13 (2011) 3. doi: 10.1186/1480-9222-13-3.

[43] R.M. Cawthon, Telomere measurement by quantitative PCR. Nucleic Acids Res. **30**(10) (2002) e47. doi: 10.1093/nar/30.10.e47.

[44] Baron RM, Kenny DA. The moderator-mediator variable distinction in social psychological research: conceptual, strategic, and statistical considerations. J Pers Soc Psychol. **51**(6) (1986)1173-82. doi: 10.1037//0022-3514.51.6.1173.

[45] Benjamin DJ, Berger JO, Johannesson M, Nosek, BA Wagenmakers EJ, Berk R, et al. Redefine statistical significance. Nat Hum Behav. 2(1) (2018) 6-10. doi: 10.1038/s41562-017-0189-z.

[46] Gigerenzer G. Mindless statistics. Journal of Socio-Economics, 33 (2004) 587-606. https://doi.org/10.1016/j.socec.2004.09.033

[47] SQRP, *Bilaga rapport 2020:1 Specialistvård: patienter som avslutats under 2019*. 2020.

[48] Szende A, Self-Reported Population Health: An International Perspective based on EQ-5D [Elektronisk resurs]. 2014.

[49] Hilton L, Hempel S, Ewing BA, Apaydin E, Xenakis L, Newberry S, et al. Mindfulness Meditation for Chronic Pain: Systematic Review and Meta-analysis. Ann Behav Med. **51**(2) (2017)199-213. doi: 10.1007/s12160-016-9844-2.

[50] Hammer JH, Wade NG, Cragun RT. Valid assessment of spiritual quality of life with the WHOQOL-SRPB BREF across religious, spiritual, and secular persons: A psychometric study. Psychology of Religion and Spirituality, 12(4), 440–450. https://doi.org/10.1037/rel000026650.

[51] Bonelli RM, Koenig HG. Mental Disorders, Religion and Spirituality 1990 to 2010: A Systematic Evidence-Based Review. *J Relig Health* **52**, 657–673 (2013). https://doi.org/10.1007/s10943-013-9691-4

[52] Schnell T, Fuchs D, Hefti R. Worldview Under Stress: Preliminary Findings on Cardiovascular and Cortisol Stress Responses Predicted by Secularity, Religiosity, Spirituality, and Existential Search. *J Relig Health* **59**, 2969–2989 (2020). https://doi.org/10.1007/s10943-020-01008-5

[53] Biccheri E, Roussiau N, Mambet-Doue C. Fibromyalgia, Spirituality, Coping and Quality of Life. J Relig Health 55 (4) (2016), 1189-97. doi: 10.1007/s10943-016-0216-9.

[54] Dedeli O, Kaptan G. Spirituality and Religion in Pain and Pain Management. Health Psychol Res. **1**(3) (2013) e29. doi: 10.4081/hpr.2013.e29

[55] Moreira-Almeida A, Koenig HG. Religiousness and spirituality in fibromyalgia and chronic pain patients. Curr Pain Headache Rep. **12**(5) (2008) 327-32. doi: 10.1007/s11916-008-0055-9.

[56] Gerdle B, Backryd E, Falkenberg T, Lundström E, Ghafouri B. Changes in inflammatory plasma proteins from patients with chronic pain associated with treatment in an interdisciplinary multimodal rehabilitation program - an explorative multivariate pilot study. Scand J Pain. **20**(1) (2019) 125-138. doi: 10.1515/sjpain-2019-0088.

[57] Ahlgren C, Fjellman-Wiklund A, Hamberg K, Johansson EE, Stålnacke BM, The meanings given to gender in studies on multimodal rehabilitation for patients with chronic musculoskeletal pain – a literature review. Disability and Rehabilitation. **38**(23) (2016) 2255-2270. doi: 10.3109/09638288.2015.1127435.

[58] Smirthwaite G, Tengelin E, Borrman T. (O)jämställdhet i hälsa och vård. (rev uppl) Stockholm: Sveriges kommuner och regioner; 2014.

[59] Statens beredning för medicinsk och social utvärdering. Behandling av långvariga smärttillstånd med fokus på kvinnor. En kartläggning av systematiska översikter. Vol. SBU-rapport nr 301. 2019: Stockholm: Statens beredning för medicinsk utvärdering (SBU), 2019.

[60] Dowell D, Haegerich TM, Chou R. *CDC Guideline for Prescribing Opioids for Chronic Pain—United States, 2016.* JAMA, 2016. **315**(15): p. 1624-1645.

[61] UN, *World drug report 2017*. 2017, Vienna: United Nations, Office on Drugs and Crime.

[62] Statens beredning för medicinsk och social utvärdering, *Rehabilitering vid långvarig smärta: en systematisk litteraturöversikt.* Stockholm: Statens beredning för medicinsk och social utvärdering (SBU), 2010.

[63] Rosenberg EI, Genao I, Chen I, et al., *Complementary and alternative medicine use by primary care patients with chronic pain.* Pain Med, 2008. **9**(8): p. 1065-72.

[64] K. Preacher, & A. Hayes. SPSS and SAS procedures for estimating indirect effects in simple mediation models. Behavior Research Methods, Instruments, & Computers in Human Behavior. 36, (2004) 717-731.

[65] A.F. Hayes, Beyond Baron and Kenny: Statistical mediation analysis in the new millennium. *Communication Monographs. 76*(4) (2009) 408–420. https://doi.org/10.1080/03637750903310360

Figure Click here to access/download;Figure;Figure 1. Mediation model.pdf

Figure 1. Mediation model

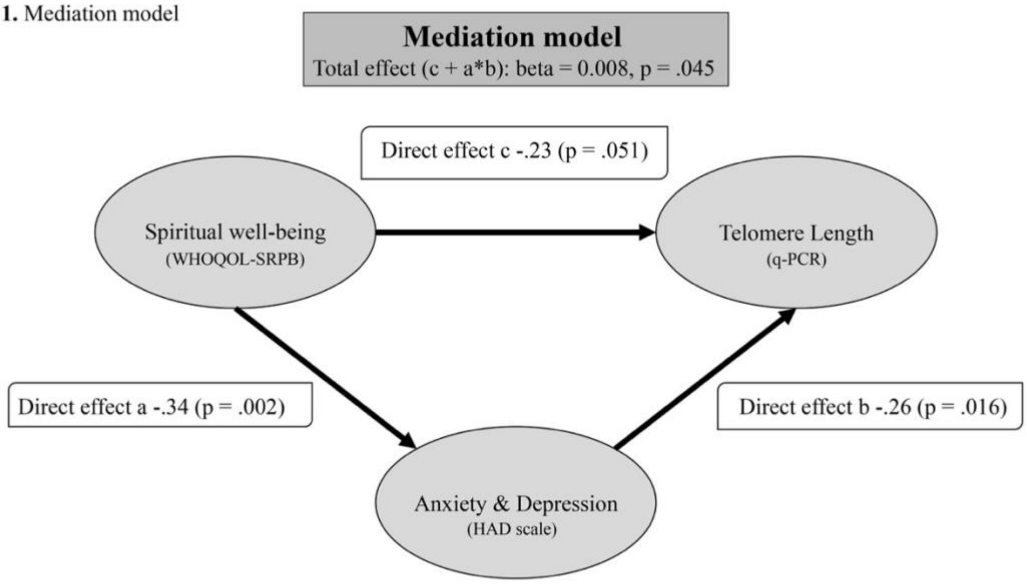

WHOQOL-SRPB: WHO Quality of Life –Spiritual, Religious and Personal Beliefs, HAD scale: Hospital Anxiety and Depression Scale; q-PCR: quantitative Polymerase Chain Reaction.

Click here to access/download
Supporting Information
Regional Ethical Board for Stockholm .pdf

~~Title Page~~Title Page

Exploring emotional well-being, ~~personal,~~ spiritual, ~~and~~ religious and personal beliefs and telomere length in chronic pain patients - A pilot study with cross-sectional design

Linn Rönne-Peterséna~~, Msc~~[1], M ~~linn.ronne-petersen@ki.se,~~
~~M~~aria Niemi~~, PhD~~[2] ~~maria.niemi@ki.se~~
, H~~H~~arald Walach~~h, Professor~~[3] ~~hw@chs-institute.org~~
, C~~C~~atharina Lavebratt~~, Assoc Prof~~[4] ~~catharina.lavebratt@ki.se~~
, L~~L~~iu L. Yang~~, M.D, PhD~~[4] ~~liu.yang@ki.se~~
, B~~B~~jörn Gerdle~~, Professor~~[5] ~~bjorn.gerdle@liu.se~~
, B~~B~~ijar Ghafouri~~, Assoc Prof~~[5] ~~bijar.ghafouri@liu.se~~
, T~~T~~orkel Falkenberg~~, Assoc Prof~~[1]* ~~torkel.falkenberg@ki.se +46706183868~~

~~*Corresponding author~~

~~AFFILIATIONS:~~

[1]*Deparment of Neurobiology, Care sciences and Society, Division of Nursing 2, Karolinska Institutet, ~~171 77,~~ Stockholm, Sweden.*

[2]*Department of Global Public Health, K9 FHE Dalman, Karolinska Institutet, ~~171 77~~ Stockholm, Sweden*

[3]*Change Health Science Institute, Berlin, Germany*

[4]*Department of Molecular Medicine and Surgery, Karolinska Institutet and Center for Molecular Medicine, ~~171 76~~ Stockholm, Sweden*

[5]*Pain and Rehabilitation Centre, and Department of Health, Medicine and Caring Sciences, Linköping University, ~~581 83~~ Linköping, Sweden*

*Corresponding author
E-mail: torkel.falkenberg@ki.se

Formatted: Font: 18 pt
Formatted: Numbering: Continuous
Formatted: Swedish (Sweden)
Field Code Changed
Formatted: Swedish (Sweden)
Formatted: Swedish (Sweden)
Formatted: English (United Kingdom)
Formatted: English (United Kingdom)
Field Code Changed
Formatted: Swedish (Sweden)
Formatted: Font color: Text 1, Swedish (Sweden)
Formatted: Swedish (Sweden)
Field Code Changed
Formatted: English (United States)
Formatted: English (United States)

~~ABSTRACT~~ Abstract

Formatted: Font: 18 pt

~~*Introduction:*~~ Living with chronic pain is associated with substantial suffering and high societal costs. Patient reported outcomes (PROM's) and effects on cellular ageing should be considered in pain management. The aim of this study was to explore correlations of PROM's and cellular ageing (telomere length [TL] and telomerase activity [TA] measures) amongst patients with chronic non-malignant pain. ~~*Methods:*~~ This was an explorative pilot study with cross-sectional design and recruitment was done at two pain rehabilitation facilities in Sweden, with inpatient setting/integrative care and outpatient setting/multimodal care, respectively. Eighty-four patients were enrolled by referral to pain rehabilitation in Sweden. Main outcome measures collected after admission in addition to TL and TA were the following PROMs: Numerical Rating Scale, Chronic Pain Acceptance Questionnaire, Hospital Anxiety and Depression scale, Five Facets Mindfulness Questionnaire, WHO Quality of Life - Spiritual, Religious and Personal Beliefs and EuroQol 5 Dimensions. ~~*Results:*~~ All the PROM's showed evidence of poor health status. TL correlated negatively with Hospital Anxiety and Depression ($r = -.219$, $p = .047$) and positively with WHO Quality of Life -Spiritual, Religious and Personal Beliefs ($r = .224$, $p = .052$). TL did not correlate with any of the pain measures. TA correlated positively with pain spread ($r = .222$, $p = .049$). A mediation of the direct effect of spiritual well-being on TL by anxiety and depression could be shown ($b = 0.008$; $p = .045$). ~~*Conclusion:*~~ The correlations between TL and spiritual well-being and anxiety and depression suggest some importance of emotional and spiritual dimensions in pain management, with implications for cellular aging, which needs further confirmation.

ClinicalTrials.gov Identifier: NCT02459639

KEYWORDS

Chronic pain, Emotional health, Spirituality, Telomere length, Telomerase activity, Integrative Health Care

~~1. INTRODUCTION~~Introduction

Formatted: Font: 18 pt

~~1.1.~~ Chronic pain as a personal and societal condition

Formatted: Font: 16 pt

Chronic pain constitutes considerable personal suffering for patients living with it, and poses a great challenge for the healthcare system with high economic impact on society [1, 2]. Chronic pain has been described as an enigmatic phenomenon associated with substantial negative psychosocial factors and physical ill-health [3-5]. With 19-50 % of the European population affected, chronic pain is common, and in the female population the prevalence is at least 10 % higher [2, 6, 7]. Patients' reality of complex health issues is often misunderstood and misinterpreted by the healthcare system, leading to feelings of stigmatization and forcing the patients to seek healthcare repeatedly [8, 9]. Leadely et al [1] conclude that chronic pain is neither sufficiently prioritized in policies nor in budgets. Why such a widespread and incapacitating condition isn't more prioritized seems at large unclear.

~~1.2.~~ Measuring pain

Formatted: Font: 16 pt

Despite the complex nature of pain and the subsequent need for a wider approach to study it, most pain research has mainly focused on pain intensity and duration [10]. In contrast, Wideman et al [10] have proposed a comprehensive model for how to integrate the nature of subjectivity and objectivity in pain, while at the same time arguing that we can neither measure the largest part of pain, namely the *pain experience (inner experience)*, since it is non-observable and deeply personal. However, what research and clinicians can quantify are *pain measures* (subjective self-reported or objective non-self-reported), preferably mindful of *what* is being measured, i.e. not pain itself, but a limited part of the pain phenomena.

~~1.3.~~ Telomeres, cellular ageing and pain

Formatted: Font: 16 pt

Telomeres are specific, protective structures at the ends of our chromosomes that are crucial for preventing genetic information from being lost during cell division [11]. Shortened telomere length (TL) is considered a predictor for a number of age-related diseases and mortality [12-14], although it is also affected by factors *independent* of chronological age [14-16]. In addition, TL shortening tends to be more the result of cumulative life stress and age-related diseases [17]. The enzyme telomer*ase* functions as a buffer against the cellular aging in the nucleus, steadily re-building the telomeres by adding on base pairs [11, 16]. Changes in TL can take months or years to be detectable, while telomerase activity (TA) is more dynamic and linked to short-term changes. Changes in ~~Sometimes~~ TA ~~is a signal~~ enzyme levels have implications for telomere length and protection of the chromosomes ~~for repair activity, or an indicator of a lack of such activity~~ [18, 19], making it suitable for examining more ~~direct~~ immediate effects in time of for example certain lifestyle factors ~~on~~ or health-promoting behaviors ~~the cellular ageing process~~ [20].

Formatted: English (United States)

Formatted: English (United States)

Formatted: English (United States)

A handful articles address the association between chronic pain and cellular ageing, whereas reports on pain and measures of TA are lacking. The majority of studies find significant correlations between chronic pain or degenerative tissue (associated with pain) and decreased TL [5, 21-24]. There are also studies not showing significant correlations [25, 26]. Notably, one study observed changes in TL when looking at combinations of pain and high and low stress, arguing that a combined effect of the two may accelerate cellular ageing [26]. It has been estimated that chronic pain shortens life by 4-5 years (as measured in loss of base pairs) and combined with depression it adds up to 6 years [5]. Hence,

r~~R~~esearch aiming to investigate correlations between emotional well-being, pain experiences and cellular aging are urgently needed and could contribute to ~~cater for~~ new ways of developing and evaluating chronic ~~different health~~pain ~~care~~ interventions. ~~Multimodal~~ Health care interventions which ~~inclu~~include~~ding~~ elements of emotional well-being and spiritual health are important ~~elements of~~in nursing care and are emerging as potential health services components [27] with hypothetical implications for stress, mental health and pain management [27, 28] and even cellular aging.

Formatted: Font: Not Italic

Given these challenges and opportunities, t~~T~~he aim of this study was to explore correlations of Patient Reported Outcome Measurements [PROM's] and markers of cellular ageing amongst patients with chronic non-malignant pain. We used PROM's of clinical pain, physical and emotional functioning, ~~personal,~~ spiritual, ~~and~~ religious and personal beliefs, mindfulness and quality of life. We used TL and TA as measures of cellular ageing and potential mediators between PROMS and cellular ageing were explored.

~~2.~~ M~~ETHODS~~ ethods

Formatted: Font: 18 pt

~~2.1.~~ Participant recruitment

Formatted: Font: 16 pt

The participants in this study were individuals suffering from chronic pain conditions referred to tertiary specialist centers and enrolled at either The Vidar Clinic, Järna *or* Pain and Rehabilitation Center, University Hospital, Linköping between June 2015 and December 2017. The study was preregistered (ClinicalTrials.gov Identifier NCT02459639), and ethically approved by the Regional Ethics Committee in Stockholm (EPN 2014/953-31/1). ~~The study was preregistered (ClinicalTrials.gov Identifier NCT02459639).~~ After oral and written information, participants

Formatted: Font: Times New Roman, 12 pt

~~could make~~made an informed ~~written~~ decision whether to participate or not with no implications for their care. Inclusion criteria were ICD-10 M79, *OR* chronic pain (>3 months) in neck/shoulders and/or lower back or generalized pain (e.g. fibromyalgia), ≥18 years of age, fluent in Swedish, from Stockholm, Sörmland or Östergötland county councils. Comorbidities were allowed except for psychotic illness, addiction, and cancer.

In all, 57 patients at The Vidar Clinic and 138 patients at Linköping were invited to the study, of which 51 respectively 35 patients showed interest to participate. Of these, 51 respectively 33 patients met the inclusion criteria and agreed to participate. The final analysed sample consisted of 84 participating patients. The size of the Vidar Clinic was smaller compared to the Rehabilitation Center, University hospital, Linköping and hence explains the differences in recruitment proportions.

~~2.2.~~ Data collection

Formatted: Font: 16 pt

This was an explorative pilot study with cross-sectional design, looking into clinical characteristics and correlations in a sample of persons with chronic non-malignant pain. Measures were collected after admission. Because of its exploratory nature, no sample size was calculated but a minimum of 70 enrolled patients was strived for, which allows to document an effect size of r = .3 with 72% power and reaches a power of 80% with r = .32. These are the ranges of correlations that were deemed likely and clinically important. Patients were recruited from two geographic locations – one with an out-patient setting (Linköping) and one with an in-patient setting (The Vidar Clinic). The same standard protocols for blood sampling were used at both sites, with the patients in a fasting state and where samples were collected after admission. Questionnaires were distributed at the time of the blood sampling. Double data entry was practiced for quality reasons and the

completed database was then validated by cross-checking 12% of all data, whereby 0.7% were found to be incorrect due to small systematic errors. Those errors were controlled and adjusted for in the whole dataset before analysing.

~~2.3.~~ Measurements

Formatted: Font: 16 pt

The measurements of this study comprised of TA and TL and PROM's of clinical pain, physical and emotional functioning, ~~personal,~~ spiritual, ~~and~~ religious and personal beliefs, mindfulness and quality of life. In addition, demographics, anthropometrics, blood pressure and blood samples were collected. A BMI between 18.5-24.9 kg/m^2 would be considered normal, as was a blood pressure <140/90 mmHg. The participants' blood samples were taken and sent for standard clinical analysis at Clinical Chemistry Laboratory at Linköping University Hospital and at Karolinska University Laboratory at Södertälje Hospital. The blood count was measured as well as the properties of the erythrocytes, leucocytes, thrombocytes, standard lipid status (triglycerides and cholesterol with quotas) and inflammatory profile (sedimentation rate, C-reactive protein). ~~(data not shown).~~ Also, levels of haemoglobin, glucose, sodium, potassium and creatinine were measured. ~~d (data not shown).~~ The IMMPACT recommendations for core outcome measurements in chronic pain clinical trials [29] were considered and applied heading-wise, but not strictly followed in regard to the scales used. Some questionnaries were replaced due to being validated in Sweden and others were added due to relevance for this study.

Clinical pain was assessed by means of intensity using the Numerical Rating Scale [NRS] (0 = no pain, 10 = worst possible pain) which is a commonly used instrument, included in the Swedish National Quality Registry for Pain Rehabilitation [SQRP] and recommended by

IMMPACT [29], A score <4 translates to mild pain, 4-6 moderate and >7 severe pain. Pain duration (years, months and days) and pain spread was also assessed by means of well established self-report questionaire from SQRP. In addition, the Chronic Pain Acceptance Questionnaire [CPAQ] ($\alpha \geq .80$), with the subscales of *pain willingness* and *activity engagement* was used because of its emphasis on aspects of coping, which is a focus area within pain research [30, 31]. In accordance with recommendations from Dworkin et al [29], patient usage of rescue analgesics (fast-acting medications) were registered. The *physical functioning* was evaluated with the Swedish National Board of Health and Welfare's indicator questions on physical activity [FYS], recently validated [32], as well as EUROQOL 5 Dimensions-3L [EQ5D]($\alpha > .85$)[33]. The latter is an established measure of quality of life, including aspects of physical functioning. The EQ5D index value is a single number calculated from the different health states obtained within the questionnaire. This number is commonly ranging from 0 to 1, although negative numbers are possible, and it reflects how good or bad the health state is in comparison with the preferences of a general population in a specific country. The related EQ5D VAS is a single question with the score range 0-100, going from worst possible to best possible health [33]. *Emotional functioning* was measured with the Hospital Anxiety and Depression Scale [HAD Scale] ($\alpha > .89$), a reliable and valid instrument with subscales measuring *depression* and *anxiety* [34], validated in Sweden [35] and where high values indicate psychological distress [36]. *Participant ratings of global improvement and satisfaction with treatment*, as well as *Symptoms and adverse events* were assessed qualitatively, and will be reported separately.

~~Personal, S~~spiritual, ~~and~~ religious and personal beliefs, are increasingly recognized as a distinctive, important, and cross-cultural concept in quality of life assessments [37]. Hence, in addition to the IMMPACT recommendations we assessed ~~personal ,~~ spiritual, ~~and~~ religious and

personal beliefs using the WHO Quality of Life, Spiritual, Religious and Personal Beliefs (WHOQOL-SRPB) (α = .85) questionnaire in its appropriate Swedish adaptation [38]. The questionnaire has the eight following subscales: *spiritual connection, meaning and purpose in life, experiences of awe and wonder, wholeness and integration, spiritual strength, inner peace, hope and optimism, and faith*. Mindfulness using Five Facets Mindfulness Questionnaire [FFMQ]) (α = .81) was included to complement the WHOQOL-SRPB to assess if improvements in health could be mediated by increased mindfulness. It has five subscales: *non-reactivity, observe, acting with awareness, describe* and *non-judging*. FFMQ is the most frequently used questionnaire for mindfulness [39] and has been validated in Sweden [40]. The information of number of items and min-max score for each questionnaire is found in Table 3.

~~2.4.~~ Telomerase measurement

Formatted: Font: 16 pt

Blood samples used for this study were collected between 2015 and 2017 at the two sites. TA was detected by real-time telomeric repeat amplification protocol (RT-TRAP) [41], with some modifications. In brief, blood samples were drawn in line with Swedish nursing standards, with the participants in a fasting and rested state (minimum 20 minutes). Whole blood was obtained in 8 ml BD Vacutainer® CPT™ Mononuclear Cell Preparation Tube - Sodium Citrate (Becton Dickinson). Thereafter, lymphocytes and monocytes were separated within two hours of sampling, according to manufacturer's protocol. Approximately half of the pelleted cells were lysed by incubation with 120 µl CHAPS (Merck Millipore, including 0.15 units/µl RiboLock [LifeTechnologies, Thermo Fisher Scientific]) on wet ice for 30 min and three short vortexes. Following this initial preparation done locally at laboratories on respective site, the samples were

labelled and frozen at -80°C, after which they were transported to Center for Molecular Medicine (CMM) at Karolinska Institutet for storage and further analysis.

Thawed lysate was centrifuged at 4°C at 12000xg for 20 min and the supernatant was transferred to an empty tube. The total protein concentration was measured in the supernatant by the DC Protein Assay (Bio-Rad) and all patients' cell lysates were diluted to equal concentrations in CHAPS (0.95 µg/µl), aliquoted and refrozen at -80°C. An aliquot per sample was thawed and equal amounts of total protein (3.8 µg) from each sample was added to a reaction mix with a total volume of 50 µL containing 1 mM of each dNTP, 20 mM Tris-HCl (pH 8.3), 2.5 mM MgCl2, 63 mM KCl, 0.05% Tween 20, 1 mM EGTA, 8 µmol of each of the primers TS (5'-AATCCGTCGAGCAGAGTT-3') and ACX (5'- GCGCGG(CTTACC)3CTAACC-3'). HepG2 (hepatic cancer) and ARO (thyroid cancer) cell lines were used as telomerase-positive controls, whereas CHAPS buffer and heat-inactivated samples were used as negative controls. TSR8 is an oligonucleotide with a sequence identical to the TS primer extended with 8 telomeric repeats being AG(GGTTAG)$_7$. Serial dilutions of TSR8 control template were used to generate a standard curve to calculate TA. The serial dilutions were 0.2 amoles/µL, 0.04 amoles/µL, 0.008 amoles/µL, 0.0016 amoles/µL, 0.00032 amoles/µL, and 0.000064 amoles/µL corresponding to 200, 40, 8, 1.6, 0.32 and 0.064 TPG units/µL; TPG is the total product generated, corresponding to the number of TS primers (1 unit equals 10^{-3} amoles or 600 molecules) that are extended with at least 3 TTAGGG repeats by telomerase in the extract in a 30 min incubation at 30°C. The reaction mix was incubated at 30°C for 30 min followed by termination at 95°C for 5 min. Then 8 µL of the telomeric repeat products (i.e. corresponding to 8/50*3.8=0.608 ug total protein) were used for the RT-TRAP assay amplified by 8 µL Power SYBR Green in 384-well plates. The reaction was performed on QuantStudio 7 Flex (Applied Biosystems; Life Technologies; Thermo Fisher Scientific Inc.) with

the following conditions: 95°C for 10 min, followed by 36 repeats of 95°C for 20 s, 52°C for 30 s and 72°C for 60 s. Samples, controls and standard curve dilutions were run in triplicate, standard curve and controls on all plates. All samples from each patient were run on the same randomized plate. Efficiency was 99-105%. The mean of the correlation coefficients of the standard curves was > 0.993. The mean coefficients of variation (CV) of intra-plate Ct values for the standard dilutions of the five plates was CV = 0.56%, and the mean inter-plate CV of three inter-plate controls was CV = 1.6%. The detection success rate was 100%. For each supernatant, aliquots are still there with original protein concentration.

~~2.5.~~ Telomere length measurement

Formatted: Font: 16 pt

The other half of the pelleted cells, collected and prepared at both sites, were also stored at -80°C until DNA extraction. Genomic DNA was extracted based on the kit [42], but the speed of centrifuge was changed to 6000g and avoid vortex by inverse, and incubate at 37°C for 3 h. DNeasy® Blood & Tissue Kit (Qiagene). DNA concentration was quantified with NanoDrop ND-1000 Spectrophotometer (Nano-Drop Technologies Inc., Wilmington, DE, USA). Relative TL was determined using real-time quantitative polymerase chain reaction (qPCR) according to Cawthon et al’s protocol [43] where the relative telomere to single copy gene (T/S) ratios was determined using a standard curve. In brief, each DNA sample (10 ng) was assessed for the telomere (*Tel*) and the single-copy gene (hemoglobin-b, *HGB*) in triplicate within the same 384-well plate, amplified by using Power SYBR Green in 10 µl total reaction volume. The reaction was performed on QuantStudio 7 Flex (Applied Biosystems; Life Technologies, Carlsbad, CA, USA) with the following conditions: 50°C for 2 min, then 95°C for 10 min, followed by 40 repeats of 95°C for 15 s and 60°C for 1 min, followed by a dissociation stage to monitor amplification specificity. The

same standard curve of pooled DNA from these patient samples ranging from 80 ng to 0.128 ng, was run on each plate for both genes and was used to determine the quantity of each gene for each sample. This allowed controlling for differences in the efficiencies between that of *Tel* and *HGB*. The gene quantities were then used to determine the T/S ratio for each sample. DNA samples with a Ct standard deviation of ≥ 0.35 between triplicates or a Ct value outside the standard curve were omitted from the analyses. The correlation coefficients of the standard curves were above 0.99 for each primer set and 384-plate. The inter-plate coefficient of variation (CV) of T/S ratio was 9.1% calculated from a patient sample run in seven 384-well plates. The TL analysis detection success rate was 100%. The primer sequences were (written 5’à3’): Tel1: CGGTTTGTTTGGGTTTGGGTTTGGGTTTGGGTTTGGGTT; Tel2: GGCTTGCCTTACCCTTACCCTTACCCTTACCCTTACCCT; *HGB* Fw: GCTTCTGACACAACTGTGTTCACTAGC; *HGB* Rv: CACCAACTTCATCCACGTTCACC. Samples from all three timepoints per person were assayed in the same 384-well plate.

~~2.6.~~ Statistical analysis

Formatted: Font: 16 pt

The software used for analysis was IBM SPSS version 26. Background data are described as means and standard deviations, or frequencies and percentages, and Chi-Square or Fishers exact tests were calculated for descriptive background measures. The TA variable was ln-transformed due to significant skewness. Partial correlations were used to assess how the TL (adjusted for age) and TA correlated with pain intensity, spread and psychological outcomes at baseline. In addition, we used an analytic approach similar to Hassett et al [5], where the groups were divided into “high pain/low pain”; “high HAD score/low HAD score”; and “high WHOQOL-SRPB score/low WHOQOL-SRPB score” (the group mean score was used as cut-off), and ANCOVA analyses

adjusted for age were conducted with TL and TA as dependent variables. A linear regression analysis was conducted, with age, anxiety and depression and pain intensity as independent variables, to study their impact on the telomere and telomerase variables, which were thus dependent variables in the analyses. A potential mediation model was conducted in an exploratory fashion following the standard mediation model of Baron and Kenny [44]. Direct effects were modeled in univariate regression models. Beta-weights, i.e. partial correlations coefficients were given for the different paths. This model was an ad-hoc decision after we saw the data pattern, because we wanted to explore the data further. It has to be viewed as a preliminary additional analysis to clarify relationships. We mark significance levels according to a p-level of .005 corrected for multiple testing, which is in accordance with a recommended level for new discoveries [45]. But due to the exploratory nature of the study, we do not emphasize significances, but rather effect size, i.e. the extent of the correlation [46].

3. RESULTSesults

3.1. Sample characteristics

The socio-demographic background variables and pain variables of participants are presented in Table 1. The majority of the participants were female, from Sweden and currently working. Most had upper secondary school or university education. Regarding pain, 82% reported it as constant and high, with an average pain intensity of 7.1 (out of 10) and a mean duration of nearly 12 years. With this amount of pain 60% were taking prescription drugs for pain and various other types of medications. Almost one third of the participants were using herbals and other supplements. In order to assess representativeness of our sample it was compared to the target population and hence

Formatted: Font: 18 pt

Formatted: Font: 16 pt

Table 2 presents essential characteristics of the sample and target population, with numbers from a report by SQRP [47]. Table 3 presents the differences in participant demographic and pain variables from the different sites, where differences in distributions and means are also presented.

Table 1. Socio-demographic background and pain variables of participants

Variable	n	Score	SD
Gender	84		
**Female**	84	85.7%	
**Male**	84	14.3%	
Age	84	47.6	10.3
Country of birth	84		
**Sweden**	84	77.4%	
**Other Nordic countries**	84	3.6%	
**Europe**	84	9.5%	
**Outside Europe**	84	9.5%	
Education	84		
**Primary schooling**	84	7.1%	
**Upper secondary school**	84	64.3%	
**University**	84	22.6%	
**Other**	84	6.0%	
Working	82	87.8%	
Blood pressure	77		
**Systolic**	77	120.4 mmHg	17.3
**Diastolic**	77	76.9 mmHg	10.8
BMI	77	27.6	10.3
ICD-10 diagnosis	84		
**M-diagnoses**	84	66.7%	
**R-diagnoses**	84	19.0%	
**Q-diagnoses**	84	8.0%	
**F-diagnoses**	84	7.1%	
**Other diagnoses**	84	2.4%	
Medication	84		
**Prescription drugs for pain**	84	61%	
**Prescription drugs for other**	84	45.2%	
**Non-prescription drugs for pain**	75	28%	
**Non-prescription drugs for other**	75	10.7%	
**Herbals**	77	26%	
Type of pain	83		
**Constant**	83	81.9%	
**Periodic**	83	18.1%	
Pain duration	81	11.8	10.5
Pain intensity NRS	83	7.1	1.5
Pain spread	83	19.1	9.0

BMI: Body Mass Index, ICD-10: International Classification of Diseases, M-diagnoses: diseases of the musculoskeletal system and connective tissue, R-diagnoses: symptoms, signs and abnormal clinical and laboratory findings not elsewhere

Formatted: English (United States)

Formatted: English (United States)

classified, Q-diagnoses: congenital malformations, deformations and chromosomal abnormalities, F-diagnoses: Mental, Behavioral and Neurodevelopmental disorders, NRS: numerical rating scale.

~~***Table 1.*** *Socio-demographic background and pain variables of participants*~~

Formatted: Font: Italic

Variable	n	Score	SD
Gender	84		
**Female**	84	85.7%	
**Male**	84	14.3%	
Age	84	47.6	10.3
Country of birth	84		
**Sweden**	84	77.4%	
**Other Nordic countries**	84	3.6%	
**Europe**	84	9.5%	
**Outside Europe**	84	9.5%	
Education	84		
**Primary schooling**	84	7.1%	
**Upper secondary school**	84	64.3%	
**University**	84	22.6%	
**Other**	84	6.0%	
Working	82	87.8%	
Blood pressure	77		
**Systolic**	77	120.4 mmHg	17.3
**Diastolic**	77	76.9 mmHg	10.8
BMI	77	27.6	10.3
ICD-10 diagnosis	84		
**M-diagnoses**	84	66.7%	
**R-diagnoses**	84	19.0%	
**Q-diagnoses**	84	8.0%	
**F-diagnoses**	84	7.1%	
**Other diagnoses**	84	2.4%	
Medication	84		
**Prescription drugs for pain**	84	61%	
**Prescription drugs for other**	84	45.2%	
**Non-prescription drugs for pain**	75	28%	
**Non-prescription drugs for other**	75	10.7%	
**Herbals**	77	26%	
Type of pain	83		
**Constant**	83	81.9%	
**Periodic**	83	18.1%	
Pain duration	81	11.8	10.5
Pain intensity NRS	83	7.1	1.5
Pain spread	83	19.1	9.0

~~BMI: Body Mass Index, ICD-10: International Classification of Diseases, M-diagnoses: diseases of the musculoskeletal system and connective tissue, R-diagnoses: symptoms, signs and abnormal clinical and laboratory findings not elsewhere classified, Q-diagnoses: congenital malformations, deformations and chromosomal abnormalities, F-diagnoses: Mental, Behavioral and Neurodevelopmental disorders, NRS: numerical rating scale.~~

Table 2. Essential sample characteristics in comparison with the target population

Variable	Sample score	Target population score*
**Age**	47	44
**Gender F/M**	86/14%	79/21%
**Working now**	88%	67%
**Nationality Sweden/other**	81/19%	82/18%
**Senior high school**	64%	41%
**University**	23%	42%
**BMI**	28	27
**Pain duration**	12 years	9 yea

Formatted Table

**Pain intensity**	7.1	r s 4 . 1
**Pain spread**	19	1 5

*Based on Swedish National Quality Registry for Pain Rehabilitation 2020

Table 3: Differences in demographic and pain variables in the two patient cohorts (Vidar Clinic and Linköping University Hospital)

	**Clinic**	**N**	**Mean**	**p-value (t-test)**
**Sex**	Vidar	51	96 (% female)	0.001 (Fisher's exact)
	Linköping	33	70 (% female)	
**age**	Vidar	51	50.50	0.001
	Linköping	33	43.17	
**Pain duration (years)**	Vidar	49	15.39	<0.001
	Linköping	32	6.38	
**Pain intensity**	Vidar	51	6.95	0.43
	Linköping	32	7.22	
**Pain spread**	Vidar	51	22.53	<0.001
	Linköping	32	13.69	

~~**Table 2.** *Essential sample characteristics in comparison with the target population*~~

~~**Variable**~~	~~**Sample score**~~	~~**Target population score***~~
~~**Age**~~	~~47~~	~~44~~
~~**Gender F/M**~~	~~86/14%~~	~~79/21%~~
~~**Working now**~~	~~88%~~	~~67%~~
~~**Nationality Sweden/other**~~	~~81/19%~~	~~82/18%~~

**Senior high school**	64%	41%
**University**	23%	42%
**BMI**	28	27
**Pain duration**	12 years	9 years
**Pain intensity**	7.1	4.1
**Pain spread**	19	15

*Based on Swedish National Quality Registry for Pain Rehabilitation 2020

Table 3: *Differences in demographic and pain variables in the two patient cohorts (Vidar Clinic and Linköping University Hospital)*

	**Clinic**	**N**	**Mean**	**p-value (t-test)**
**Sex**	Vidar	51	96 (% female)	0.001 (Fisher's exact)
	Linköping	33	70 (% female)	
**age**	Vidar	51	50.50	0.001
	Linköping	33	43.17	
**Pain duration (years)**	Vidar	49	15.39	<0.001
	Linköping	32	6.38	
**Pain intensity**	Vidar	51	6.95	0.43
	Linköping	32	7.22	
**Pain spread**	Vidar	51	22.53	<0.001
	Linköping	32	13.69	

3.2. Patient reported outcome measures

Formatted: Font: 16 pt

All PROM's were normally distributed. The PROM's for *clinical pain* including pain intensity, pain duration, pain spread, and medication are presented in table 1. All other PROM's, including subscales are presented in Table 4. To better understand the interrelatedness of different PROM's, correlations were calculated amongst all the questionnaires and pain measures and are presented in Table 5.

Table 4. Patient Reported Outcome Measures including subscales and scoring

Questionnaire	n	Items, n	Scoring	Min-max	Mean	Std. Dev
**HAD scale score**	84	14	0-3	0-42	19.17	8.49
HAD scale anxiety	84	7	0-3	0-21	10.13	5.10
HAD scale depression	84	7	0-3	0-21	9.04	4.33
**EQ5D VAS**	82	1	0-100	0-100	40.77	17.37
**EQ5D Index value**	84	5	1-3	5-15	0.26	0.32
**FFMQ score**	80	29	1-5	29-145	91.95	15.12
FFMQ non-reactivity	80	6	1-5	6-30	17.80	3.94
FFMQ observe	80	7	1-5	7-35	24.40	5.02
FFMQ acting with awareness	80	5	1-5	5-25	14.55	3.87
FFMQ describe	80	6	1-5	6-30	19.26	4.80
FFMQ non-judging	80	5	1-5	5-25	16.03	4.22
**CPAQ score**	79	8	0-6	0-48	22.14	7.18
CPAQ pain willingness	79	4	0-6	0-24	11.72	5.34
CPAQ activity engagement	79	4	0-6	0-24	10.42	4.63
**WHOQOL-SRPB score**	75	32	1-5	32-160	92.00	26.44
WHOQOL-SRPB spiritual connection	75	4	1-5	4-20	10.27	4.98
WHOQOL-SRPB meaning and purpose in life	75	4	1-5	4-20	12.85	3.49
WHOQOL-SRPB experiences of awe and wonder	75	4	1-5	4-20	10.52	4.62
WHOQOL-SRPB wholeness and integration	75	4	1-5	4-20	12.84	3.83
WHOQOL-SRPB spiritual strength	75	4	1-5	4-20	12.92	3.23
WHOQOL-SRPB inner peace	75	4	1-5	4-20	10.93	4.04
WHOQOL-SRPB hope and optimism	75	4	1-5	4-20	10.51	3.38
WHOQOL-SRPB faith	75	4	1-5	4-20	11.16	3.37

HAD: Hospital anxiety depression scale, EQ5D (VAS): Euro Quality of Life 5 Dimensions (visual analogue scale), FFMQ: Five Facets Mindfulness Questionnaire, CPAQ: Chronic Pain Acceptance Questionnaire, WHOQOL-SRPB: WHO Quality of Life –Spiritual, Religious and Personal Beliefs.

Table 5. Correlations amongst questionnaires and pain measures

	**HAD score**	**EQ5D index**	**EQ5D VAS**	**FFMQ score**	**CPAQ score**	**WHO score**	**Pain intensity**	**Pain duration**	**Pain spread**
**[illegible]**									
**EQ5D index**	-.300								
**EQ5D VAS**	-.347*	.378*							
**FFMQ score**	-.470*	.019	.019						
**CPAQ score**	-.292	.212	.366*	-.005					
**WHO score**	-.347*	.189	.236	.485*	.064				
**Pain intensity**	.292	-.333*	-.270	-.230	-.160	-.235			

Formatted: English (United States)

Formatted: English (United States)

Formatted: English (United States)

Formatted: English (United States)

Formatted: English (United States)

**Pain duration**	.011	.051	.004	.070	-.101	.166	-.108	
**Pain spread**	.298	-.104	-.158	.008	-.273	.090	.095	.330*

Abbreviations: Hospital Anxiety and Depression scale (HAD), Euroqol 5 Dimensions (EQ5D), Visual Analogue Scale (VAS), Five Facets Mindfulness Questionnaire (FFMQ), Chronic Pain Acceptance Questionnaire (CPAQ), WHO Quality of Life Spiritual, Religious and Personal Beliefs (WHO). *Correlation is significant at the 0.005 level (2-tailed)

Table 4. *Patient Reported Outcome Measures including subscales and scoring.*

Questionnaire	n	Items, n	Scoring	Min-max	Mean	Std. Dev
**HAD scale score**	84	14	0-3	0-42	19.17	8.49
HAD scale anxiety	84	7	0-3	0-21	10.13	5.10
HAD scale depression	84	7	0-3	0-21	9.04	4.33
**EQ5D VAS**	82	1	0-100	0-100	40.77	17.37
**EQ5D Index value**	84	5	1-3	5-15	0.26	0.32
**FFMQ score**	80	29	1-5	29-145	91.95	15.12
FFMQ non-reactivity	80	6	1-5	6-30	17.80	3.94
FFMQ observe	80	7	1-5	7-35	24.40	5.02
FFMQ acting with awareness	80	5	1-5	5-25	14.55	3.87
FFMQ describe	80	6	1-5	6-30	19.26	4.80
FFMQ non-judging	80	5	1-5	5-25	16.03	4.22
**CPAQ score**	79	8	0-6	0-48	22.14	7.18
CPAQ pain willingness	79	4	0-6	0-24	11.72	5.34
CPAQ activity engagement	79	4	0-6	0-24	10.42	4.63
**WHOQOL-SRPB score**	75	32	1-5	32-160	92.00	26.44
WHOQOL-SRPB spiritual connection	75	4	1-5	4-20	10.27	4.98
WHOQOL-SRPB meaning and purpose in life	75	4	1-5	4-20	12.85	3.49
WHOQOL-SRPB experiences of awe and wonder	75	4	1-5	4-20	10.52	4.62
WHOQOL-SRPB wholeness and integration	75	4	1-5	4-20	12.84	3.83
WHOQOL-SRPB spiritual strength	75	4	1-5	4-20	12.92	3.23
WHOQOL-SRPB inner peace	75	4	1-5	4-20	10.93	4.04
WHOQOL-SRPB hope and optimism	75	4	1-5	4-20	10.51	3.38
WHOQOL-SRPB faith	75	4	1-5	4-20	11.16	3.37

HAD: Hospital anxiety depression scale, EQ5D (VAS): Euro Quality of Life 5 Dimensions (visual analogue scale), FFMQ: Five Facets Mindfulness Questionnaire, CPAQ: Chronic Pain Acceptance Questionnaire, WHOQOL-SRPB: WHO Quality of Life –Spiritual, Religious and Personal Beliefs.

Table 5. *Correlations amongst questionnaires and pain measures*

	HAD score	EQ5D index	EQ5D VAS	FFMQ score	CPAQ score	WHO score	Pain intensity	Pain duration	Pain spread
**HAD score**									
**EQ5D index**	-.300								

**EQ5D VAS**	-.347*	.378*						
**FFMQ score**	-.470*	.019	.019					
**CPAQ score**	-.292	.212	.366*	-.005				
**WHO score**	-.347*	.189	.236	.485*	.064			
**Pain intensity**	.292	-.333*	-.270	-.230	-.160	-.235		
**Pain duration**	.011	.051	.004	.070	-.101	.166	-.108	
**Pain spread**	.298	-.104	-.158	.008	-.273	.090	.095	.330*

Abbreviations: Hospital Anxiety and Depression scale (HAD), Euroqol 5 Dimensions (EQ5D), Visual Analogue Scale (VAS), Five Facets Mindfulness Questionnaire (FFMQ), Chronic Pain Acceptance Questionnaire (CPAQ), WHO Quality of Life Spiritual, Religious and Personal Beliefs (WHO). *Correlation is significant at the 0.005 level (2-tailed)

3.3. Cellular measurements

Formatted: Font: 16 pt

The mean TL of the group was 1.163 (n=84, SD=0.412) and the group mean TA (ln transformed) was 2.064 TPG units/µL (n=80, SD=1.774). At group level, all routine blood test results were within normal reference intervals according to Swedish standards at Karolinska University Laboratory. (data not shown).

3.4. Telomere measurement correlations and associations with PROMs

Formatted: Font: 16 pt

TL was negatively associated with age (r=-.210, p=.055) and in an age-adjusted correlation, only P-cholesterol (r=.225, p=.041) and LDL-cholesterol (r=.218, p=.050) correlated with TL. . No correlations were found between measures in blood samples and the TA. (data not shown). With self-report measures, a negative correlation was found between the TL and HAD score (r = -.219, p = .047) (Table 6). Also, a positive correlation was seen between the TL and WHOQOL-SRPB (r

= .224, p = .052) (Table 6). Interestingly the TL was not correlated to any of the pain measures. The TA did not correlate with any of the self-reported questionnaires or pain measures. When using an analytic approach similar to Hassett et al [5], where the groups were divided into "high pain/low pain" and "high HAD score/low HAD score" (the group mean score was used as cut-off), and ANCOVA analyses adjusted for age were conducted with TL and TA as dependent variables, no associations were detected (for TA and HAD: Eta^2 = 0.00; p = 0.901;, for TL and HAD: Eta^2 = 0.012; p = 0.32; for TA and pain: Eta^2 = 0.011, p = 0.357; for TL and pain: Eta^2 = 0.001; p = 0.78; for TA and WHOQOL-SRPB: Eta^2 = 0,008; p = 0,460; for TL and WHOQOL-SRPB: Eta^2 = 0,022; p = 0,225).

Formatted: Superscript

Formatted: Superscript

Table 6. Partial correlations with p-values, between TA/TL and self-report measures

	HAD	EQ5D index	EQ5D VAS	FFMQ	CPAQ	WHO	PI	PD	PS
TL	-.219 (.047)	-.003 (.977)	.141 (.211)	.131 (.250)	.189 (.089)	.224 (.052)	-.154 (.166)	.083 (.462)	-.188 (.090)
TA	-.042 (.709)	-.080 (.480)	-.100 (.383)	.039 (.738)	-.031 (.787)	.145 (.225)	-.177 (.119)	.181 (.113)	.222 (.049)

Formatted: English (United States)

Abbreviations: Hospital Anxiety and Depression scale (HAD), Euroqol 5 Dimensions (EQ5D), Visual Analogue Scale (VAS), Five Facets Mindfulness Questionnaire (FFMQ), Chronic Pain Acceptance Questionnaire (CPAQ), WHO Quality of Life Spiritual, Religious and Personal Beliefs (WHO), Pain Intensity (PI), Pain Duration (PD), Pain Spread (PS), Telomere Length (TL), Telomerase Activity (TA).

Table 6. *Partial correlations with p-values, between TA/TL and self-report measures*

	HAD	EQ5D index	EQ5D VAS	FFMQ	CPAQ	WHO	PI	PD	PS
TL	-.219 (.047)	-.003 (.977)	.141 (.211)	.131 (.250)	.189 (.089)	.224 (.052)	-.154 (.166)	.083 (.462)	-.188 (.090)
TA	-.042 (.709)	-.080 (.480)	-.100 (.383)	.039 (.738)	-.031 (.787)	.145 (.225)	-.177 (.119)	.181 (.113)	.222 (.049)

Abbreviations: Hospital Anxiety and Depression scale (HAD), Euroqol 5 Dimensions (EQ5D), Visual Analogue Scale (VAS), Five Facets Mindfulness Questionnaire (FFMQ), Chronic Pain Acceptance

~~Questionnaire (CPAQ), WHO Quality of Life Spiritual, Religious and Personal Beliefs (WHO), Pain Intensity (PI), Pain Duration (PD), Pain Spread (PS), Telomere Length (TL), Telomerase Activity (TA).~~

~~3.5.~~ Mediation effects

Formatted: Font: 16 pt

As the first order correlations and theoretical consideration suggested that the relationship between spiritual well-being (WHOQOL-SRPB) and TL might be mediated by the effect of the former on depression and anxiety (HAD scale) we inspected a potential mediation model in an exploratory fashion. First, the direct effects a, b and c were modeled in univariate regression models. Beta-weights, i.e. partial correlations coefficients are given for the different paths in Figure 1. Modelling the total effect using the mediation path, the interaction a*b (Total Effect = c + a*b), following the standard mediation model of Baron and Kenny [44] yields a total effect (beta = 0.008; p = .045), i.e. supports the idea of a mediation of the direct effect of spiritual well-being on TL by anxiety and depression (HAD scale). However, the indirect effect, the interaction a*b (ab = 0.002; beta = -.23; p = .15) is not significant.

Insert Figure 1 here

Figure 1. Mediation model

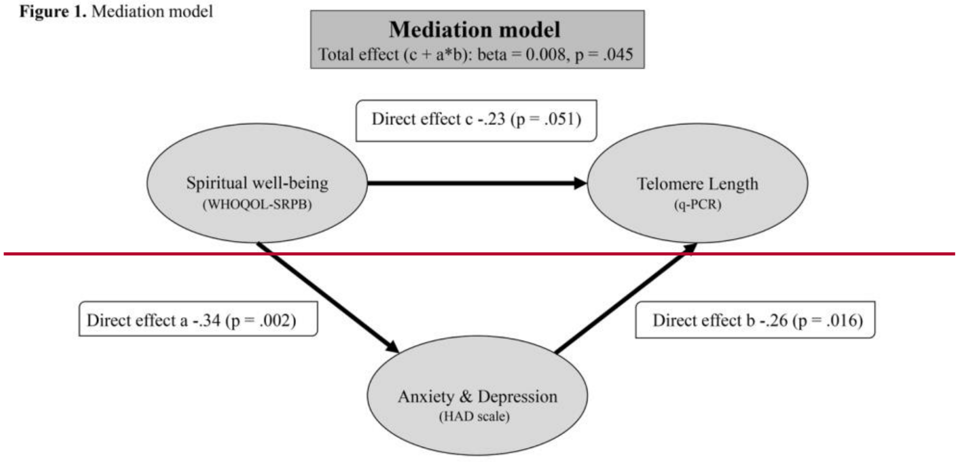

WHOQOL-SRPB: WHO Quality of Life –Spiritual, Religious and Personal Beliefs, HAD scale: Hospital Anxiety and Depression Scale; q-PCR: quantitative Polymerase Chain Reaction.

~~4. DISCUSSION~~ Discussion

Formatted: Font: 18 pt

To the best of our knowledge this is the first scientific study that explores the body-mind-spirit perspective of pain in an integrated and comprehensive manner, by investigating both telomeres, blood tests and questionnaires covering pain, coping, depression, mindfulness and spiritual well-being, including personal and religious beliefs.

Our typical study participant was a woman that rated her overall health poorly, with EQ5D VAS mean being 41 out of a maximum of 100. In comparison, the Swedish population norm (not standardized) is 83 out of 100 [48]. The EQ5D index value is 0.85 for the Swedish general population, whereas our participant mean score was 0.26 (SD 0.32). Our average participant had a clinically probable risk for both anxiety and depression with a moderate pain coping capacity - more for pain –willingness than for activity engagement. When aspects of mindfulness were

reported, our average participant was struggling with acting with awareness or staying focused on the task at hand without becoming distracted. Mindfulness meditation could be beneficial as an intervention since it can improve pain, depression symptoms and quality of life with no risk of addiction [49]. On average, participants scored moderately on all facets of the WHOQOL-SRPB. Their sense of meaning and purpose in life together with a feeling of wholeness and integration was slightly more prominent while there was a lack of hope and optimism as well as few experiences of awe and wonder. Despite the obvious challenges to realize a universal instrument for spirituality that allow equitable comparison among people with different worldviews [50] the SRPB dimensions used in our study can be seen as related to spiritual quality of life in one way or another. Studies have repeatedly established positive links between ~~religion,~~ spirituality, religion and health where for example religious involvement is correlated with better mental health in the areas of depression, substance abuse, and suicide [51].~~(reference).~~ It has also been shown that cardiovascular and cortisol stress responses of a sample of university students can be predicted by [illegible] secularity, religiosity, spirituality, and existential search [52]~~(reference)~~. Since spirituality seem to enhance quality of life and affective well-being in chronic pain patients, partly mediated through coping [53~~1~~-55~~3~~], health care interventions including spiritual, religious and personal meaning dimensions might be beneficial.

Commented [BTF1]: Bonelli, R.M., Koenig, H.G. Mental Disorders, Religion and Spirituality 1990 to 2010: A Systematic Evidence-Based Review. *J Relig Health* **52**, 657–673 (2013). https://doi.org/10.1007/s10943-013-9691-4

Commented [BTF2]: Schnell, T., Fuchs, D. & Hefti, R. Worldview Under Stress: Preliminary Findings on Cardiovascular and Cortisol Stress Responses Predicted by Secularity, Religiosity, Spirituality, and Existential Search. *J Relig Health* **59**, 2969–2989 (2020). https://doi.org/10.1007/s10943-020-01008-5

Interestingly, the blood samples did not deviate from normal reference intervals ~~(data not shown)~~ indicating that patients with severe and long-lasting pain paradoxically appear “perfectly healthy”. However, more complex proteomics or pro-inflammatory markers have been shown to deviate in chronic pain patients and correlate with other health aspects such as HAD scores [56~~4~~].

Important to mention is the unbalanced gender ratio (86-14) in this sample, characteristic of the pain patient population nationally as well as globally, illuminating a gender issue. Women

are noticeably overrepresented amongst patients living with chronic pain [57~~5~~-59~~7~~] and it has been highlighted as a gendered condition possibly subjected to medical gender bias [58~~6~~]. Further adding to this, a report from 2018 requested by the Swedish Government concluded that there were close to zero (1 of 185) reviews with focus on women in this female dominated condition [59~~7~~].

Regarding correlations, the participants' self-reported anxiety and depression had stronger relationships with measures of mindfulness and spiritual well-being than with pain measures.~~:~~ Indeed, our findings indicate that stronger~~ personal ,~~ spiritual, ~~or~~ religious or personal beliefs may be protective of depression and anxiety, and that this may perhaps be mediated by increased mindfulness - presence in everyday life. However, our study design does not allow us to assess the direction of association, whereby it may also be so that decreased depression and anxiety instead enables increased mindfulness and~~ personal,~~ spiritual, ~~and~~ religious and personal beliefs.

One interesting finding in this study is that the WHOQOL-SRPB had a positive correlation to TL, meaning that a greater sense of spiritual well-being, meaning, purpose and wholeness might be linked with longer telomeres. Furthermore, our exploratory model supports the idea of a mediation of the direct effect of spiritual well-being on TL by anxiety and depression as measured by the HAD scale. The relationship between spiritual well-being and TL could indicate a weak relationship between PROM's and aspects of telomeres. As we had only a small sample, the correlations, though interesting from a conceptual point of view, did not reach conventional significance thresholds. This likely indicates a power problem which underlines the need for replication with a larger data set. The HAD scale was negatively correlated with the TL, meaning that higher depression/anxiety scores, could be linked to shorter telomeres. Similar findings have been described by Hassett et al [5] and Sibille et al [26].

Another finding is the absence of correlation between TL and the pain measures selected in this study. This is surprising since others have found statistically significant correlations between TL and pain measures [5, 21-24]. In contrast, TA had a positive relationship with pain spread, i.e. high TA was associated with higher pain spread. But again, this correlation is not large enough to reach a convincing significance level with the sample size we had available. This is ambiguous since TL is negatively associated with pain spread to a notable degree. Surprisingly, when repeating the cross table statistical analysis of Hassett et al [5] by grouping high pain-high depression in contrast to low pain-low depression we were still unable to reproduce the results with correlations between TL and high depression - high pain. ~~(data not shown).~~ For all lab tests, pain measures and other self-report measures it is the HAD scale and the WHOQOL-SRPB that emerge in correlation with TL.

In summary, what ultimately correlates with the genomic integrity within this sample of chronic pain patients is neither quantitative pain measures or scales, nor index numbers generated for health economy calculation purposes, but instead anxiety, depression and ~~personal,~~ spiritual, ~~and~~ religious and personal beliefs. And those, in turn, have a significant relation to both the single EQ5D VAS question and some of the self-report pain measures. While how patients feel is often dismissed in health care [8, 9], it seems to be mirrored by the state of genomic integrity as well as in self-reports in our study. This is in line with the model proposed by Wideman et al [10], with patients as experts on their own condition.

Although the recommendations by large organizations like IMMPACT is to use NRS as the core measurement for clinical pain [29], it doesn’t relate to the genomic integrity of the participants in this study. And while measures of emotional functioning are included in the recommendations

for core outcome measures, the spiritual dimension of pain is absent from the international recommendations [29].

The global opioid crisis is a painful reality that is of foremost relevance to pain management. Opioids are commonly prescribed to chronic pain patients in spite of doubtful effectiveness as long-term treatment. Between 1999-2014 approximately 165 000 persons died of overdose related to opioid medications - only in the United States [60~~58~~]. Moreover, during 2015 opioids were responsible for a third of the almost half a million global drug deaths [61~~59~~]. And despite the relation to global morbidity, mortality and doubtful long-term effectiveness, 24 % of the participants in our sample of chronic pain patients had opioids prescribed for their pain. Our study gives reasons to suggest that it might be beneficial for health care to be guided by interventions also including the dimensions of emotional well-being and spiritual, religious and personal ~~dimensions of pain, as well as personal and religious meaning.~~beliefs when it comes to pain management, i.e. ~~.~~ a~~A~~ focus on promoting connection at the expense of addiction.

Formatted: Font color: Red

Increasingly, more patients are offered rehabilitation in so-called multimodal programs with psychological and physical activities and this biopsychosocial approach and model in pain management is currently recommended in Sweden [59~~7~~, 62~~0~~] , though without reference to spiritual aspects of health and pain. Given that TL is a good indicator for longevity and robustness of health [12] and that spiritual well-being is somewhat associated with TL this indicates a novel arena with potential in pain rehabilitation. Our findings are preliminary and warrant further scrutiny.

~~4.1. Methodological considerations and ~~L~~l~~imitations

Formatted: Font: 18 pt

A limitation with this study is its cross-sectional design with the innate inability to draw causal conclusions, and its comparatively small sample size. The latter was due to organisational restrictions that could not be remedied. However, the goal was rather to generate some preliminary insights, questions and hypotheses for future studies. Another apparent limitation is the lack of a control group. This is part of the nature of this study as patients cannot be randomised to become chronic pain patients. Only naturalistic cohorts of healthy, age matched controls would be possible, and we were unable to recruit such controls for this study for organisational reasons.

There are several biases in this project. Firstly, the two sites had different acceptance rates which can generate bias. Together, 45% of all eligible persons accepted participation which increases the risk of bias and raises the concern of the sample not being representative of the target population. Yet, by comparing our data to national health registry data [47] we can see that our sample was in fact representative of the target population, as well as assessing on what accounts they were not.

The double data entry and cross-checking and careful validation of the database before analysis improved methodological strength in this study. Also, the inclusion of two geographically separate locations as study sites with the aim of improving generalizability can be seen as a strength. In addition, since Complementary and Alternative Medicine (CAM) modalities are widely used by pain patients [63~~1~~], the inclusion of The Vidar Clinic as a formal health care provider including certain CAM therapies can also been seen as a strength contributing to generalizability.

Another limitation is that other potential confounders apart from BMI, age or sex may have accounted for some of the results. We did not investigate the effect of tobacco or alcohol use, which are known to confound telomere measurements. Indeed, levels of exercise and nutrition and patients' medication should also be considered. Also, the initial preparation of the blood samples

used for TL and TA measurement were done by different staff at the two sites. However, the standard protocol was followed and when conducting analyses of the mononuclear cells in laboratory environment, the quality of blood sampling and initial preparation could be validated. Additionally, the randomisation of the telomere samples as well as blinding of laboratory personnel performing the tests added to methodological strengths and reduced bias.

The size of the correlations we found were rather small, between r = .2 and .3. Because of the small sample size and the comparatively large number of correlations, inherent in an exploratory project such as this one, we would not want to draw any definitive conclusions. But it seems to be an interesting hypothesis to follow up in a subsequent study whether the positive association between TL and spiritual well-being can be substantiated, as well as the negative association between depression and anxiety and TL. Also the positive correlation between TA and pain spread warrants further scrutiny. The former would be indicative of a protective effect of spirituality and meaning-making against physical disintegration. The latter would point to an association between repair processes and the spreading of pain. With such focussed hypotheses further studies could produce more confirmatory evidence than our data can provide.

Also, it should be noted that our mediation model is intuitive, but far from definitive. That spiritual well-being together with an interaction between SRPB and HAD should have a positive, albeit small, influence on telomere length certainly warrants further scrutiny. As our data were cross-sectional, it would be mandatory to have longitudinal data which would lend itself to a clear causal analysis, which our analysis cannot provide. Furthermore, one can argue that mediation is only demonstrated according to the original Baron and Kenny approach, but not according to more recent discussions [64, 65], which would require an independent interaction term to be significant.

However, we nevertheless think that this potential mediation might be interesting for other researchers to scrutinize in more detail.

~~5.~~ Conclusion~~CONCLUSION~~

Formatted: Font: 18 pt

In summary, chronic pain patients participating in this study confirm the view of a complex and holistic web of health dimensions that are affected by and associated with their suffering and a heavy burden of symptoms. Our findings show that telomeres tend to be shorter in more anxious and depressed pain patients, but not in patients with more self-reported pain. Intriguingly, spiritual well-being as measured with WHOQOL-SRPB, was in this sample of patients correlated with TL. TA was more pronounced in patients reporting higher pain spread. These findings are preliminary and need confirmation. This study confirms the notion that numerical estimations of pain may be too crude to capture the complexity of chronic pain, and the widespread use of such measures in health care and research can possibly be explained by an historical tendency to assess the effectiveness of pharmacological pain management. Considering the international opioid crisis, non-pharmacological and non-opioid treatments emerge as a way forward. We have indicated some reason to believe that the emotional and spiritual dimensions of pain and health is a neglected ~~but~~ yet ~~still~~ highly important aspect for the well-being and longevity of chronic pain patients and hence should ~~also~~ be an additional focus in pain management and most importantly for further research.

Authors contributions

Formatted: Font: 18 pt

Linn Rönne Petersén: Investigation, Validation, Visualization, Data Curation, Writing – original draft, Project administration. **Maria Niemi**: Methodology, Data Curation, Formal analysis, Writing – Review and Editing. **Harald Walach**: Formal analysis, Validation, Writing – Review and Editing. **Catharina Lavebratt**: Investigation, Resources, Methodology, Writing – Review and Editing. **Liu L.,Yang**: Investigation, Writing – Review and Editing. **Björn Gerdle**: Conceptualization, Methodology, Resources, Writing – Review and Editing, Funding acquisition **Bijar Ghafouri**: Investigation, Writing – Review and Editing. **Torkel Falkenberg**: Conceptualization, Supervision, Methodology, Resources, Writing – Review and Editing, Visualization, Project administration, Data Curation, Funding acquisition.

Financial support

Formatted: Font: 18 pt

This work was supported by Ekhagastiftelsen, KI Research School in Health Care Sciences, Gyllenbergsstiftelsen, Integrative Care Science Center, Vidarstiftelsen and Mahle stiftung; making the research possible without any demands of retribution.

Formatted: No Spacing, Left

Declaration of Competing Interest

Formatted: Font: 18 pt

Declarations of interest: none

Acknowledgements

Formatted: Font: 18 pt

The many anonymous patients are deeply appreciated for generously giving of body, mind and spirit, making this project possible. Also, the following persons are gratefully acknowledged: Annele Claeson for her general support and efforts during data entry, Eva-Britt Lind and Ulrika Wentzel Olausson for essential contribution in recruitment, data collection, and data entry. Vincent Millisher for substantial contribution in analysing telomere data, as well as editing and reviewing. Tobias Sundberg for contribution to database development and initial crude analysis. Sebastian Sauer for help with evaluating the mediation model. Maria Arman for insights and expertise on nursing and Johanna Hök Nordberg for integrated feedback on manuscript and both for thoughtful supervision. Linköping Smärtcentrum and The Vidar Clinic are acknowledged for smooth collaboration. We are also grateful for initial advice and guidance on genomic aspects of the project by Elizabeth Blackburn. Lastly a thank you to the unnamed reviewers who helped elevate this work.

Formatted: Font: 18 pt

Data availability statement

There are ethical restrictions on sharing our de-identified data set publicly since data contain potentially identifying sensitive patient information. The reasons are both the rules on confidentiality, primarily to protect the research subjects, and the GDPR regulations in Sweden. ~~Data can be obtained from the authors on request~~

639

~~REFERENCES~~References

[1] ~~R.M.~~ Leadley RM, ~~N.~~ Armstrong N, ~~Y.C.~~ Lee YC, ~~A.~~ Allen A, ~~J.~~ Kleijnen J.~~,~~ Chronic diseases in the European Union: the prevalence and health cost implications of chronic pain. J Pain Palliat Care Pharmacother. 26(4) (2012) 310-25. doi: 10.3109/15360288.2012.736933.

[2] ~~S.P~~ Bergman SP, ~~P.~~ Herrstrom P, ~~K.~~ Hogstrom K, ~~I.F~~ Peterson IF, ~~B.~~ Svensson B, ~~L.T.~~ Jacobsson LT.~~,~~ Chronic musculoskeletal pain, prevalence rates, and sociodemographic associations in a Swedish population study, J. Rheumatol, 28(6) (2001) 1369-77.

[3] ~~J.~~ McBeth J, ~~D.P.~~ Symmons DP, ~~A.J.~~ Silman AJ, ~~T.~~ Allison T, ~~R.~~ Webb R, ~~T.~~ Brammah T, et al. ~~G.J. Macfarlane,~~ Musculoskeletal pain is associated with a long-term increased risk of cancer and cardiovascular-related mortality. Rheumatology (Oxford), 48(1) (2009) 74-7. doi: 10.1093/rheumatology/ken424.

[4] ~~F.~~ Wolfe W, ~~A.L.~~ Hassett AL, ~~B.~~ Walitt B, ~~K.~~ Michaud K.~~,~~ Mortality in fibromyalgia: a study of 8,186 patients over thirty-five years. Arthritis Care Res (Hoboken). 63(1) (2011) 94-101. doi: 10.1002/acr.20301

[5] ~~A.L.~~ Hassett AL, ~~, E.~~ Epel E, ~~D.J.~~ Clauw DJ, ~~R.E.~~ Haris RE, ~~S.E.~~ Harte SE, ~~A.~~ Kairys A, et al ~~S. Buyske, D.A. Williams~~. Pain is associated with short leukocyte telomere length in women with fibromyalgia. J Pain. **13**(10) (2012) 959-69. doi: 10.1016/j.jpain.2012.07.003.

[6] ~~B.~~ Gerdle B, ~~J.~~ Bjork J, ~~C.~~ Henriksson C, ~~A.~~ Bengtsson A.~~,~~ Prevalence of current and chronic pain and their influences upon work and healthcare-seeking: a population study. J Rheumatol. 31(7) (2004) 1399-406.

[7] ~~K.J.~~ Reid KJ, ~~J.~~ Harker J, ~~M.M.~~ Bala MM, ~~C.~~ Truyers C, ~~E.~~ Kellen E, ~~G.E.~~ Bekkering GE, et al., J. ~~Kleijnen,~~ Epidemiology of chronic non-cancer pain in Europe: narrative review of prevalence, pain treatments and pain impact. Current Medical Research and Opinion. **27**(2) (2011) 449-462. doi: 10.1185/03007995.2010.545813. Epub 2011 Jan 3.

[8] ~~L.~~ De Ruddere L, and ~~K.D.~~ Craig KD, Understanding stigma and chronic pain: a-state-of-the-art review. Pain. **157**(8) (2016) 1607-10. doi: 10.1097/j.pain.0000000000000512.

[9] ~~F.~~ Toye F, ~~K.~~ Seers K, ~~N.~~ Allcock N, ~~M.~~ Briggs M, Ca~~E. CA~~rr E, ~~J.~~ AndrewsJ, et al. ~~, K. Barker,~~ Patients' experiences of chronic non-malignant musculoskeletal pain: a qualitative systematic review. Br J Gen Pract. **63**(617) (2013) 829-41.

[10] ~~T.H.~~ Wideman TH, ~~R.R.~~ Edwards RR, ~~D.M.~~ Walton DM, ~~M.O.~~ Martel MO, ~~A.~~ Hudon A, ~~D.A.~~ Seminowicz DA. The Multimodal Assessment Model of Pain: A Novel Framework for Further Integrating the Subjective Pain Experience Within Research and Practice. Clin J Pain. 35(3) (2019) 212-221. doi: 10.1097/AJP.0000000000000670.

Formatted: Font: 18 pt

Formatted: Swedish (Sweden)

[11] ~~E.~~ Blackburn E, ~~E.~~ Epel E, ~~and J.~~ Lin J.~~,~~ Human telomere biology: A contributory and interactive factor in aging, disease risks, and protection. Science. 350(6265) (2015) 1193-8. doi: 10.1126/science.aab3389.

[12] ~~E.S.~~ Epel E, ~~E.H.~~ Blackburn E, ~~J.~~ Lin J, ~~F.S.~~ Dhabhar FS, ~~N.E.~~ Adler NE, ~~J.D.~~ Morrow JD, et al. ~~R.M. Cawthon,~~ Accelerated telomere shortening in response to life stress. Proc Natl Acad Sci U S A. 101(49) (2004) 17312-5. doi: 10.1073/pnas.0407162101. Epub 2004 Dec 1.

[13] ~~E.S.~~ Epel E, ~~J.~~ Lin J, ~~F.H.~~ Wilhelm FH, ~~O.M.~~ Wolkowitz OM, ~~R.~~ Cawthon R, ~~N.E.~~ Adler NE, et al.~~C. Dolbier, W.B. Mendes, E.H. Blackburn.~~ Cell aging in relation to stress arousal and cardiovascular disease risk factors. Psychoneuroendocrinology. 31(3) (2006) 277-87. doi: 10.1016/j.psyneuen.2005.08.011.

[14] ~~P.C.~~ Haycock PC, ~~E.E.~~ Heydon EE, ~~S.~~ Kaptoge S, ~~A.S.~~ Butterworth AS, ~~A.~~ Thompson A, ~~P.~~ Willeit P, Leucocyte telomere length and risk of cardiovascular disease: systematic review and meta-analysis. Bmj. 349 (2014) 4227. doi: https://doi.org/10.1136/bmj.g4227

[15] K.T. Sibille, ~~L.~~ Witek-Janusek L, ~~H.L.~~ Mathews HL, ~~R.B.~~ Fillingim RB.~~,~~ Telomeres and epigenetics: potential relevance to chronic pain. Pain. 153(9) (2012) 1789-93. doi: 10.1016/j.pain.2012.06.003.

[16] ~~E.~~ Blackburn E, ~~and E.~~ Epel E.~~,~~ The telomere effect : a revolutionary approach to living younger, healthier, longer. Orion spring, London, 2017.

[17] ~~A.~~Vaiserman A, ~~and D.~~ Krasnienkov D.~~,~~ Telomere Length as a Marker of Biological Age: State-of-the-Art, Open Issues, and Future Perspectives. Front. Genet. 11:630186 (2021) doi: 10.3389/fgene.2020.630186.

[18] ~~E.S.~~ Epel E, ~~J.~~ Lin J, ~~F.S.~~ Dhabhar FS, ~~O.M.~~ Wolkowitz OM, ~~E.~~ Puterman E, ~~L.~~ Karan L, et al. ~~E.H. Blackburn,~~ Dynamics of telomerase activity in response to acute psychological stress. Brain Behav Immun, 24(4) 2010 531-9. doi: 10.1016/j.bbi.2009.11.018.

[19] ~~D.A.~~ Skvortsov DA, ~~M.E.~~ Zvereva ME, ~~O.V.~~ Shpanchenko OV, ~~O.A.~~ Dontsova OA.~~,~~ Assays for detection of telomerase activity. Acta Naturae. 3(1) (2011) 48-68.

[20] ~~J.~~ Daubenmier J, ~~J.~~ Lin J, ~~E.~~ Blackburn E, ~~F.M.~~ Hecht FM, ~~J.~~ Kristeller J, ~~N.~~ Maninger N, et al. ~~M. Kuwata, P. Bacchetti, P.J. Havel, E. Epel,~~ Changes in stress, eating, and metabolic factors are related to changes in telomerase activity in a randomized mindfulness intervention pilot study. Psychoneuroendocrinology. 37(7) (2012) 917-28.

[21] ~~C.L.~~ Le Maitre CL, ~~A.J.~~ Freemont AJ, ~~and J.A.~~ Hoyland JA.~~,~~ Accelerated cellular senescence in degenerate intervertebral discs: a possible role in the pathogenesis of intervertebral disc degeneration. Arthritis Res Ther. 9(3) (2007) R45. doi: 10.1186/ar2198.

Formatted: English (United States)

Formatted: Swedish (Sweden)

[22] ~~S.~~ Dechsupa S, ~~W.~~ Yingsakmongkol W, ~~W.~~ Limthongkul W, ~~W.~~ Singhatanadgige W, ~~S.~~ Honsawek S.~~,~~ Relative telomere length and oxidative DNA damage in hypertrophic ligamentum flavum of lumbar spinal stenosis. PeerJ. 6: (2018) e5381. doi: 10.7717/peerj.5381

[23] ~~T.~~ Maeda T, ~~Y.~~ Kudo Y, ~~T.~~ Horiuchi T, ~~N.~~ Makino N.~~,~~ Clinical and anti-aging effect of mud-bathing therapy for patients with fibromyalgia. Mol Cell Biochem. 444(1-2) (2018) 87-92. doi: 10.1007/s11010-017-3233-4.

[24] ~~K.T.~~ Sibille KT, ~~H.~~ Chen H, ~~E.J.~~ Bartley EJ, ~~J.~~ Riley J, ~~T.L.~~ Glover TL, ~~C.D.~~ King CD, et al. ~~, H. Zhang, Y. Cruz-Almeida, B.R. Goodin, A. Sotolongo, M.E. Petrov, M. Herbert, H.W. Bulls, J.C. Edberg, R. Staud, D. Redden, L.A Bradley, R.B. Fillingim,~~ Accelerated aging in adults with knee osteoarthritis pain: consideration for frequency, intensity, time, and total pain sites. Pain Rep. 2(3) (2017) e591. doi: 10.1097/PR9.0000000000000591.

[25] ~~A.M.~~ Steward AM, ~~J.D.~~ Morgan JD, ~~J.P.~~ Espinosa JP, ~~D.C.~~ Turk DC, ~~K.V.~~ Patel KV.~~,~~ Chronic Pain and Telomere Length in Community-Dwelling Adults: Findings From the 1999 to 2002 National Health and Nutrition Examination Survey. J Pain. 18(12) (2017) 1517-1525. doi: 10.1016/j.jpain.2017.08.006.

[26] ~~K.T.~~ Sibille KT, ~~T.~~ Langaee T, ~~B.~~ Burkley B, ~~Y.~~ Gong Y, ~~T.L.~~ Glover TL, ~~C.~~ King C, et al. ~~, J.L. Riley, C. Leeuwenburgh, R. Staud, L.A. Bradley, R.B. Fillingim,~~ Chronic pain, perceived stress, and cellular aging: an exploratory study. Mol Pain. 8 (2012) 12. doi: 10.1186/1744-8069-8-12.

[27] ~~G,~~ Lucchetti G.~~,~~ *Spirituality, Religiousness and Health [Elektronisk resurs]*. 2019: Springer International Publishing.

[28] ~~G.~~ Lucchetti G, ~~H.G.~~ Koenig HG, ~~A.L.G.~~ Lucchetti ALG.~~,~~ Spirituality, religiousness, and mental health: A review of the current scientific evidence. World J Clin Cases. 16;9(26) (2021) 7620-7631. doi: 10.12998/wjcc.v9.i26.7620.

[29] ~~R.H.~~ Dworkin RH, ~~D.C.~~ Turk DC, ~~J.T.~~ Farrar JT, ~~J.A.~~ Haythornthwaite JA, ~~M.P.~~ Jensen MP, ~~N.P.~~ Katz NP, et al. ~~, R.D. Kerns, G.Stucki, R.R. Allen, N. Bellamy, D.B. Carr, J. Chandler, P. Cowan, R. Dionne, B.S. Galer, S. Hertz, A.R. Jadad, L.D. Kramer, D.C. Manning, S. Martin, C.G. McCormick, M.P McDermot, P. McGrath, S. Quessy, B.A. Rappaport, W. Robbins, J.P. Robinson, M. Rothman, M.A. Royal, L. Simon, J.W. Stauffer, W. Stein, J. Tollett, J. Wernicke, J. Witter,~~ Core outcome measures for chronic pain clinical trials: IMMPACT recommendations. Pain. 113(1-2) (2005) 9-19. doi: 10.1016/j.pain.2004.09.012.

[30] ~~L.M.~~ McCracken LM, ~~K.E.~~ Vowles KE, and ~~C.~~ Eccleston C.~~,~~ Acceptance of chronic pain: component analysis and a revised assessment method. Pain. 107(1-2) (2004)159-66. doi: 10.1016/j.pain.2003.10.012.

[31] ~~G.S.~~ Rovner GS, ~~K.~~ Arestedt K, ~~B.~~ Gerdle B, ~~B.~~ Börsbo B, ~~L.M.~~ McCracken LM.~~,~~ Psychometric properties of the 8-item Chronic Pain Acceptance Questionnaire *(CPAQ-8) in a Swedish chronic pain cohort.* J Rehabil Med. 46(1) (2014) 73-80. doi: 10.2340/16501977-1227.

Formatted: Swedish (Sweden)

[32] ~~S.J.~~ Olsson SJ, ~~O.~~ Ekblom O, ~~E.~~ Andersson E, ~~M.~~ Börjesson M, ~~L.V.~~ Kallings LV.~~,~~ Categorical answer modes provide superior validity to open answers when asking for level of physical activity: A cross-sectional study. Scand J Public Health. 44(1) (2016) 70-6. doi: 10.1177/1403494815602830.

[33] EuroQol-Group, EuroQol--a new facility for the measurement of health-related quality of life. Health Policy. 16(3) (1990) 199-208. doi: 10.1016/0168-8510(90)90421-9.

[34] ~~I.~~ Bjelland I, ~~A.A.~~ Dahl AA, ~~T.T.~~ Haug TT, ~~D.~~ Neckelman D.~~,~~ The validity of the Hospital Anxiety and Depression Scale. An updated literature review. J Psychosom Res. 52(2) (2002) 69-77. doi: 10.1016/s0022-3999(01)00296-3.

[35] ~~J.~~ Lisspers J, ~~A.~~ Nygren A, and ~~E.~~ Söderman E.~~,~~ Hospital Anxiety and Depression Scale (HAD): some psychometric data for a Swedish sample. 96(4) (1997) 281-286. doi: 10.1111/j.1600-0447.1997.tb10164.x.

Formatted: Swedish (Sweden)

[36] ~~R.~~ LoMartire R, ~~B.O.~~ Äng BO, ~~B.~~ Gerdle B, ~~L.~~ Vixner L.~~,~~ Psychometric properties of Short Form-36 Health Survey, EuroQol 5-dimensions, and Hospital Anxiety and Depression Scale in patients with chronic pain. Pain. 161(1) (2020) 83-95. doi: 10.1097/j.pain.0000000000001700.

[37] ~~S.M.~~ Skevington SM, ~~K.S.~~ Gunson KS, ~~and K.A.~~ O'Connell KA.~~,~~ Introducing the WHOQOL-SRPB BREF: developing a short-form instrument for assessing spiritual, religious and personal beliefs within quality of life. Qual Life Res. 22(5) (2013) 1073-83. doi: 10.1007/s11136-012-0237-0.

[38] ~~C.~~ Melder C.~~,~~ Vilsenhetens epidemiologi -en religionspsykologisk studie i existentiell folkhälsa [The epidemiology of lost meaning − A study in psychology of religion and existential public health in a Swedish context], in Acta Universitatis Upsaliensis, Psychologia et Sociologia Religionum 25th [Doctoral dissertation] 2011, Uppsala University Uppsala, Sweden. p. 305.

[39] ~~R.A.~~ Baer RA, ~~G.T.~~ Smith GT, ~~J.~~ Hopkins J, ~~J.~~ Krietemeyer J, ~~L.~~ Toney L.~~,~~ Using self-report assessment methods to explore facets of mindfulness. Assessment. 13(1) (2006) 27-45. doi: 10.1177/1073191105283504.

Formatted: Swedish (Sweden)

[40] ~~J.L.~~ Lilja JL, ~~A.~~ Frodi-Lundgren A, ~~J.J.~~ Hanse JJ, ~~T.~~ Josefsson T, ~~L.G.~~ Lundh LG, ~~C.~~ Sköld C, et al.~~, E. Hansen, A.G. Broberg,~~ Five Facets Mindfulness Questionnaire--reliability and factor structure: a Swedish version. Cogn Behav Ther. 40(4) (2011) 291-303.

[41] ~~M.~~ Hou M, ~~D.~~ Xu D, ~~M.~~ Bjorkholm M, ~~A.~~ Gruber A.~~,~~ Real-time quantitative telomeric repeat amplification protocol assay for the detection of telomerase activity. Clin Chem. 47(3) (2001) 519-24.

[42] ~~N.J.~~ O'Callaghan NJ, ~~and M.~~ Fenech M.~~,~~ A quantitative PCR method for measuring absolute telomere length. Biol Proced Online. 13 (2011) 3. doi: 10.1186/1480-9222-13-3.

[43] R.M. Cawthon, Telomere measurement by quantitative PCR. Nucleic Acids Res. **30**(10) (2002) e47. doi: 10.1093/nar/30.10.e47.

[44] ~~R.M.~~ Baron RM, ~~and D.A.~~ Kenny DA.~~,~~ The moderator-mediator variable distinction in social psychological research: conceptual, strategic, and statistical considerations. J Pers Soc Psychol. **51**(6) (1986)1173-82. doi: 10.1037//0022-3514.51.6.1173.

[45] ~~D.J.~~ Benjamin DJ, ~~J.O.~~ Berger JO, ~~M.~~ Johannesson M, ~~B.A.~~ Nosek, BA ~~E.J.~~ Wagenmakers EJ, ~~R.~~ Berk R, et al.~~, K.A. Bollen, B. Brembs, L. Brown, C. Camerer, D. Cesarini, C.D. Chambers, M. Clyde, T.D. Cook, P. De Boeck, Z. Dienes, A. Dreber, K. Easwaran, C. Efferson, E. Fehr, F. Fidler, A.P. Field, M. Forster, E.I. George, R. Gonzalez, S. Goodman, E. Green, D.P. Green, A.G. Greenwald, J.D. Hadfield, L.V. Hedges, L. Held, T. Hua Ho, H. Hoijtink, D.J. Hruschka, K. Imai, G. Imbens, J.P.A. Ioannidis, M. Jeon, J.H. Jones, M. Kirchler, D. Laibson, J. List, R. Little, A. Lupia, E. Machery, S.E. Maxwell, M. McCarthy, D.A. Moore, S.L. Morgan, M. Munafó, S. Nakagawa, B. Nyhan, T.H. Parker, L. Pericchi, M. Perugini, J. Rouder, J. Rousseau, V. Savalei, F.D. Schönbrodt, T. Sellke, B. Sinclair, D. Tingley, T. Van Zandt, S. Vazire, D. J. Watts, C. Winship, R.L. Wolpert, Y. Xie, C. Young, J. Zinman, V.E. Johnson.~~ Redefine statistical significance. Nat Hum Behav. 2(1) (2018) 6-10. doi: 10.1038/s41562-017-0189-z.

[46] ~~G.~~ Gigerenzer G. Mindless statistics. Journal of Socio-Economics, 33 (2004) 587-606. https://doi.org/10.1016/j.socec.2004.09.033

[47] SQRP, *Bilaga rapport 2020:1 Specialistvård: patienter som avslutats under 2019*. 2020.

[48] ~~A.~~ Szende A, Self-Reported Population Health: An International Perspective based on EQ-5D [Elektronisk resurs]. 2014.

[49] ~~L.~~ Hilton L, ~~S.~~ Hempel S, ~~B.A.~~ Ewing BA, ~~E.~~ Apaydin E, ~~L.~~ Xenakis L, ~~S.~~ Newberry S, et al. ~~, B. Colaiaco, A. Rualez Maher, R.M. Shanman, M.E. Sorbero, M.A. Maglione,~~ Mindfulness Meditation for Chronic Pain: Systematic Review and Meta-analysis. Ann Behav Med. **51**(2) (2017)199-213. doi: 10.1007/s12160-016-9844-2.

[50] ~~J.H.~~ Hammer JH, ~~N.G.~~ Wade NG, ~~and R.T.~~ Cragun RT.~~,~~ Valid assessment of spiritual quality of life with the WHOQOL-SRPB BREF across religious, spiritual, and secular persons: A psychometric study. Psychology of Religion and Spirituality, 12(4), 440–450. https://doi.org/10.1037/rel000026650.

[51] Bonelli RM, Koenig HG. Mental Disorders, Religion and Spirituality 1990 to 2010: A Systematic Evidence-Based Review. *J Relig Health* **52**, 657–673 (2013). https://doi.org/10.1007/s10943-013-9691-4

[52] Schnell T, Fuchs D, Hefti R. Worldview Under Stress: Preliminary Findings on Cardiovascular and Cortisol Stress Responses Predicted by Secularity, Religiosity, Spirituality, and Existential Search. *J Relig Health* **59**, 2969–2989 (2020). https://doi.org/10.1007/s10943-020-01008-5

[53~~1~~] ~~E.~~ Biccheri E, ~~N.~~ Roussiau N, ~~C.~~ Mambet-Doue C. ~~,~~ Fibromyalgia, Spirituality, Coping and Quality of Life. J Relig Health 55 (4) (2016), 1189-97. doi: 10.1007/s10943-016-0216-9.

[54~~2~~] ~~O.~~ Dedeli O, ~~. and G.~~ Kaptan G. ~~,~~ Spirituality and Religion in Pain and Pain Management. Health Psychol Res. **1**(3) (2013) e29. doi: 10.4081/hpr.2013.e29

[55~~3~~] ~~A.~~ Moreira-Almeida A, ~~, and H.G.~~ Koenig HG. ~~,~~ Religiousness and spirituality in fibromyalgia and chronic pain patients. Curr Pain Headache Rep. **12**(5) (2008) 327-32. doi: 10.1007/s11916-008-0055-9.

[56~~4~~] ~~B.~~ Gerdle B, ~~E.~~ Backryd E, ~~T.~~ Falkenberg T, ~~E.~~ Lundström E, ~~,~~ ~~B.~~ Ghafouri B. ~~,~~ Changes in inflammatory plasma proteins from patients with chronic pain associated with treatment in an interdisciplinary multimodal rehabilitation program - an explorative multivariate pilot study. Scand J Pain. **20**(1) (2019) 125-138. doi: 10.1515/sjpain-2019-0088.

[57~~5~~] ~~C.~~ Ahlgren C, ~~A.~~ Fjellman-Wiklund A, ~~K.~~ Hamberg K, ~~E.E.~~ Johansson EE, ~~B.M.~~ Stålnacke BM, The meanings given to gender in studies on multimodal rehabilitation for patients with chronic musculoskeletal pain – a literature review. Disability and Rehabilitation. **38**(23) (2016) 2255-2270. doi: 10.3109/09638288.2015.1127435.

[58~~6~~] ~~G.~~ Smirthwaite G, Tengelin E, ~~E.~~, Borrman ~~,~~ T. (O)jämställdhet i hälsa och vård. (rev uppl) Stockholm: Sveriges kommuner och regioner; 2014.

[59~~7~~] Statens beredning för medicinsk och social utvärdering. Behandling av långvariga smärttillstånd med fokus på kvinnor. En kartläggning av systematiska översikter. Vol. SBU-rapport nr 301. 2019: Stockholm: Statens beredning för medicinsk utvärdering (SBU), 2019.

[60~~58~~] Dowell ~~,~~ D~~.~~, ~~T.M.~~ Haegerich TM, ~~and R.~~ Chou R. ~~,~~ *CDC Guideline for Prescribing Opioids for Chronic Pain—United States, 2016.* JAMA, 2016. **315**(15): p. 1624-1645.

[61~~59~~] UN, *World drug report 2017*. 2017, Vienna: United Nations, Office on Drugs and Crime.

Formatted: English (United States)
Formatted: English (United States)
Formatted: English (United States)
Field Code Changed
Formatted: English (United States)
Formatted: English (United States)
Formatted: No underline, English (United States)
Formatted: No underline
Formatted: No underline, English (United States)
Formatted: No underline
Formatted: No underline, English (United States)
Formatted: No underline
Formatted: No underline, English (United States)
Formatted: Portuguese (Brazil)
Formatted: Swedish (Sweden)
Formatted: Swedish (Sweden)
Formatted: Swedish (Sweden)
Formatted: Swedish (Sweden)
Formatted: Swedish (Sweden)

[620] Statens beredning för medicinsk och social utvärdering, *Rehabilitering vid långvarig smärta: en systematisk litteraturöversikt.* Stockholm: Statens beredning för medicinsk och social utvärdering (SBU), 2010.

[631] Rosenberg , E.I., I. Genao I, I. Chen I, et al., *Complementary and alternative medicine use by primary care patients with chronic pain.* Pain Med, 2008. **9**(8): p. 1065-72.

[642] K. Preacher, & A. Hayes. SPSS and SAS procedures for estimating indirect effects in simple mediation models. Behavior Research Methods, Instruments, & Computers in Human Behavior. 36, (2004) 717-731.

[653] A.F. Hayes, Beyond Baron and Kenny: Statistical mediation analysis in the new millennium. *Communication Monographs. 76*(4) (2009) 408–420. https://doi.org/10.1080/03637750903310360

Formatted: Swedish (Sweden)

Formatted: Swedish (Sweden)

Formatted: Swedish (Sweden)

Formatted: Swedish (Sweden)

Karolinska Institutet

Response to Reviewers

Torkel Falkenberg
Karolinska Institutet
Dept. of Neurobiology, Care Sciences & Society
Alfred Nobels allé 23, 141 52 Huddinge
Sweden

PONE-D-23-08421

Exploring emotional well-being, personal, spiritual and religious beliefs and telomere length in chronic pain patients - A pilot study with cross-sectional design

May 2023,

Dear Editor,

Thank you for considering our manuscript for publication in PLOS ONE and please find our revisions and comments that addresses the points raised during the review process as described below.

Reviewer #1:

*The study explores a fascinating hypothesis, around the relationship between several psychological states and telomere length. I should note beforehand that my expertise lies in spirituality and psychology, not in biology, so there is a lot about this study that I cannot evaluate. This may also have led to some misunderstandings on my part, for which I hope the authors will forgive me.

-Thank you so much for appreciating our work.

*My main question around this study has to do with its rationale. A minor point on this is on page 4, when the authors state that 'sometimes TA is a signal for repair activity, or an indicator of a lack of such activity'. This immediately raised the question for me what then the relevance and validity of this measure is. Why is it included in this study? When is or isn't it an indicator for (non-)activity? Perhaps the authors can elaborate on this a little.

-Thank you for pointing this out. We have tried to clarify this and changed the text to: “Changes in TA enzyme levels have implications for telomere length and protection of the chromosomes [18, 19], making it suitable for examining more immediate effects in time of for example certain lifestyle factors or health-promoting behaviors [20].

*Also, from the introduction it doesn't become clear what the rationale behind the study is: Why do the authors expect TL and TA to relate to the mentioned PROs?

- We have made the rationale clearer in the introduction. In addition, we point to research that has linked TL to chronic pain and cellular ageing, whereas reports on pain and TA are lacking and hence the inclusion in this study in order to learn more about the possible role of TA levels and pain.

***What's more, from the methods section I understand that this study is part of a larger clinical trial on the effects of anthroposophical integrative care (AIC) or standard care (SC) on TA and TL, with the PROs being secondary outcome variables in this trial. What then, is the reason behind this current study?**

-Thank you for addressing this. Indeed, this is the baseline reporting (before care programs are provided). In the rationale we describe the need for research aiming to investigate correlations between emotional well-being, pain experiences and cellular aging in the group of chronic pain patients. Provided such correlations exists, this could underpin "new ways of developing and evaluating chronic pain interventions" for this group of patients.

***The research question of this manuscript is also not mentioned in the trial registration. Might it be that the authors were examining bivariate correlations to check their data, stumbled upon the associations reported here and decided to publish about it? If so, the authors should at least make this clear.**

-The reviewer is correct. At the time of the trial registration, we did not foresee baseline reporting as a self-standing paper. However, when confronted with the massive amount of data in this project, we realized the need for detailing the pain patients reported PROM's in relation to TA and TL before they were subjected to care programs, since such information may help the development and evaluation of current pain management.

***Assuming for now that the authors have a clear rationale for examining the research question in this manuscript, I have some further questions for clarification (in order of appearance, not in order of importance):**

*** What were the arguments for the inclusion and exclusion criteria? Specifically, the county councils and the comorbities (which are also reported much more specifically in the trial registration).**

-The idea was to be as pragmatic as possible and authentic to the irl clinical health care reality both patients and clinicians experience on a daily basis. Hence, we were aiming for external validity, rather than internal validity, and inclusion criterions were deliberately broad. The ambition was to describe this cohort of patients as correctly as possible, and hence their comorbidities were of interest, alongside with many other reported outcomes. The effect on all outcomes of the different care programs will be reported separately.

*** Why were the different parameters in the blood samples examined? What role do these play in the current study?**

-These are important to monitor since routine blood sample parameters beyond normal reference intervals could hypothetically affect our outcomes. However, at group level, this was not the case since all routine blood test results were within normal reference intervals according to Swedish standards at Karolinska University Laboratory. We do elaborate a little on this: "Interestingly, the blood samples did not deviate from normal reference intervals indicating that patients with severe and long-lasting pain

paradoxically appear "perfectly healthy". However, more complex proteomics or pro-inflammatory markers have been shown to deviate in chronic pain patients and correlate with other health aspects such as HAD scores [54]". The latter markers were not examined in this study.

*** Why were both partial correlations and linear regression analysis examined? Did the first influence which variables were included in the latter? Or was there some other reason?**

Thank you for noting this – indeed, the linear regression analyses are no longer reported in the paper due to the redundancy you describe, and we have thus removed that sentence form the statistical analysis description. Instead, we have added a description of the ANCOVA analysis result which is reported: "In addition, we used an analytic approach similar to Hassett et al [5], where the groups were divided into "high pain/low pain" and "high HAD score/low HAD score" (the group mean score was used as cut-off), and ANCOVA analyses adjusted for age were conducted with TL and TA as dependent variables."

*** Were no control variables included in the linear regression analysis, particularly gender and level of education? If so, why not? [I do want to complement the authors for controlling for multiple testing]**

We do not report linear regression analyses in this paper and have removed the description form the statistical analysis section (see above).

How were differences in age, pain duration and pain spread between the samples from the study sites handled?

*As we do not compare study sites in this study, we have not taken into account the differences in baseline variables in the study site samples in the analysis.

* What does it mean for the study that neither TL nor TA were correlated to any of the pain measures?

-We do elaborate on this: "Another finding is the absence of correlation between TL and the pain measures selected in this study. This is surprising since others have found statistically significant correlations between TL and pain measures [5, 21-24]. In contrast, TA had a positive relationship with pain spread, i.e. high TA was associated with higher pain spread. But again, this correlation is not large enough to reach a convincing significance level with the sample size we had available. This is ambiguous since TL is negatively associated with pain spread to a notable degree".

The fact that TL was not linked to pain measures but rather to existential measures is something we address in the summary: "In summary, what ultimately correlates with the genomic integrity within this sample of chronic pain patients is neither quantitative pain measures or scales, nor index numbers generated for health economy calculation purposes, but instead anxiety, depression and personal, spiritual and religious beliefs".

Our findings hence challenge the idea that the big focus of health care on the pain itself might not be what really is important for the well being of the patients, but rather their existential situation and experiences which seem also to be linked to vital parameters such as the longevity of their DNA.

Karolinska Institutet

*** Why was an ANCOVA performed for the HADS and not the WHOQOL?**

We agree, that it makes sense to run the same analysis and report results for WHOQOL-SRPB as well – we have now added this.

*** What theoretical considerations underlie the test for the mediation effect?**

Thank you for addressing this. As we state in the article, our mediation model is intuitive, but far from definitive and that one can argue that mediation is only demonstrated according to the original Baron and Kenny approach, but not according to more recent discussions, which would require an independent interaction term to be significant. However, we nevertheless think that this potential mediation might be interesting for other researchers to scrutinize in more detail. Given the explorative design of our study we hope the reviewer agree with this.

*** In the discussion section the authors discuss many findings that do not pertain to the research question. What is the purpose of this discussion?**

Thank you for pointing this out. We agree that some elaborations may seem a little out of scope. However, we take the opportunity in the discussion to address gender, pain management recommendations and the global opioid crisis since they are all dimensions which the typical chronic pain patient experience with implications for their individual care programs where emotional and spiritual dimensions are lacking.

*** What is the connection of the study with opioid use (p.22-23)? This was not included in the study was it?**

- We do report drug use in the result section and state in the discussion that: "despite the relation to global morbidity, mortality and doubtful long-term effectiveness, **24 % of the participants** in our sample of chronic pain patients had opioids prescribed for their pain". We think that this merits discussion of opioid use as well as the global crisis around pain management.

*** Overall, I think the authors present an interesting finding, based on acceptable methodology, that certainly warrants further study. However, the background of the study in insufficiently clear and needs a more sound rationale.**

- Thank you and we hope the changes and clarifications we have made have improved the manuscript to the reviewer's satisfaction.

Reviewer #2: Dear authors, thank you for your efforts. Your study covers a relevant topic and an important attempt to promote more holistic non-pharmacological treatments in chronic pain patients. Your methodology is sound (scales used, handling of biological materials) but the sample size is very small and relevant confounders have not been considered (tobacco and alcohol as stated by yourself, other lifestyle factors as exercise and nutrition, or patients medication specially anti-inflammatory drugs). This makes the results, specially the associations with TL/TA rather speculative and hypothetical.

Karolinska Institutet

Thank you for appreciating our efforts in this challenging study and we agree that research like this is needed to find new ways to improve existential care of patients in need, such as chronic pain patients given their large suffering.

We agree that the sample size is rather small, however given the complexity of this project with measures from cell to existential health it was not possible to enroll a larger patient basis. In the article we state that: “Because of its exploratory nature, no sample size was calculated but a minimum of 70 enrolled patients was strived for, which allows to document an effect size of r = .3 with 72% power and reaches a power of 80% with r = .32. These are the ranges of correlations that were deemed likely and clinically important”.

We do address this as a limitation in the discussion and also state that: “The size of the correlations we found were rather small, between r = .2 and .3. Because of the small sample size and the comparatively large number of correlations, inherent in an exploratory project such as this one, we would not want to draw any definitive conclusions”.

Hence, we agree that the findings of this exploratory project, notably the weak correlation to TL, need replication in larger clinical trials. In such future trials, the limitation of potential confounders apart from BMI, age or sex such as the one mentioned by the reviewer including tobacco or alcohol use should be considered.

We have added the limitations suggested by the reviewer to the limitation discussion: “Indeed, levels of exercise and nutrition and patients medication should also be considered”.

***Associations between spiritual und religious beliefs and mental health (anxiety and depression) are well documented and have been found in several studies and different contexts (pain, cardiac rehabilitation, coping with disease).**

-Thank you for addressing this, and we have extended the discussion to include an extension on this: “Studies have repeatedly established positive links between religion, spirituality and health where for example religious involvement is correlated with better mental health in the areas of depression, substance abuse, and suicide (reference 1). It has also been shown that cardiovascular and cortisol stress responses of a sample of university students can be predicted by their secularity, religiosity, spirituality, and existential search (reference 2)”.

1. Bonelli, R.M., Koenig, H.G. Mental Disorders, Religion and Spirituality 1990 to 2010: A Systematic Evidence-Based Review. *J Relig Health* **52**, 657–673 (2013). https://doi.org/10.1007/s10943-013-9691-4
2. Schnell, T., Fuchs, D. & Hefti, R. Worldview Under Stress: Preliminary Findings on Cardiovascular and Cortisol Stress Responses Predicted by Secularity, Religiosity, Spirituality, and Existential Search. *J Relig Health* **59**, 2969–2989 (2020). https://doi.org/10.1007/s10943-020-01008-5

---

6. PLOS authors have the option to publish the peer review history of their article (what does this mean?). If published, this will include your full peer review and any attached files.

If you choose “no”, your identity will remain anonymous but your review may still be made public.

Do you want your identity to be public for this peer review? For information about this choice, including consent withdrawal, please see our Privacy Policy.

Reviewer #1: **Yes:** Anja Visser

Reviewer #2: **Yes:** René Hefti M.D.

Thank you for your fine work in reviewing this manuscript and for your contributions to this research field.

Sincerely yours,

Torkel Falkenberg, on behalf of all the co-authors
