# Supplementary material for: Exploring emotional well-being, spiritual, religious and personal beliefs and telomere length in chronic pain patients—A pilot study with cross-sectional design
Source: PLoS One. 2024 Sep 4;19(9):e0308924. doi: 10.1371/journal.pone.0308924 (PMC11373805; doi:10.1371/journal.pone.0308924)
Supplement: Attachment — Submitted filename: Final Response to Reviewers .docx [file pone.0308924.s001.docx]

**Response to Reviewers**

Torkel Falkenberg

Karolinska Institutet

Dept. of Neurobiology, Care Sciences & Society

Alfred Nobels allé 23, 141 52 Huddinge

Sweden

**PONE-D-23-08421**

**Exploring emotional well-being, personal, spiritual and religious beliefs and telomere length in chronic pain patients - A pilot study with cross-sectional design**

May 2023,

**Dear Editor,

Thank you for considering our manuscript for publication in PLOS ONE and please find our revisions and comments that addresses the points raised during the review process as described below.**

Reviewer #1:

***The study explores a fascinating hypothesis, around the relationship between several psychological states and telomere length. I should note beforehand that my expertise lies in spirituality and psychology, not in biology, so there is a lot about this study that I cannot evaluate. This may also have led to some misunderstandings on my part, for which I hope the authors will forgive me.**

-Thank you so much for appreciating our work.

***My main question around this study has to do with its rationale. A minor point on this is on page 4, when the authors state that 'sometimes TA is a signal for repair activity, or an indicator of a lack of such activity'. This immediately raised the question for me what then the relevance and validity of this measure is. Why is it included in this study? When is or isn't it an indicator for (non-)activity? Perhaps the authors can elaborate on this a little.**

-Thank you for pointing this out. We have tried to clarify this and changed the text to: “Changes in TA enzyme levels have implications for telomere length and protection of the chromosomes [18, 19], making it suitable for examining more immediate effects in time of for example certain lifestyle factors or health-promoting behaviors [20].

***Also, from the introduction it doesn't become clear what the rationale behind the study is: Why do the authors expect TL and TA to relate to the mentioned PROs?**

- We have made the rationale clearer in the introduction. In addition, we point to research that has linked TL to chronic pain and cellular ageing, whereas reports on pain and TA are lacking and hence the inclusion in this study in order to learn more about the possible role of TA levels and pain.

***What's more, from the methods section I understand that this study is part of a larger clinical trial on the effects of anthroposophical integrative care (AIC) or standard care (SC) on TA and TL, with the PROs being secondary outcome variables in this trial. What then, is the reason behind this current study?**

-Thank you for addressing this. Indeed, this is the baseline reporting (before care programs are provided). In the rationale we describe the need for research aiming to investigate correlations between emotional well-being, pain experiences and cellular aging in the group of chronic pain patients. Provided such correlations exists, this could underpin “new ways of developing and evaluating chronic pain interventions” for this group of patients.

***The research question of this manuscript is also not mentioned in the trial registration. Might it be that the authors were examining bivariate correlations to check their data, stumbled upon the associations reported here and decided to publish about it? If so, the authors should at least make this clear.**

-The reviewer is correct. At the time of the trial registration, we did not foresee baseline reporting as a self-standing paper. However, when confronted with the massive amount of data in this project, we realized the need for detailing the pain patients reported PROM’s in relation to TA and TL before they were subjected to care programs, since such information may help the development and evaluation of current pain management.

***Assuming for now that the authors have a clear rationale for examining the research question in this manuscript, I have some further questions for clarification (in order of appearance, not in order of importance):**

*** What were the arguments for the inclusion and exclusion criteria? Specifically, the county councils and the comorbities (which are also reported much more specifically in the trial registration).**

-The idea was to be as pragmatic as possible and authentic to the irl clinical health care reality both patients and clinicians experience on a daily basis. Hence, we were aiming for external validity, rather than internal validity, and inclusion criterions were deliberately broad. The ambition was to describe this cohort of patients as correctly as possible, and hence their comorbidities were of interest, alongside with many other reported outcomes. The effect on all outcomes of the different care programs will be reported separately.

*** Why were the different parameters in the blood samples examined? What role do these play in the current study?**

-These are important to monitor since routine blood sample parameters beyond normal reference intervals could hypothetically affect our outcomes. However, at group level, this was not the case since all routine blood test results were within normal reference intervals according to Swedish standards at Karolinska University Laboratory. We do elaborate a little on this: “Interestingly, the blood samples did not deviate from normal reference intervals indicating that patients with severe and long-lasting pain

paradoxically appear “perfectly healthy”. However, more complex proteomics or pro-inflammatory markers have been shown to deviate in chronic pain patients and correlate with other health aspects such as HAD scores [54]”. The latter markers were not examined in this study.

*** Why were both partial correlations and linear regression analysis examined? Did the first influence which variables were included in the latter? Or was there some other reason?**

Thank you for noting this – indeed, the linear regression analyses are no longer reported in the paper due to the redundancy you describe, and we have thus removed that sentence form the statistical analysis description. Instead, we have added a description of the ANCOVA analysis result which is reported: “In addition, we used an analytic approach similar to Hassett et al [5], where the groups were divided into “high pain/low pain” and “high HAD score/low HAD score” (the group mean score was used as cut-off), and ANCOVA analyses adjusted for age were conducted with TL and TA as dependent variables.”

*** Were no control variables included in the linear regression analysis, particularly gender and level of education? If so, why not? [I do want to complement the authors for controlling for multiple testing]**

We do not report linear regression analyses in this paper and have removed the description form the statistical analysis section (see above).

**How were differences in age, pain duration and pain spread between the samples from the study sites handled?**

*As we do not compare study sites in this study, we have not taken into account the differences in baseline variables in the study site samples in the analysis.

* What does it mean for the study that neither TL nor TA were correlated to any of the pain measures?

-We do elaborate on this: “Another finding is the absence of correlation between TL and the pain measures selected in this study. This is surprising since others have found statistically significant correlations between TL and pain measures [5, 21-24]. In contrast, TA had a positive relationship with pain spread, i.e. high TA was associated with higher pain spread. But again, this correlation is not large enough to reach a convincing significance level with the sample size we had available. This is ambiguous since TL is negatively associated with pain spread to a notable degree”.

The fact that TL was not linked to pain measures but rather to existential measures is something we address in the summary: “In summary, what ultimately correlates with the genomic integrity within this sample of chronic pain patients is neither quantitative pain measures or scales, nor index numbers generated for health economy calculation purposes, but instead anxiety, depression and personal, spiritual and religious beliefs”.

Our findings hence challenge the idea that the big focus of health care on the pain itself might not be what really is important for the well being of the patients, but rather their existential situation and experiences which seem also to be linked to vital parameters such as the longevity of their DNA.

*** Why was an ANCOVA performed for the HADS and not the WHOQOL?**

We agree, that it makes sense to run the same analysis and report results for WHOQOL-SRPB as well – we have now added this.

*** What theoretical considerations underlie the test for the mediation effect?**

Thank you for addressing this. As we state in the article, our mediation model is intuitive, but far from definitive and that one can argue that mediation is only demonstrated according to the original Baron and Kenny approach, but not according to more recent discussions, which would require an independent interaction term to be significant. However, we nevertheless think that this potential mediation might be interesting for other researchers to scrutinize in more detail**.** Given the explorative design of our study we hope the reviewer agree with this.

*** In the discussion section the authors discuss many findings that do not pertain to the research question. What is the purpose of this discussion?**

Thank you for pointing this out. We agree that some elaborations may seem a little out of scope. However, we take the opportunity in the discussion to address gender, pain management recommendations and the global opioid crisis since they are all dimensions which the typical chronic pain patient experience with implications for their individual care programs where emotional and spiritual dimensions are lacking.

*** What is the connection of the study with opioid use (p.22-23)? This was not included in the study was it?**

- We do report drug use in the result section and state in the discussion that: “despite the relation to global morbidity, mortality and doubtful long-term effectiveness, **24 % of the participants** in our sample of chronic pain patients had opioids prescribed for their pain”. We think that this merits discussion of opioid use as well as the global crisis around pain management.

*** Overall, I think the authors present an interesting finding, based on acceptable methodology, that certainly warrants further study. However, the background of the study in insufficiently clear and needs a more sound rationale.**

- Thank you and we hope the changes and clarifications we have made have improved the manuscript to the reviewer’s satisfaction.

**Reviewer #2: Dear authors, thank you for your efforts. Your study covers a relevant topic and an important attempt to promote more holistic non-pharmacological treatments in chronic pain patients. Your methodology is sound (scales used, handling of biological materials) but the sample size is very small and relevant confounders have not been considered (tobacco and alcohol as stated by yourself, other lifestyle factors as exercise and nutrition, or patients medication specially anti-inflammatory drugs). This makes the results, specially the associations with TL/TA rather speculative and hypothetical.**

Thank you for appreciating our efforts in this challenging study and we agree that research like this is needed to find new ways to improve existential care of patients in need, such as chronic pain patients given their large suffering.

We agree that the sample size is rather small, however given the complexity of this project with measures from cell to existential health it was not possible to enroll a larger patient basis. In the article we state that: “Because of its exploratory nature, no sample size was calculated but a minimum of 70 enrolled patients was strived for, which allows to document an effect size of r = .3 with 72% power and reaches a power of 80% with r = .32. These are the ranges of correlations that were deemed likely and clinically important”.

We do address this as a limitation in the discussion and also state that: “The size of the correlations we found were rather small, between r = .2 and .3. Because of the small sample size and the comparatively large number of correlations, inherent in an exploratory project such as this one, we would not want to draw any definitive conclusions”.

Hence, we agree that the findings of this exploratory project, notably the weak correlation to TL, need replication in larger clinical trials. In such future trials, the limitation of potential confounders apart from BMI, age or sex such as the one mentioned by the reviewer including tobacco or alcohol use should be considered.

We have added the limitations suggested by the reviewer to the limitation discussion: “Indeed, levels of exercise and nutrition and patients medication should also be considered”.

***Associations between spiritual und religious beliefs and mental health (anxiety and depression) are well documented and have been found in several studies and different contexts (pain, cardiac rehabilitation, coping with disease).**

-Thank you for addressing this, and we have extended the discussion to include an extension on this: “Studies have repeatedly established positive links between religion, spirituality and health where for example religious involvement is correlated with better mental health in the areas of depression, substance abuse, and suicide (reference 1). It has also been shown that cardiovascular and cortisol stress responses of a sample of university students can be predicted by their secularity, religiosity, spirituality, and existential search (reference 2)”.

1. Bonelli, R.M., Koenig, H.G. Mental Disorders, Religion and Spirituality 1990 to 2010: A Systematic Evidence-Based Review. *J Relig Health* **52**, 657–673 (2013). <https://doi.org/10.1007/s10943-013-9691-4>
2. Schnell, T., Fuchs, D. & Hefti, R. Worldview Under Stress: Preliminary Findings on Cardiovascular and Cortisol Stress Responses Predicted by Secularity, Religiosity, Spirituality, and Existential Search. *J Relig Health* **59**, 2969–2989 (2020). https://doi.org/10.1007/s10943-020-01008-5

6. PLOS authors have the option to publish the peer review history of their article ([what does this mean?](https://eur01.safelinks.protection.outlook.com/?url=https%3A%2F%2Fjournals.plos.org%2Fplosone%2Fs%2Feditorial-and-peer-review-process%23loc-peer-review-history&data=05%7C01%7Ctorkel.falkenberg%40ki.se%7C78e2dc5df30c4e2c3f6b08db4677aa58%7Cbff7eef1cf4b4f32be3da1dda043c05d%7C0%7C0%7C638181252142856983%7CUnknown%7CTWFpbGZsb3d8eyJWIjoiMC4wLjAwMDAiLCJQIjoiV2luMzIiLCJBTiI6Ik1haWwiLCJXVCI6Mn0%3D%7C3000%7C%7C%7C&sdata=0bezHxVYgYSusldm9vD5%2F4bdyJ5Zb8EUbVSPV%2BZ0wts%3D&reserved=0)). If published, this will include your full peer review and any attached files.

If you choose “no”, your identity will remain anonymous but your review may still be made public.

**Do you want your identity to be public for this peer review?** For information about this choice, including consent withdrawal, please see our [Privacy Policy](https://eur01.safelinks.protection.outlook.com/?url=https%3A%2F%2Fwww.plos.org%2Fprivacy-policy&data=05%7C01%7Ctorkel.falkenberg%40ki.se%7C78e2dc5df30c4e2c3f6b08db4677aa58%7Cbff7eef1cf4b4f32be3da1dda043c05d%7C0%7C0%7C638181252142856983%7CUnknown%7CTWFpbGZsb3d8eyJWIjoiMC4wLjAwMDAiLCJQIjoiV2luMzIiLCJBTiI6Ik1haWwiLCJXVCI6Mn0%3D%7C3000%7C%7C%7C&sdata=8antI2C1XEmqEd%2FC4nL6OqOyGgLQzjvJMoyFenc%2FiWs%3D&reserved=0).

Reviewer #1: **Yes:** Anja Visser

Reviewer #2: **Yes:** René Hefti M.D.

Thank you for your fine work in reviewing this manuscript and for your contributions to this research field.

Sincerely yours,

Torkel Falkenberg, on behalf of all the co-authors
