# Supplementary material for: Exploring emotional well-being, spiritual, religious and personal beliefs and telomere length in chronic pain patients—A pilot study with cross-sectional design
Source: PLoS One. 2024 Sep 4;19(9):e0308924. doi: 10.1371/journal.pone.0308924 (PMC11373805; doi:10.1371/journal.pone.0308924)
Supplement: Attachment — Submitted filename: Response to Reviewers.docx [file pone.0308924.s003.docx]

**Response to reviewers**

Torkel Falkenberg

Karolinska Institutet

Dept. of Neurobiology, Care Sciences & Society

Alfred Nobels allé 23, 141 52 Huddinge

Sweden

**PONE-D-23-08421**

**Exploring emotional well-being, spiritual, religious and personal beliefs and telomere length in chronic pain patients - A pilot study with cross-sectional design**

January 2024,

**Dear Editor,

Thank you again for considering our manuscript for publication in PLOS ONE and please find a revised version of the manuscript that addresses the points raised during the latest review process as described below, with our answers in bold.**

**Reviewer #1:**

*Thank you for carefully addressing the comments in your reply. Though I recognize that you have made changes in the manuscript, I would have preferred them to be a bit more elaborate to really help the reader understand why this study was conducted and is relevant and - with that - what they can learn from it. In particular, I want to ask the authors again to be more explicit about the relationship of this study to the clinical trial and the ad-hoc nature of this part of the study. I ask this mostly from a pedagogical perspective: It is helpful for readers to understand how this research process went and what struck you about the data that prompted further exploration.

**-One of our main interests lie within the field of spirituality. More specifically in the connection between psychospiritual factors and biological health. The overall aim of the prospective *clinical trial* was to achieve a better understanding of the possible effects of, and useful measure for evaluating, two different multimodal treatment interventions for pain. What might not have been entirely clear on clinicaltrials.gov was the explicit difference between those two care programs evaluated; one was conventional/multimodal and the other had a distinct spiritual approach.**

**The aim of *this* study was to explore correlations of PROM’s and cellular ageing (telomere length [TL] and telomerase activity [TA]) amongst patients with chronic non-malignant pain (with some of the PROM’s relating to psychospiritual health). That is the philosophical interrelatedness between the clinical trial and this study. Moreover, as mentioned in the first rebuttal letter, we**

**did not foresee the massive amount of data and realized the need for a baseline study in order to evaluate TA and TL in relation to PROMS, before adding the longitudinal perspective and evaluating the care programs. That is the pragmatic “why”.**

**We acknowledge that it might be uncommon to combine psychospiritual and genomic perspectives, -seemingly very different areas. However, in the context of chronic pain, these are two areas lacking research. That’s the relevance. In addition it would naturally be rather exciting if a connection between the spiritual and material dimensions could be seen. As to what can be learned, we feel that it has been sufficiently elaborated upon under “conclusion” and “implications for further studies”. In an attempt to be more explicit and to facilitate an understanding, a short introduction has been added to the very beginning:**

**“This study is an exploration of correlations between baseline parameters obtained from a larger, prospective clinical trial. The study spans over broad fields of knowledge, from psychospirituality to genomics. However, in the context of chronic pain, both of these fields are under-researched, which motivates the present study. In addition, this study has an explicit interest in exploring possible links between these two areas of research.”**

*On this second inspection, the results of the study seem to become less clear to me than they were. One of the reasons for this is that the authors do not provide interpretations of all the analyses displayed in the results section. It is left to the reader to interpret many of the tables and draw conclusions from them. That is not helpful, given that you present this data for a reason and it had a part to play in your decision-making about analyses and your answer to the research question. Present these interpretations to the reader.

**-Thank you for addressing this. Indeed, we agree that the results can be interpreted more clearly to help the reader. While we have chosen to focus the results section on the *presentation* of results, and placed the interpretations in the discussion section, we have carefully improved the phrasing and referring to results tables throughout the texts. Hopefully this improvement of the stringency will facilitate reader understanding.**

*When the ANCOVA results are presented, only the analysis with the HADS is mentioned, but the results of the WHOQOL are also presented, which is confusing.

**-Thank you for noting this, we have now added mention of the ““highWHOQOL-SRPB/lowWHOQOL-SRPB” analysis as well.**

*Here, the reader also really needs to see how the authors interpret the different results between the partial correlations and the ANCOVA, so they can understand why the authors push on with the mediation analysis regardless of the negative results of the ANCOVA.

**-It is an astute observation that these two analyses do not follow from each other. We intended them to complement each other, and we have added half a sentence to this effect at the beginning of the mediation analysis “Since the ANCOVA-analysis with dichotomized SRPB scores might have collapsed variance, and as (…)”, in addition to the following sentences on p17: “This was in order to assess whether dichotomised variables of pain, anxiety/depression, and SRPB would yield additional information about the associations with TL and TA, to what can be found through correlations with continuous variables. However, no associations were detected in this additional analysis”.**

*When the mediation analysis is presented, it is suggested that it is obvious from the results and theory that anxiety would mediate the relationship between spirituality and TL. However, I don't see an indication in the results nor in the introduction section (theoretical framework) that would suggest this. In the discussion section the authors also put this into question again. So this really needs some sentences of explanation as well. Perhaps one of my own publications can be helpful here (Visser, A., de Jager Meezenbroek, E. C., & Garssen, B. (2018). Does spirituality reduce the impact of somatic symptoms on distress in cancer patients? Cross-sectional and longitudinal findings. Social Science and Medicine, 214(August), 57–66. <https://doi.org/10.1016/j.socscimed.2018.08.012>), but I'm sure there are also other publications that discuss this issue. In the discussion section even mindfulness is included in the mediation hypothesis, which was not examined in the study. (it also seems the mediation effect is discussed twice in subsequent paragraphs, which is redundant).

**To make this connection more clear for the reader, we have added a short paragraph to the introduction (row 247-252), also quoting some references for interested readers to follow up. The new references have been added as nr 29-33 in the reference list. Since this mediation analysis is really only a serendipitous finding, we did not want to place more emphasis on it by adding still more text in the discussion**. **We hope that the reviewer will agree with this reasoning.**

*A more minor thing that keeps puzzling me about the results is the difference between the two sites. If you didn't think they were of importance, why then did you analyse and present them? And now that you have analysed and presented them, how do you interpret these differences and what conclusions do you draw from that in relation to your research question?

**-Thank you for raising this question. We agree that it seems obsolete in relation to this study and the table has been removed. Since it is a baseline study with the same inclusion criteria for both sites, the differences are irrelevant. The data might, however, be displayed and interpreted in coming articles with longitudinal perspectives, where it may have relevance.**

*So, notwithstanding my interest in this study, I still feel the authors should be more precise in their exhibition of the study and its result.

**-Well taken. We hope that our adjustments and revisions according to your suggestions has elevated and clarified the work.**

**Reviewer #2:** 

Dear authors , thank you for revising the manuscript and addressing my comments. By having a second look at the manuscript I have to make the following statements:

1. English language must be improved. The manuscript has to be revised by a native English speaker (or a professional). I made some few comments to give you examples. But it is a general topic for the whole manuscript.

**-Thank you for pointing this out. The manuscript has now been revised and grammatically corrected by a native English speaker.**

2. Please address my further comments in the attached document (see manuscript with track changes)

**-All the comments from the pdf file have been addressed in the word file with changes made accordingly.**

3. The paragraph "limitations" (usually "strengths and limitations") must be rewritten. It is a disordered mixture of limitations and possible strengths. First mention strengths and then limitations.

**-Thank you. We have now restructured the “strengths and limitations” section and moved text from that section which was not clearly related to strengths and limitations to a new sub heading “implications for further studies”.**

Many thanks for your fine work in reviewing this manuscript and also for your contributions to this research field.

Sincerely yours,

Torkel Falkenberg, on behalf of all the co-authors
