# Supplementary material for: Exploring emotional well-being, spiritual, religious and personal beliefs and telomere length in chronic pain patients—A pilot study with cross-sectional design
Source: PLoS One. 2024 Sep 4;19(9):e0308924. doi: 10.1371/journal.pone.0308924 (PMC11373805; doi:10.1371/journal.pone.0308924)
Supplement: Attachment — Submitted filename: Response to Reviewers.docx [file pone.0308924.s004.docx]

**Response to reviewers**

Torkel Falkenberg

Karolinska Institutet

Dept. of Neurobiology, Care Sciences & Society

Alfred Nobels allé 23, 141 52 Huddinge

Sweden

**PONE-D-23-08421**

**Exploring emotional well-being, spiritual, religious and personal beliefs and telomere length in chronic pain patients - A pilot study with cross-sectional design**

July 2024,

**Dear Editor,

Thank you once again for considering our manuscript for publication in PLOS ONE and please find a revised version of the manuscript that addresses the points raised during this latest review process as described below, with our answers in bold.**

**Reviewer #1:**

* Thank for carefully addressing all of the comments. The rationale of the study is now sufficiently clear.

**-Thank you for the reviewing and help with elevating our work**

**Reviewer #3:** 

The findings from this pilot study are very interesting. While the paper has improved from the initial submission, I have several concerns that the authors should consider.


1) I do not think the introduction/background adequately justifies the current study. What is the motivation beyond these topics not being previously investigated? Why might religion/ spirituality impact telomere length among those with chronic pain? Why might religion/ spirituality be mediated by mental health in this population? Despite space limitations, some elaboration is necessary.

I suggest reviewing the following for a general framework:

Hill, T. D., Ellison, C. G., Burdette, A. M., Taylor, J., & Friedman, K. L. (2016). Dimensions of religious involvement and leukocyte telomere length. Social Science & Medicine, 163, 168-175.

Page, R. L., Peltzer, J. N., Burdette, A. M., & Hill, T. D. (2020). Religiosity and health: A holistic biopsychosocial perspective. Journal of holistic nursing, 38(1), 89-101.

Handbook of Religion and Health- Koenig, VanderWeele, Peteet 2024 (There is a section on religion and biological functioning).

I also suggest reviewing the following for information on how religion may work among those with health problems:

Idler, E. L., & Kasl, S. V. (1992). Religion, disability, depression, and the timing of death. American journal of Sociology, 97(4), 1052-1079.

Hill, T. D., Burdette, A. M., Taylor, J., & Angel, J. L. (2016). Religious attendance and the mobility trajectories of older Mexican Americans: An application of the growth mixture model. Journal of Health and Social Behavior, 57(1), 118-134.

**-We are grateful for this advice. We have reviewed the studies indicated by the reviewers and included four of them plus an additional one that may help clarify the definitional problems. We added two paragraphs to the MS (lines 122 to 140). We hope that this clarifies the purpose better. Also, it should be noted that our research question was rather broad and because of some ethical concerns about the load of questions to be given to the patients we were unable to implement a more differentiated measurement approach to spirituality and religiosity.**

2) Did the authors conduct additional analyses to exam individual aspects of the religion/ spirituality scale with the focal variables? Given that the most significant findings center around this scale, additional analyses may be insightful.

**-No, we did not. We already added one additional analysis, the mediation model, to those stipulated by our protocol and we mentioned this. Since already this additional analysis met with some concerns by other reviewers in previous rounds of reviews we thought it not advisable to go into further details. Apart from that, we had no further a-priori hypotheses about specific hypotheses of how aspects of mindfulness or of the spiritual well being module of the WHOQol would be related to our potential outcome variables. As this was only one aspect of our study, we did not feel justified to further explore the data set. Exploration of such data always comes with the danger of capitalization of chance, especially if no a-priori hypotheses have been formulated. Hence, we would rather not explore this data-set further but rather develop a new study with more focused hypotheses.**

3) I also suggest strengthening the discussion by elaborating on what we can take from these findings. The current discussion section is somewhat vague and would benefit from examining previous research on religion/spirituality and biological functioning.

**-Thank you for this suggestion. We have added a paragraph where we added some previous findings and strengthened the discussion and conclusions accordingly. We hope, this makes the manuscript acceptable.**

Many thanks for your fine work in reviewing this manuscript and also for your contributions to this research field.

Sincerely yours,

Torkel Falkenberg, on behalf of all the co-authors
